# Co-Crystal Structure-Guided Optimization of Dual-Functional Small Molecules for Improving the Peroxygenase Activity of Cytochrome P450BM3

**DOI:** 10.3390/ijms23147901

**Published:** 2022-07-18

**Authors:** Xiangquan Qin, Yiping Jiang, Jie Chen, Fuquan Yao, Panxia Zhao, Longyi Jin, Zhiqi Cong

**Affiliations:** 1 Department of Chemistry, Yanbian University, Yanji 133002, China; qxiangquan@163.com; 2 CAS Key Laboratory of Biofuels, Shandong Provincial Key Laboratory of Synthetic Biology, Qingdao Institute of Bioenergy and Bioprocess Technology, Chinese Academy of Sciences, Qingdao 266101, China; jiangyp@qibebt.ac.cn (Y.J.); chen_jie@qibebt.ac.cn (J.C.); yaofq@qibebt.ac.cn (F.Y.); zhaopx@qibebt.ac.cn (P.Z.); 3 University of Chinese Academy of Sciences, Beijing 100049, China

**Keywords:** cytochrome P450, peroxygenase, dual-functional small molecule, hydrogen peroxide, docking simulation

## Abstract

We recently developed an artificial P450–H_2_O_2_ system assisted by dual-functional small molecules (DFSMs) to modify the P450BM3 monooxygenase into its peroxygenase mode, which could be widely used for the oxidation of non-native substrates. Aiming to further improve the DFSM-facilitated P450–H_2_O_2_ system, a series of novel DFSMs having various unnatural amino acid groups was designed and synthesized, based on the co-crystal structure of P450BM3 and a typical DFSM, N-(ω-imidazolyl)-hexanoyl-L-phenylalanine, in this study. The size and hydrophobicity of the amino acid residue in the DFSM drastically affected the catalytic activity (up to 5-fold), stereoselectivity, and regioselectivity of the epoxidation and hydroxylation reactions. Docking simulations illustrated that the differential catalytic ability among the DFSMs is closely related to the binding affinity and the distance between the catalytic group and heme iron. This study not only enriches the DFSM toolbox to provide more options for utilizing the peroxide-shunt pathway of cytochrome P450BM3, but also sheds light on the great potential of the DFSM-driven P450 peroxygenase system in catalytic applications based on DFSM tunability.

## 1. Introduction

**Figure 1:**
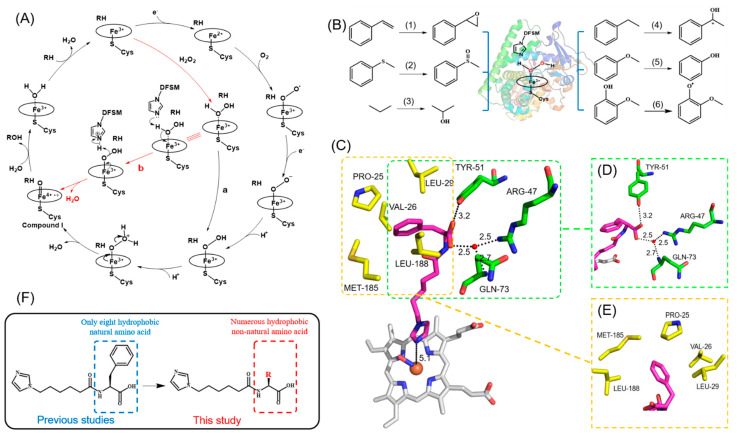
(**A**) The catalytic cycle of P450 monooxygenase and the proposed mechanism of DFSM-activated peroxide-shunt pathway to generate the artificial P450BM3 peroxygenase. a: The peroxide-shunt pathway without the addition of the DFSMs; b: DFSM-activated peroxide-shunt pathway. (**B**) Typical reactions catalyzed by the DFSM-facilitated P450BM3-H_2_O_2_ system: 1. styrene epoxidation; 2. thioanisole sulfoxidation; 3 and 4. hydroxylation of alkanes and aromatic compounds, 5. demethylation of aromatic ethers; 6. one-electron oxidation of phenol. (**C**) Co-crystal structure of P450BM3 and the DFSM Im-C6-Phe (PDB No. 7EGN [42]). (**D**) Hydrogen bond networks between Im-C6-Phe and P450BM3. (**E**) Hydrophobic interactions between the anchor group of Im-C6-Phe and P450BM3. (**F**) Co-crystal structure-guided optimization of the DFSM by introducing hydrophobic unnatural amino acids.

Cytochrome P450 enzymes (P450s) are a superfamily of heme proteins involved in drug metabolism, xenobiotic detoxification, and steroid biosynthesis [1,2]. P450s are promising biocatalysts for organic synthesis, drug discovery, and bioremediation because of their versatile catalytic oxyfunctionalizations of a variety of substrates [3,4,5,6,7,8,9,10,11,12,13,14,15,16,17]. However, one of the limitations of applying P450s as in vitro biocatalysts is that most P450s require reduced nicotinamide cofactors NAD(P)H and a complex system of electron transport partners (redox partners) to activate molecular oxygen for the formation of active species. Although the peroxide-shunt pathway is an alternative approach to avoid the consumption of these expensive cofactors, this pathway is inefficient for typical P450s (Figure 1A) [18,19]. In fact, there are only a few native P450s belonging to the CYP152 family that are capable of efficiently using H_2_O_2_ to perform their catalytic functions [20,21,22]. Considering the significant potential of peroxygenases as practical biocatalysts [23,24,25,26], many efforts have been made to develop a peroxide-driven P450 system [27,28,29,30,31].

By mimicking the molecular structure and catalytic mechanism of natural peroxygenase and peroxidase [32,33], we recently developed a unique P450–H_2_O_2_ system by employing an exogenous fatty acyl amino acid equipped with a terminal imidazolyl (called a dual-functional small molecule, DFSM) to activate the peroxide-shunt pathway of P450BM3. The typical DFSM, such as *N*-(ω-imidazolyl)-hexanoyl-l-phenylalanine (Im-C6-Phe), was thought to bind with P450BM3 by the acyl amino acid, as the anchor group and its imidazolyl moiety serves as the acid–base catalyst to facilitate H_2_O_2_ activation (shown as the red arrow pathway in Figure 1A) [34,35,36,37,38,39,40,41]. This system uses various P450BM3 variants to efficiently catalyze a variety of monooxygenation and oxidation reactions towards non-native substrates, e.g., styrene epoxidation, thioanisole sulfoxidation, hydroxylation of alkanes and aromatic compounds, demethylation of aromatic ethers, as well as one-electron oxidation of phenol and aromatic amines (Figure 1B). In these reactions, the catalytic activity and selectivity were found to be dependent on the presence of the DFSMs. We, therefore, reasoned that the catalytic capability of the DFSM-facilitated P450–H_2_O_2_ system could be further enhanced by fine-tuning the structure of the DFSMs.

The crystallographic studies revealed the unusual binding mode of the DFSM Im-C6-Phe to P450BM3, which is distinct from those of the previously reported natural fatty acid substrate and its analogues, namely, there is a clearly hydrophobic interaction except for the hydrogen-bond interactions between the DFSM and protein [42]. As shown in Figure 1C–E, the terminal carboxyl group directly forms a hydrogen bond with Tyr51 while interacting with Arg47 and Gln73 through the water-mediated hydrogen-bond networks [43,44,45]. Notably, the benzyl moiety of Im-C6-Phe is buried in a hydrophobic pocket composed of Pro25, Val26, Leu29, Met185, and Leu188, suggesting the potential contribution of the hydrophobic interactions to DFSM binding. Thus, we recognized that we could modulate the binding affinity of the DFSM to P450 by tuning its hydrophobic effect, which might affect the catalytic efficiency of the DFSM-facilitated P450 peroxygenase system. However, natural amino acids were the only source of synthetic blocks as anchoring groups of the DFSMs in previous reports [34,46]. The fact that there are only eight hydrophobic natural amino acids hindered us from systematically estimating the hydrophobic effect on the catalytic activity and selectivity of the DFSM-facilitated P450 peroxygenase. In the last few decades, a myriad of unnatural amino acids have been constructed and widely used in pharmaceutical, food, and chiral synthesis [47,48,49]. The involvement of unnatural amino acids could provide infinite possibilities for designing and fabricating novel DFSMs. Herein, we report the design and synthesis of new DFSMs using unnatural amino acids that have various hydrophobic side-chain groups and evaluate their catalytic efficiency in the epoxidation and hydroxylation reactions, as well as scrutinize their hydrophobic interactions with P450 by molecular docking simulations (Figure 1F).

## 2. Results and Discussion

### 2.1. Design and Synthesis of the DFSMs Equipped with Unnatural Amino Acids

**Scheme 1:**
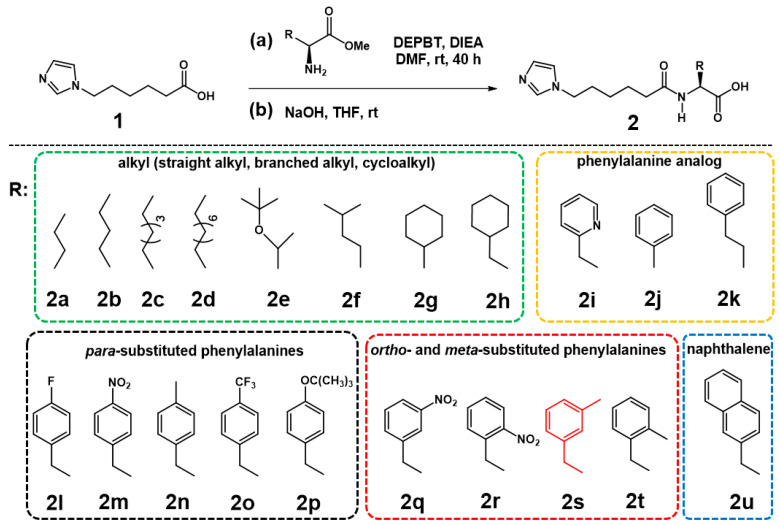
Synthesis of the DFSMs having various of unnatural amino acids. (a) Amino acid methyl ester hydrochloride (1.1 eq), DEPBT (1.1 eq), DIEA (2 eq), DMF, rt, 40h. (b) NaOH (1 M), rt, 1 h.

Twenty-one hydrophobic unnatural amino acids were chosen as anchoring groups of the DFSMs to investigate how enzymatic catalysis is modulated by the size and hydrophobicity of the DFSMs. According to the nature of the functional group, the side chains of these amino acids can be roughly divided into two classes, i.e., alkyl groups and aromatic groups. The former includes straight chains (**2a**–**2d**), branched chains (**2e**, **2f**), and cycloalkyls (**2g**, **2h**). The latter includes phenylalanine analogues (**2i**–**2k**), *para*-substituted phenylalanines (**2l**–**2p**), *ortho*- and *meta*-substituted phenylalanines (**2q**–**2t**), and naphthalene methylene (**2u**) (Scheme 1). The DFSMs were then synthesized by the amidation condensation and subsequent hydrolysis following a modified approach of a previous report [50]. Given the excellent activity of Im-C6-Phe shown in most cases, *N*-(6-imidazolyl) hexanoic acid (Im-C6-COOH) was chosen as the starting material to react with unnatural amino acid methyl esters (**1**), using 3-(diethoxy-phosphoryloxy)-1,2,3-benzotriazin-4(3*H*)-one (DEPBT) as the coupling agent instead of 1-hydroxybenzotriazole (HOBT). This is due to better chirality retention of DEPBT in comparison with HOBT. In fact, we have even observed that the partial racemization of the amino acid moiety easily occurred when we performed the gram-scale preparation of Im-C6-Phe with HOBT as the coupling agent (unpublished data), whereas alkaline hydrolysis did not influence the optical purity of the final DFSM products under proper conditions. Thus, 21 DFSMs, having hydrophobic unnatural amino acids (**2a**–**2u**), were obtained with total yields over a range of 42–88% (Scheme S2).

### 2.2. Effect of the DFSMs on the H_2_O_2_-Dependent Epoxidation of P450BM3

**Figure 2:**
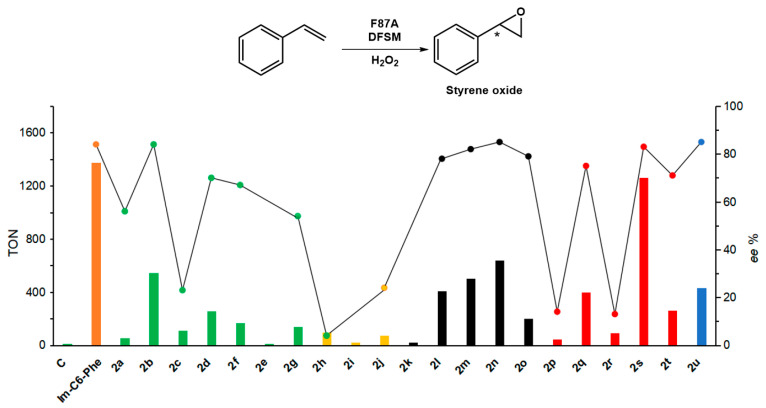
H_2_O_2_-dependent epoxidation of styrene catalyzed by P450BM3 in the absence/presence of DFSMs. *ee*, enantiomeric excess (%) of (*R*)-styrene oxide determined by chiral GC and was calculated by ((*R*)-styrene oxide − (*S*)-styrene oxide)/((*R*)-styrene oxide + (*S*)-styrene oxide) according to the corresponding peak area. TON: Turnover numbers were estimated for 30-min reactions. In this study, TON is also the maximum number of substrate molecules that were catalyzed by one enzyme molecule, since the system is inactivated by the high concentration of H_2_O_2_ within 30 min. The TON and *ee*% values are presented as bar charts and stacked line charts, respectively. *: The chiral carbon atom.

With the new DFSMs having hydrophobic unnatural amino acid groups in hand, we first evaluated their effect on the epoxidation of styrene catalyzed by the F87A mutant of P450BM3 (Figure 2). The F87A mutant gave a low catalytic turnover number (TON) of 12 in the absence of the DFSMs, which is consistent with our previous result [34,37]. As expected, the epoxidation activity was improved in varying degrees upon the addition of the DFSMs. When the DFSMs that had unnatural amino acid groups with straight alkyl side chains (**2a**–**2d**) were used, the catalytic TON almost presented a parabolic change with the increase of the number of carbon atoms of the alkyl group. The DFSM **2b** showed the best catalytic TON among four examined small molecules. Based on the above results, we speculate that the bulk of the residue is insufficient to fill the hydrophobic region of P450BM3, causing low peroxygenase activity. Meanwhile, the flexible alkyl backbone would not be confined to the hydrophobic region if the alkyl chain were to be excessively extended. The undesired alkyl chain might occupy the substrate channel and hinder substrate binding. Similarly, the lower catalytic activity of branched residues (**2e**, **2f**) and cyclohexane residues (**2g**, **2h**) further confirm this deduction. Subsequently, the activities of the phenylalanine analogues 3-(2-pyridyl)-l-alanine (**2i**), l-phenyl glycine (**2j**), and l-homophenylalanine (**2k**) were characterized, however, the expected enzyme activity was not observed. The nitrogen of the pyridine moiety in **2i** may have repulsive interactions with the surrounding hydrophobic environment, resulting in a complete loss of the peroxygenase activity. Compared with phenylalanine, the inclusion or removal of one –CH_2_– leads to a significant reduction in catalytic activity. The catalytic activity is improved by introducing substituents such as –F (**2l**), –NO_2_ (**2m**), and –CH_3_ (**2n**) at the 4-position of the aromatic ring. Among five examined DFSMs, **2n** proved to have the best catalytic activity, with a TON of 641 and 85% *ee* of (*R*)-styrene oxide. In contrast, the less favorable larger *para*-substituents such as –CF_3_ (**2o**) and –OC(CH_3_)_3_ (**2p**) gave rise to a decrease in catalytic activity. The substitution at *ortho*- and *meta*-positions on the aromatic ring also affected the catalytic activity of P450BM3. Among three examined DFSMs (**2q**–**2s**), **2s** showed the highest catalytic activity with a TON of 1264 and 83% *ee* of (*R*)-styrene oxide, comparable with Im-C6-Phe. The catalytic activity of **2u** is lower than that of **2s**, probably because of the large, conjugated structure of naphthalene.

### 2.3. Effect of the DFSMs on the H_2_O_2_-Dependent Hydroxylation of P450BM3

The selectively direct hydroxylation of the C–H bond is one of the most fascinating catalytic functions for cytochrome P450 enzymes. The effect of the DFSMs with unnatural amino acids was next examined on the H_2_O_2_-dependent hydroxylation, catalyzed by a mutant of the P450BM3 heme domain. Three substrates, namely, ethylbenzene, hexane, and naphthalene, were chosen to represent three typical hydroxylation reactions, i.e., benzylic hydroxylation, alkane hydroxylation, and aromatic hydroxylation.

**Figure 3:**
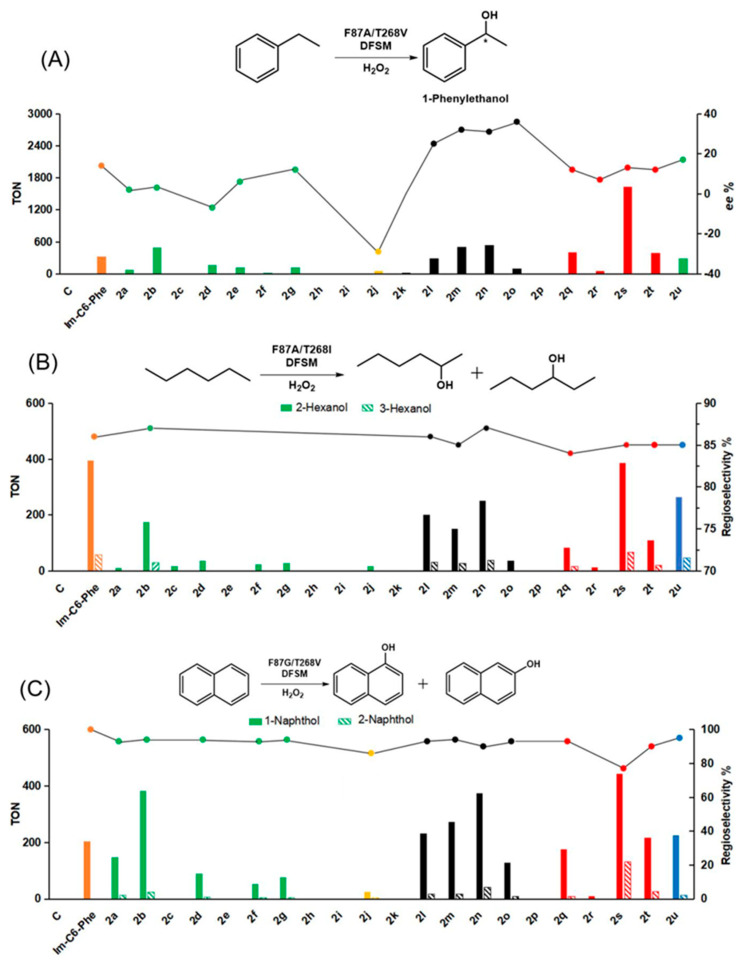
H_2_O_2_-dependent hydroxylations of ethylbenzene (**A**), hexane (**B**), and naphthalene (**C**) catalyzed by P450BM3 in the absence/presence of DFSMs. *ee*, enantiomeric excess (%) of (*R*)-(+)-1-phenylethanol determined by chiral GC and was calculated by ((*R*)-(+)-1-phenylethanol − (*S*)-(-)-1-phenylethanol)/((*R*)-(+)-1-phenylethanol + (*S*)-(-)-1-phenylethanol) according to the corresponding peak area. Regioselectivity was determined according to the corresponding TON, for hexane hydroxylation: 2-hexanol/(2-hexanol + 3-hexanol), and for naphthalene hydroxylation: 1-naphthol/(1-naphthol + 2-naphthol). The TON and *ee*% values are presented as bar and stacked line charts, respectively. *: The chiral carbon atom.

The hydroxylation of ethylbenzene catalyzed by the mutant F87A/T268V gave (*R*)-(+)-1-phenylethanol as the major product in the presence of the examined DFSMs (Figure 3A). No hydroxylated products were detected in the absence of the DFSMs. These results are consistent with those observed with Im-C6-Phe. Interestingly, the substituent effect of side chains in the DFSM is like the catalytic activity of benzylic hydroxylation with styrene epoxidation, namely, the DFSMs with good activity for epoxidation of styrene also showed high activity for hydroxylation of ethylbenzene. However, the beneficial DFSMs, including **2b**, **2l**–**2n**, **2q**, and **2s**–**2u**, showed comparable or significantly higher catalytic TONs than Im-C6-Phe. The highest TON of 1633 by the DFSM **2s** is more than 5-fold that of Im-C6-Phe (TON = 319 [34]). This result suggests that the introduction of suitable unnatural amino acids to DFSMs improved the catalytic efficiency of benzylic hydroxylation. Similarly, the *ee* of (*R*)-(+)-1-phenylethanol improved almost 2-fold by the DFSMs with substituted phenylalanine groups (**2l**–**2n**), indicating that additive hydrophobic interactions could also influence the enantioselectivity of benzylic hydroxylation. It is worth noting that the DFSM **2o** achieved the highest enantioselectivity of 36% *ee*, together with a lower TON of 104. This raises a new challenge on how to optimize the DFSM to improve activity and enantioselectivity at the same time.

Subsequently, the H_2_O_2_-dependent hydroxylation of hexane, a smaller substrate, was carried out by a combination of the mutant F87A/T268I and the DFSMs having unnatural amino acids to further understand the effect of tuning hydrophobic interactions (Figure 3B). Although the effect of side-chain substituents in the DFSMs on the catalytic activity of hexane hydroxylation showed almost the same trend as that observed for ethylbenzene hydroxylation and styrene epoxidation, the catalytic TONs by the beneficial DFSMs were obviously lower than that by Im-C6-Phe [38]. For instance, the best DFSM, **2s**, only showed a catalytic TON of 387 for the formation of 2-hexanol, which is approximately half of that by Im-C6-Phe. In addition, all the reactions gave approximately 85% regioselectivity for 2-hexanol, which is almost the same as that observed with Im-C6-Phe. This suggests that the anchoring group only influences the catalytic activity, rather than changing the orientation of the hexane in the active site, which is likely due to the small volume and linear shape of the substrate.

Finally, the hydroxylation of naphthalene was studied using a combination of the mutant F87G/T268V and the DFSMs having unnatural amino acids (Figure 3C). The DFSM **2b** showed good performance in both catalytic activity and regioselectivity for yielding 1-naphthol. Phenylalanine analogue-based DFSMs (**2i**–**2k**) still exhibit extremely low catalytic activity, whereas the *para*-substituted DFSMs **2l**–**2n** appeared to have much better catalytic activity and regioselectivity for yielding 1-naphthol. Compared with the other DFSMs, **2s** showed the highest catalytic activity with a TON of 444, which is more than 2-fold in comparison with that of Im-C6-Phe [40]. However, the regioselectivity of **2s** towards 1-naphthol is only 77%, indicating that the orientation of naphthalene has been altered in the active site.

### 2.4. Molecular Docking Simulation

**Figure 4:**
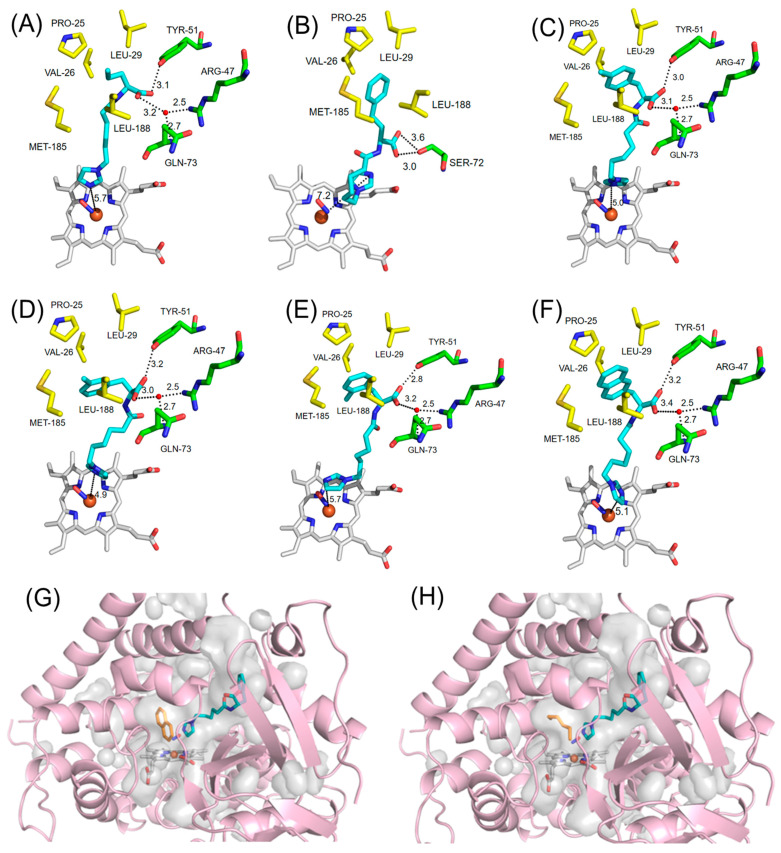
Docking simulation of DFSMs and substrates. (**A**) **2a**; (**B**) **2k**; (**C**) **2n**; (**D**) **2s**; (**E**) **2t**; (**F**) **2u**; (**G**) naphthalene; (**H**) hexane. The DFSMs, substrates, heme, hydrophobic binding pocket, and hydrophilic residues are shown as stick models colored in cyan, orange, white, yellow, and green, respectively. The iron atom and water molecule are shown as sphere models in orange and red, respectively. The cavities and pockets of the enzyme identified by Pymol are shown as surface models colored in light grey.

To understand the distinct catalytic activity of DFSMs, we performed molecular docking using AutoDock Vina [51]. As shown in Figure 2, Figure 3 and Figure 4 and Table 1, the catalytic activity of DFSMs is closely related to the binding affinity and the distance between the heme iron atom and the terminal nitrogen atom of the imidazolyl group (N–Fe), which is determined by the size and hydrophobicity of the anchoring group. In general, a higher binding affinity of DFSM and shorter N–Fe distance will be favorable for the enzyme activity enhancement. For example, **2s** has the best catalytic activity among all DFSMs in either epoxidation or hydroxylation reactions, owing to its highest binding affinity (−7.7 kcal/mol) and shortest N–Fe distance (4.9 Å). Moreover, the aromatic unnatural amino acid derivatives (**2i**–**2u**) showed superior trends in catalytic efficiency when compared with the alkyl (**2a**–**2f**) and cycloalkyl derivatives (**2g**, **2h**). Nonetheless, the DFSMs with longer alkyl chains (**2c**–**2f**) and bulky substituents on the aromatic ring (**2o**, **2p**, **2u**) lead to a reduction in activity, probably due to the steric hindrance effects, as decreasing binding energy and increasing N–Fe distance have been identified. In addition, the activity of specific DFSMs, such as **2k**, is almost completely lost as the binding mode of DFSM has changed drastically, resulting in a much larger N–Fe distance (7.2 Å, Figure 3B). Interestingly, the position of the substituent on the aromatic ring of DFSM also affects the catalytic ability. Substitution of the methyl group at the *meta*-position (**2s**) exhibits much higher activity in comparison with the *ortho*-substitution (**2t**). Similarly, the substitution of the nitro group at the *ortho*-position (**2r**) shows extremely low activity in hydroxyl reactions compared with the *meta*- (**2q**) and *para*- (**2m**) substituted isomers. It is worth noting that the different locations of the substitution might modulate the binding pattern of DFSMs and influence the N–Fe distances, which is supposed to be the reason for different catalytic efficiencies. Besides, the docking simulation results also show that while the DFSM occupies part of the substrate-binding tunnel, there is still enough space for the substrate to approach the active site (Figure 4G,H).

### 2.5. Dissociation Constant Measurement of DFSMs

**Figure 5:**
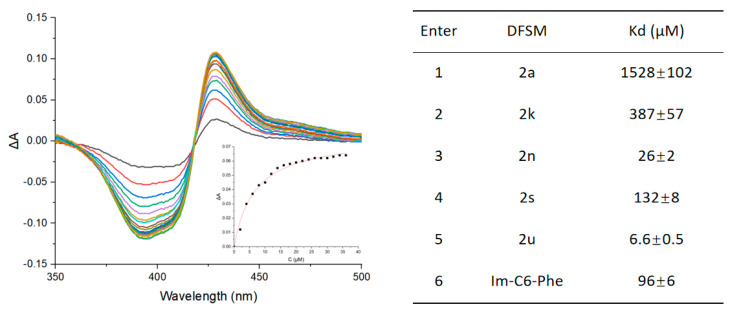
The UV-vis spectral change of F87A upon the addition of 2u (left), and the dissociation constants estimated by the titration experiment (inset). The dissociation constants of six representative DFSMs are summarized in the right table. The difference spectra were colored differently for clarity.

To further verify the reliability of the docking simulation results, we measured the dissociation constants of six representative DFSMs (Figure 5 and Figures S34–S38). The result showed a trend that the DFSMs with higher catalytic ability (**2n**, **2s**, **2u**, and Im-C6-Phe) have relatively smaller dissociation constants compared with the DFSMs with poor catalytic efficiency (**2a** and **2k**), which was consistent with docking simulation results. Thus, the size and hydrophobicity of the anchoring group indeed affected the binding of DFSMs. Moreover, based on the dissociation constants of the representative DFSMs, we believe that the DFSM concentration (500 μM) used for the reactions was reasonable. Therefore, 500 μM DFSMs could ensure that the activity of some loosely binding DFSMs would not be compensated by the high DFSM concentration. Meanwhile, it was enough to distinguish the catalytic performance among DFSMs under this concentration.

## 3. Materials and Methods

All chemicals were purchased from commercial sources (e.g., Aldrich, TCI, Alladin, and Adamas) and used without further purification. The DFSMs were prepared as described previously in the supplement materials (Schemes S1 and S2). All the mutants were from laboratory stock that was reported previously. The characterization spectra of all the synthesized compounds were listed in the supplementary materials (Figures S1–S33). 

### 3.1. General Procedure for the Synthesis of DFSMs (2s as an Example)

A DMF solution (20 mL) containing DIEA (426 mg, 2 mmol), DEPBT (546 mg, 1.1 mmol), and 6-(1H-imidazol-1-yl) hexanoic acid (300 mg, 1 mmol) was stirred at 0 °C for 1 h. (*S*)-3-Methy-l-phenylalanine methyl ester hydrochloride (415 mg, 1.1 mmol) was added to the reaction mixture. After 40 h, the reaction mixture was partitioned between DCM and H_2_O. The organic layer was washed with saturated NaCl aqueous solution and dried over MgSO_4_. The solution was concentrated under reduced pressure. The crude product was purified by column chromatography (Ethyl acetate/Methanol = 10:1) to afford mid-produce. A mixture of the mid-produce (150 mg, 0.5 mmol) in 0.6 mL sodium hydroxide aqueous solution (1 M) and 2 mL THF was stirred 1 h. The solution was acidified to pH = 5~6 with HCl (1 M). Next, the mixture was removed under reduced pressure and the residue was dissolved in ethanol. NaCl was separated by centrifugal. The ethanol layer was concentrated to give the product. The compounds were characterized by ^1^H-NMR, ^13^C-NMR, and LCMS.

Characterization of 2s: Colorless solid (86% yield), ^1^H-NMR (600 MHz, DMSO-d_6_) δ 9.15 (s, 1H), 8.18–8.16 (d, *J* = 12 Hz, 1H), 7.76 (s, 1H), 7.67 (s, 1H), 7.15–7.13 (t, *J* = 18 Hz, 1H), 7.04–7.00 (m, 3H), 4.42–4.38 (m, 1H), 4.14–4.11 (t, *J* = 18 Hz, 2H), 3.02–2.99 (m, 1H), 2.82–2.78 (m, 1H), 2.25 (s, 3H), 2.08–2.05 (m, 2H), 1.76–1.73 (m, 2H), 1.46–1.43 (m, 2H), 1.14–1.08 (m, 2H). ^13^C-NMR (151 MHz, DMSO-d_6_) δ173.64, 172.43, 138.14, 137.52, 135.56, 130.22, 128.47, 127.46, 126.60, 122.34, 120.39, 53.81, 48.66, 37.13, 35.12, 29.59, 25.35, 24.84, 21.49. LCMS (ESI) m/z [M + H]^+^: calcd. for C_22_H_26_N_3_O_3_: 344.1973; found: 344.1969.

### 3.2. General Procedure for Epoxidation of Styrene

P450BM3 variants (0.5 μM) were transferred to a glass sample bottle containing 0.1 M, pH 8.0 phosphate buffer, styrene (4 mM, dissolved in methanol), without or with DFSM (500 μM, dissolved in pH 8.0 phosphate buffer). The reaction was initiated by the addition of H_2_O_2_ (20 mM, dissolved in pH 8.0 phosphate buffer). The reaction mixture was incubated in a water bath at 25 °C for 30 min. The total volume of the reaction was 1 mL, and the final concentration of the methanol was 10%. The reaction mixture was quenched and extracted with 1 mL of ethyl acetate, and the organic phase was separated and dried with sodium sulphate anhydrous. The product was identified according to the retention time of authentic samples by gas chromatography (GC). The product was analyzed by GC by benzophenone as an internal standard.

### 3.3. General Procedure for Hydroxylation of Ethylbenzene

P450BM3 variants (0.5 μM) were transferred to a glass sample bottle containing 0.1 M, pH 8.0 phosphate buffer, ethylbenzene (10 mM, dissolved in dimethyl sulfoxide), without or with DFSM (500 μM, dissolved in pH 8.0 phosphate buffer). The reaction was initiated by the addition of H_2_O_2_ (60 mM, dissolved in pH 8.0 phosphate buffer). The reaction mixture was incubated in a water bath at 25 °C for 30 min. The total volume of the reaction was 1 mL, and the final concentration of the dimethyl sulfoxide was 2%. The reaction mixture was quenched and extracted with 1 mL of ethyl acetate, and the organic phase was separated and dried with sodium sulphate anhydrous. The product was identified according to the retention time of authentic samples by GC. The product was analyzed by GC by benzophenone as an internal standard.

### 3.4. General Procedure for Hydroxylation of n-Hexane

P450BM3 variants (0.5 µM) were transferred to a glass sample bottle containing 0.1 M, pH 8.0 phosphate buffer, n-hexane (4 mM, dissolved in methanol), without or with DFSM (500 µM, dissolved in pH 8.0 phosphate buffer). The reaction was initiated by the addition of H_2_O_2_ (20 mM, dissolved in pH 8.0 phosphate buffer). The reaction mixture was incubated in a water bath at 25 °C for 30 min. The total volume of the reaction was 1 mL, and the final concentration of the methanol was 10%. The mixture was quenched and extracted with 1 mL of ethyl acetate, and the organic phase was separated and dried with sodium sulphate anhydrous. The product was identified according to the retention time of authentic samples by GC. The product was analyzed by GC by 3-pentanol as an internal standard.

### 3.5. General Procedure for Hydroxylation of Naphthalene

P450BM3 variants (0.5 μM) were transferred to a glass sample bottle containing 0.1 M, pH 8.0 phosphate buffer, naphthalene (1 mM, dissolved in dimethyl sulfoxide), without or with DFSM (500 μM, dissolved in pH 8.0 phosphate buffer). The reaction was initiated by the addition of H_2_O_2_ (20 mM, dissolved in pH 8.0 phosphate buffer). The reaction mixture was incubated in a water bath at 25 °C for 30 min. The total volume of the reaction was 1 mL, and the final concentration of the dimethyl sulfoxide was 2%. The reaction was stopped by the addition of dilute aqueous HCl. The reaction mixture was neutralized and extracted with 1 mL of ethyl acetate, and the organic phase was separated and dried with anhydrous sodium sulphate. The product was identified according to the retention time of authentic samples by gas chromatography (GC). The product was analyzed by GC and by benzophenone as an internal standard.

### 3.6. Product Analysis

#### 3.6.1. Gas Chromatography (GC)

The products of styrene epoxidation, ethylbenzene and naphthalene hydroxylation were respectively performed on a Shimadzu GC-2010 plus gas chromatograph with a flame ionization detector (FID) and fitted with an AOC-20i auto sampler system by using a DB-5 column (length: 30 m, internal diameter: 0.25 mm, film thickness: 1.0 µm, Agilent, China).

The product of n-hexane hydroxylation was performed on a Shimadzu GC-2010 plus gas chromatograph with a flame ionization detector (FID) and fitted with an AOC-20i auto sampler system by using a HP-INNOWAX column (length: 30 m, internal diameter: 0.25 mm, film thickness: 0.5 µm, Agilent, China).

#### 3.6.2. Chiral Gas Chromatography

The product of styrene epoxidation chiral analysis was performed on a Shimadzu GC-2030 plus gas chromatograph equipped with a Astec CHIRALDEX G-TA column (length: 30 m, internal diameter: 0.25 mm, film thickness: 0.12 µm, Agilent, China), a flame ionization detector, and an AOC/20i plus auto sampler system.

The product of ethylbenzene hydroxylation chiral analysis was performed on a Shimadzu GC-2030 plus gas chromatograph equipped with a CP-Chirasil Dex CB column (length: 25 m, internal diameter: 0.25 mm, film thickness: 0.25 µm, Agilent, China), a flame ionization detector, and an AOC/20i plus auto sampler system.

### 3.7. Docking Simulation of the DFSMs

Docking simulation of DFSMs (**2a**–**2u**) or substrates (naphthalene and hexane) to the substrate binding site of P450 BM3 was performed with AutoDock Vina [51]. The protein crystal structure of the P450 BM3 F87A mutant complexed with Im-C6-Phe and hydroxylamine (PDB entry: 7EGN) was chosen as the rigid acceptor for the docking simulation of the substrates, while the Im-C6-Phe molecule was removed prior to the docking simulations of different DFSMs (**2a**–**2u**). The twenty lowest energy docking conformations were generated for each DFSM, and the docking conformation most closely resembling the Im-C6-Phe bound structure was selected. Protein structure graphics were created by PyMOL (https://www.schrodinger.com).

### 3.8. Determination of Dissociation Constants of DFSMs

The dissociation constants of DFSMs were determined by UV-vis titration experiment through monitoring the change of Soret band. The concentration of the enzyme is 4 μM. The concentration of the mother liquid for titration was 50 mM (**2a** and **2k**), 2.5 mM (**2n**, **2s**, and Im-C6-Phe), or 1 mM (**2u**). Spectral change at Soret band was plotted against concentration of DFSMs. The dissociation constant was calculated based on the hyperbolic equation fitted curve.

## 4. Conclusions

We have successfully exploited unnatural amino acids to synthesize a series of new DFSMs and evaluated their applications in the P450–H_2_O_2_ catalytic system. The catalytic activity and selectivity are well modulated by the size and length of side chains on the unnatural amino acids; for instance, the catalytic TON of ethylbenzene hydroxylation was improved 5-fold by DFSM **2s** compared with Im-C6-Phe, which can be ascribed to the changes of the binding affinity and orientation of DFSMs in the active site, as suggested by the docking model. This work well enriched the DFSM toolbox for activating the peroxide-shunt pathway of P450BM3 and might provide customized DFSM solutions for specific substrates and reactions. With the expansion of the DFSM library, we consider that the system can be further upgraded by rationally designing the structure of DFSMs. In addition, we believe that the catalytic activity and substrate scope can be further improved through a combination of protein engineering and DFSM optimization, which is currently underway in our laboratory.

## Figures and Tables

**Table 1:** Docking simulation of DFSMs.

DFSM	Score (kcal/mol)	L (Fe-N) (Å)	DFSM	Score (kcal/mol)	L (Fe-N) (Å)
**2a**	−5.9	5.7	**2l**	−7.2	5.6
**2b**	−6.6	5.1	**2m**	−7.1	5.0
**2c**	−6.1	5.6	**2n**	−7.3	5.0
**2d**	−6.4	5.6	**2o**	−6.6	5.6
**2e**	−6.8	5.9	**2p**	−6.3	5.5
**2f**	−6.4	5.6	**2q**	−7.4	5.1
**2g**	−6.9	5.6	**2r**	−6.9	5.7
**2h**	−6.9	5.8	**2s**	−7.7	4.9
**2i**	−6.5	6.2	**2t**	−7.0	5.7
**2j**	−6.7	5.6	**2u**	−7.4	5.1
**2k**	−6.9	7.2			

## Data Availability

Not applicable.

## Supplementary Materials

The following supporting information can be downloaded at: https://www.mdpi.com/article/10.3390/ijms23147901/s1.

## References

[1] Valikhani D., Bolivar J.M., Pelletier J.N. (2021). An overview of cytochrome P450 immobilization strategies for drug metabolism studies, biosensing, and biocatalytic applications: Challenges and opportunities. ACS Catal..

[2] Newmister S.A., Srivastava K.R., Espinoza R.V., Haatveit K.C., Khatri Y., Martini R.M., Garcia-Borràs M., Podust L.M., Houk K.N., Sherman D.H. (2020). Molecular basis of iterative C─H oxidation by TamI, a multifunctional P450 monooxygenase from the tirandamycin biosynthetic pathway. ACS Catal..

[3] Behrendorff J.B.Y.H., Gillam E.M.J. (2017). Prospects for Applying Synthetic Biology to Toxicology: Future Opportunities and Current Limitations for the Repurposing of Cytochrome P450 Systems. Chem. Res. Toxicol..

[4] Wu Z., Lei T., Shen C., Wang Z., Cao D., Hou T. (2019). ADMET Evaluation in Drug Discovery. 19. Reliable Prediction of Human Cytochrome P450 Inhibition Using Artificial Intelligence Approaches. J. Chem. Inf. Model..

[5] Steck V., Kolev J.N., Ren X., Fasan R. (2020). Mechanism-Guided Design and Discovery of Efficient Cytochrome P450-Derived C-H Amination Biocatalysts. J. Am. Chem. Soc..

[6] Arya C.K., Yadav S., Fine J., Casanal A., Chopra G., Ramanathan G., Vinothkumar K.R., Subramanian R. (2020). A 2-Tyr-1-carboxylate Mononuclear Iron Center Forms the Active Site of a *Paracoccus* Dimethylformamidase. Angew. Chem. Int. Ed..

[7] Mukherjee G., Satpathy J.K., Bagha U.K., Mubarak M.Q.E., Sastri C.V., de Visser S.P. (2021). Inspiration from Nature: Influence of Engineered Ligand Scaffolds and Auxiliary Factors on the Reactivity of Biomimetic Oxidants. ACS Catal..

[8] Peng Y., Gao C., Zhang Z., Wu S., Zhao J., Li A. (2022). A Chemoenzymatic Strategy for the Synthesis of Steroid Drugs Enabled by P450 Monooxygenase-Mediated Steroidal Core Modification. ACS Catal..

[9] Chen H., Huang M., Yan W., Bai W.-J., Wang X. (2021). Enzymatic Regio- and Enantioselective C–H Oxyfunctionalization of Fatty Acids. ACS Catal..

[10] Espinoza R.V., Haatveit K.C., Grossman S.W., Tan J.Y., McGlade C.A., Khatri Y., Newmister S.A., Schmidt J.J., Garcia-Borràs M., Montgomery J. (2021). Engineering P450 TamI as an Iterative Biocatalyst for Selective Late-Stage C-H Functionalization and Epoxidation of Tirandamycin Antibiotics. ACS Catal..

[11] Münch J., Püllmann P., Zhang W., Weissenborn M.J. (2021). Enzymatic Hydroxylations of Sp3-Carbons. ACS Catal..

[12] Li F., Renata H. (2021). A Chiral-Pool-Based Strategy to Access Trans-Syn-Fused Drimane Meroterpenoids: Chemoenzymatic Total Syntheses of Polysin, N-Acetyl-Polyveoline and the Chrodrimanins. J. Am. Chem. Soc..

[13] Miller D.C., Lal R.G., Marchetti L.A., Arnold F.H. (2022). Biocatalytic One-Carbon Ring Expansion of Aziridines to Azetidines via a Highly Enantioselective [1,2]-Stevens Rearrangement. J. Am. Chem. Soc..

[14] Miller D.C., Athavale S.V., Arnold F.H. (2022). Combining Chemistry and Protein Engineering for New-to-Nature Biocatalysis. Nat. Synth..

[15] Nam D., Tinoco A., Shen Z., Adukure R.D., Sreenilayam G., Khare S.D., Fasan R. (2022). Enantioselective Synthesis of α-Trifluoromethyl Amines via Biocatalytic N-H Bond Insertion with Acceptor-Acceptor Carbene Donors. J. Am. Chem. Soc..

[16] Karasawa M., Yonemura K., Stanfield J.K., Suzuki K., Shoji O. (2022). Ein Designeraußenmembranprotein Fördert Die Aufnahme von Täuschmolekülen in Einen Auf Zytochrom P450BM3 Beruhenden Ganzzellbiokatalysator. Angew. Chem. Int. Ed..

[17] Yang Y., Cho I., Qi X., Liu P., Arnold F.H. (2019). An Enzymatic Platform for the Asymmetric Amination of Primary, Secondary and Tertiary C(Sp3)-H Bonds. Nat. Chem..

[18] Jiang Y., Peng W., Li Z., You C., Zhao Y., Tang D., Wang B., Li S. (2021). Unexpected Reactions of α,β-Unsaturated Fatty Acids Provide Insight into the Mechanisms of CYP152 Peroxygenases. Angew. Chem. Int. Ed..

[19] Knorrscheidt A., Soler J., Hünecke N., Püllmann P., Garcia-Borràs M., Weissenborn M.J. (2021). Accessing Chemo- and Regioselective Benzylic and Aromatic Oxidations by Protein Engineering of an Unspecific Peroxygenase. ACS Catal..

[20] Pickl M., Kurakin S., Cantú Reinhard F.G., Schmid P., Pöcheim A., Winkler C.K., Kroutil W., de Visser S.P., Faber K. (2019). Mechanistic Studies of Fatty Acid Activation by CYP152 Peroxygenases Reveal Unexpected Desaturase Activity. ACS Catal..

[21] Li H., Liu Y. (2020). Mechanistic Investigation of Isonitrile Formation Catalyzed by the Nonheme Iron/α-KG-Dependent Decarboxylase (ScoE). ACS Catal..

[22] Wang S., Jiang S., Chen H., Bai W.J., Wang X. (2020). Directed Evolution of a Hydroxylase into a Decarboxylase for Synthesis of 1-Alkenes from Fatty Acids. ACS Catal..

[23] Olmedo A., Aranda C., del Río J.C., Kiebist J., Scheibner K., Martínez A.T., Gutiérrez A. (2016). From Alkanes to Carboxylic Acids: Terminal Oxygenation by a Fungal Peroxygenase. Angew. Chem. Int. Ed..

[24] Wang Y., Lan D., Durrani R., Hollmann F. (2017). Peroxygenases En Route to Becoming Dream Catalysts. What Are the Opportunities and Challenges?. Curr. Opin. Chem. Biol..

[25] Xu G., Michele C., Thangavelu S., Kim M.K., Gerrit J.P. (2020). Enantiocomplementary Epoxidation Reactions Catalyzed by an Engineered Cofactor-Independent Non-natural Peroxygenase. Angew. Chem. Int. Ed..

[26] Sigmund M.-C., Poelarends G.J. (2020). Current State and Future Perspectives of Engineered and Artificial Peroxygenases for the Oxyfunctionalization of Organic Molecules. Nat. Catal..

[27] Gomez de Santos P., Lazaro S., Viña-Gonzalez J., Hoang M.D., Sánchez-Moreno I., Glieder A., Hollmann F., Alcalde M. (2020). Evolved Peroxygenase–Aryl Alcohol Oxidase Fusions for Self-Sufficient Oxyfunctionalization Reactions. ACS Catal..

[28] Grogan G. (2021). Hemoprotein Catalyzed Oxygenations: P450s, UPOs, and Progress toward Scalable Reactions. JACS Au.

[29] Shoji O., Watanabe Y. (2014). Peroxygenase Reactions Catalyzed by Cytochromes P450. J. Biol. Inorg. Chem..

[30] Yu D., Wang J.-B., Reetz M.T. (2019). Exploiting Designed Oxidase-Peroxygenase Mutual Benefit System for Asymmetric Cascade Reactions. J. Am. Chem. Soc..

[31] Coleman T., Kirk A.M., Lee J.H.Z., Doherty D.Z., Bruning J.B., Krenske E.H., De Voss J.J., Bell S.G. (2022). Different Geometric Requirements for Cytochrome P450-Catalyzed Aliphatic versus Aromatic Hydroxylation Results in Chemoselective Oxidation. ACS Catal..

[32] Guengerich F.P. (2018). Mechanisms of Cytochrome P450-Catalyzed Oxidations. ACS Catal..

[33] Whitehouse C.J.C., Bell S.G., Wong L.-L. (2012). P450(BM3) (CYP102A1): Connecting the Dots. Chem. Soc. Rev..

[34] Ma N., Chen Z., Chen J., Chen J., Wang C., Zhou H., Yao L., Shoji O., Watanabe Y., Cong Z. (2018). Dual-Functional Small Molecules for Generating an Efficient Cytochrome P450BM3 Peroxygenase. Angew. Chem. Int. Ed..

[35] Xu J., Wang C., Cong Z. (2019). Strategies for Substrate-regulated P450 Catalysis: From Substrate Engineering to Co-catalysis. Chem. Eur. J..

[36] Di S., Fan S., Jiang F., Cong Z. (2022). A Unique P450 Peroxygenase System Facilitated by a Dual-Functional Small Molecule: Concept, Application, and Perspective. Antioxidants.

[37] Zhao P., Chen J., Ma N., Chen J., Qin X., Liu C., Yao F., Yao L., Jin L., Cong Z. (2021). Enabling Highly (R)-Enantioselective Epoxidation of Styrene by Engineering Unique Non-Natural P450 Peroxygenases. Chem. Sci..

[38] Chen J., Kong F., Ma N., Zhao P., Liu C., Wang X., Cong Z. (2019). Peroxide-Driven Hydroxylation of Small Alkanes Catalyzed by an Artificial P450BM3 Peroxygenase System. ACS Catal..

[39] Jiang Y., Wang C., Ma N., Chen J., Liu C., Wang F., Xu J., Cong Z. (2020). Regioselective Aromatic O-Demethylation with an Artificial P450BM3 Peroxygenase System. Catal. Sci. Technol..

[40] Chen Z., Chen J., Ma N., Zhou H., Cong Z. (2018). Selective Hydroxylation of Naphthalene Using the H2O2-Dependent Engineered P450BM3 Driven by Dual-Functional Small Molecules. J. Porphyr. Phthalocyanines.

[41] Ma N., Fang W., Liu C., Qin X., Wang X., Jin L., Wang B., Cong Z. (2021). Switching an Artificial P450 Peroxygenase into Peroxidase via Mechanism-Guided Protein Engineering. ACS Catal..

[42] Zhang X., Jiang Y., Chen Q., Dong S., Feng Y., Cong Z., Shaik S., Wang B. (2021). H-Bonding Networks Dictate the Molecular Mechanism of H2O2 Activation by P450. ACS Catal..

[43] Cong Z., Shoji O., Kasai C., Kawakami N., Sugimoto H., Shiro Y., Watanabe Y. (2015). Activation of Wild-Type Cytochrome P450BM3 by the next Generation of Decoy Molecules: Enhanced Hydroxylation of Gaseous Alkanes and Crystallographic Evidence. ACS Catal..

[44] Yonemura K., Ariyasu S., Stanfield J.K., Suzuki K., Onoda H., Kasai C., Sugimoto H., Aiba Y., Watanabe Y., Shoji O. (2020). Systematic Evolution of Decoy Molecules for the Highly Efficient Hydroxylation of Benzene and Small Alkanes Catalyzed by Wild-Type Cytochrome P450BM3. ACS Catal..

[45] Karasawa M., Stanfield J.K., Yanagisawa S., Shoji O., Watanabe Y. (2018). Whole-Cell Biotransformation of Benzene to Phenol Catalysed by Intracellular Cytochrome P450BM3 Activated by External Additives. Angew. Chem. Int. Ed..

[46] Willot S.J.P., Tieves F., Girhard M., Urlacher V.B., Hollmann F., de Gonzalo G. (2019). P450BM3-Catalyzed Oxidations Employing Dual Functional Small Molecules. Catalysts..

[47] Lovell S., Zhang L., Kryza T., Neodo A., Bock N., De Vita E., Williams E.D., Engelsberger E., Xu C., Bakker A.T. (2021). A Suite of Activity-Based Probes to Dissect the KLK Activome in Drug-Resistant Prostate Cancer. J. Am. Chem. Soc..

[48] Adaligil E., Song A., Cunningham C.N., Fairbrother W.J. (2021). Ribosomal Synthesis of Macrocyclic Peptides with Linear γ4- and β-Hydroxy-γ4-Amino Acids. ACS Chem. Biol..

[49] Faraggi T.M., Rouget-Virbel C., Rincón J.A., Barberis M., Mateos C., García-Cerrada S., Agejas J., de Frutos O., MacMillan D.W.C. (2021). Synthesis of Enantiopure Unnatural Amino Acids by Metallaphotoredox Catalysis. Org. Process Res. Dev..

[50] Hu L., Xu S., Zhao Z., Yang Y., Peng Z., Yang M., Wang C., Zhao J. (2016). Ynamides as Racemization-Free Coupling Reagents for Amide and Peptide Synthesis. J. Am. Chem. Soc..

[51] Oleg T., Olson A.J. (2010). AutoDock Vina: Improving the Speed and Accuracy of Docking with a New Scoring Function, Effificient Optimization, and Multithreading. J. Comput. Chem..

