# Supplementary material for: Co-Crystal Structure-Guided Optimization of Dual-Functional Small Molecules for Improving the Peroxygenase Activity of Cytochrome P450BM3
Source: Int J Mol Sci. 2022 Jul 18;23(14):7901. doi: 10.3390/ijms23147901 (PMC9317928; doi:10.3390/ijms23147901)
Supplement: Supplementary file 1 [file ijms-23-07901-s001.zip › ijms-1767720-supplementary.pdf]

Supporting Information:

Co-Crystal Structure-Guided Optimization of Dual-Functional Small Molecules for Improving the Peroxygenase Activity of Cytochrome P450BM3

Xiangquan Qin [1,2,†], Yiping Jiang [2,†], Jie Chen [2], Fuquan Yao [2], Panxia Zhao [2,3], Longyi Jin [1,*] and Zhiqi Cong [2,3,*]

1 Department of Chemistry, Yanbian University, Yanji 133002, China; qxiangquan@163.com
2 CAS Key Laboratory of Biofuels, Shandong Provincial Key Laboratory of Synthetic Biology,
Qingdao Institute of Bioenergy and Bioprocess Technology, Chinese Academy of Sciences, Qingdao 266101, China; jiangyp@qibebt.ac.cn (Y.J.); chen_jie@qibebt.ac.cn (J.C.); yaofq@qibebt.ac.cn (F.Y.); zhaopx@qibebt.ac.cn (P.Z.)
3 University of Chinese Academy of Sciences, Beijing 100049, China
* Correspondence: lyjin@ybu.edu.cn (L.J.); congzq@qibebt.ac.cn (Z.C.)
† These authors contributed equally to this work.

Product analysis

Gas Chromatography (GC)

The analytical condition for styrene epoxidation was as follows: splitting ratio: 1/9, temperature program: injector 280℃, detector 280℃, 100℃ oven for 1 min, then 15℃ /min gradient to 200℃, 60℃ /min gradient to 280℃ and then 280℃ for 8 min (total 17.0 min).

The analytical conditions for 3-hexanol, 2-hexanol and 1-hexanol were as follows: splitting ratio: 1/9, temperature program: injector 260℃, detector 260℃, 80℃ oven, then 10℃/min gradient to 160℃, 60℃/min gradient to 240℃ and then 240℃ for 2 min (total 11.33 min).

The analytical conditions for 1-phenylethanol and 2-phenylethanol were as follows: splitting ratio: 1/9, temperature program: injector 280℃, detector 280℃, 100℃ oven for 1 min, then 25℃ /min gradient to 180℃, 5℃ /min gradient to 220℃, 30℃ /min gradient to 280℃ and then 280℃ for 5 min (total 19.2 min).

The analytical condition for naphthalene hydroxylation was as follows: splitting ratio: 1/9, temperature program: injector 280℃, detector 300℃, 100℃ oven for 1 min, then 15℃ /min gradient to 200℃, 60℃ /min gradient to 280℃ and then 280℃ for 4 min (total 13.0 min).

Chiral Gas Chromatography

The analytical condition of styrene epoxidation was as follows: splitting ratio: 1/9, temperature program: injector 200℃, detector 200℃, 80℃ oven for 3 min, then 10℃/min gradient to 100℃ for 5 min, 5℃ /min gradient to 105℃ for 7 min, 60℃/min gradient to 170℃ for 3 min (total 19.08 min).

The analytical condition of ethylbenzene hydroxylation was as follows: splitting ratio: 1/9, temperature program: injector 250℃, detector 250℃, the initial temperature is 80℃, then 10℃/min gradient to 125℃ for 8 min, 60℃ /min gradient to 190℃ for 5 min (total 18.6 min).

synthesis

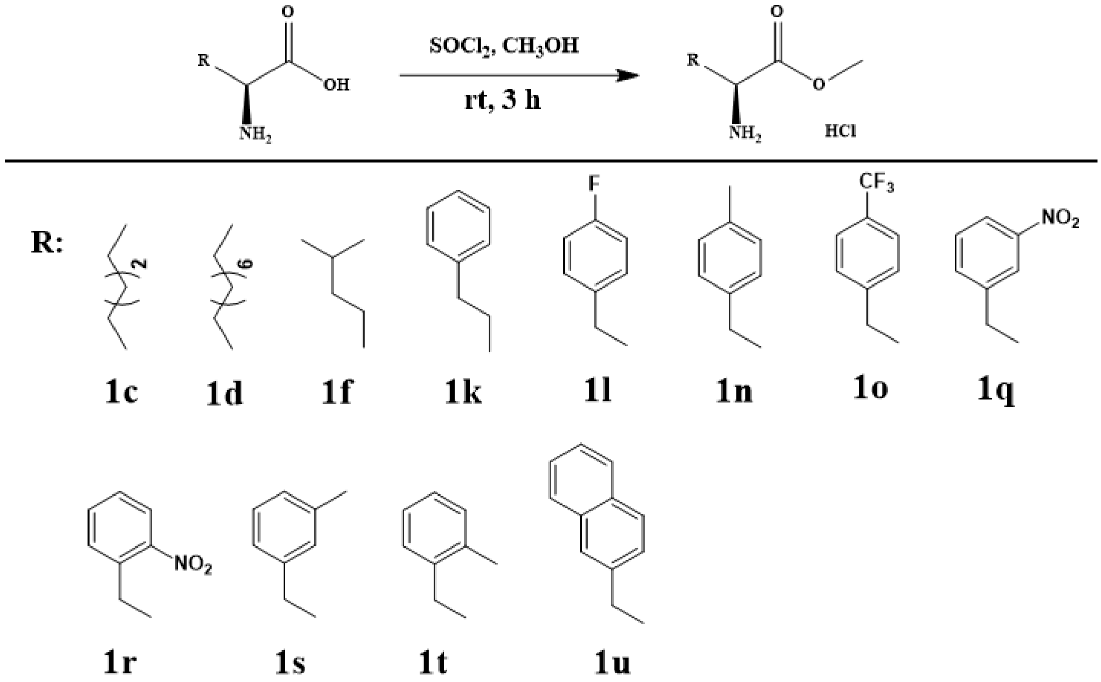

Scheme S1. Synthesis of amino acid hydrochloride.

Synthesis of **1c**: To a suspension of (S)-2-Aminooctanoic acid (1.0 g, 6.28 mmol) in MeOH (15mL) at 0 ℃ (ice bath) was added dropwise with stirring $SOCl_2$ (6.5 mL, 9.4 mmol) over 1 h. The flask was fitted with a reflux condenser and heated to 60 °C for 3 h then cooled to room temperature. After removed the solvent in a rotary evaporator, washed twice with ether to obtain produce colorless soild (95% yield), ^{1}H-NMR (600 MHz, DMSO-d_6) δ 8.71(s, 3H), 3.96-3.94 (t, *J* = 12 Hz, 1H), 3.74 (s, 3H), 1.82-1.78 (m, 2H), 1.29-1.25 (m, 8H), 0.87-0.85 (t, *J* = 12 Hz, 3H).

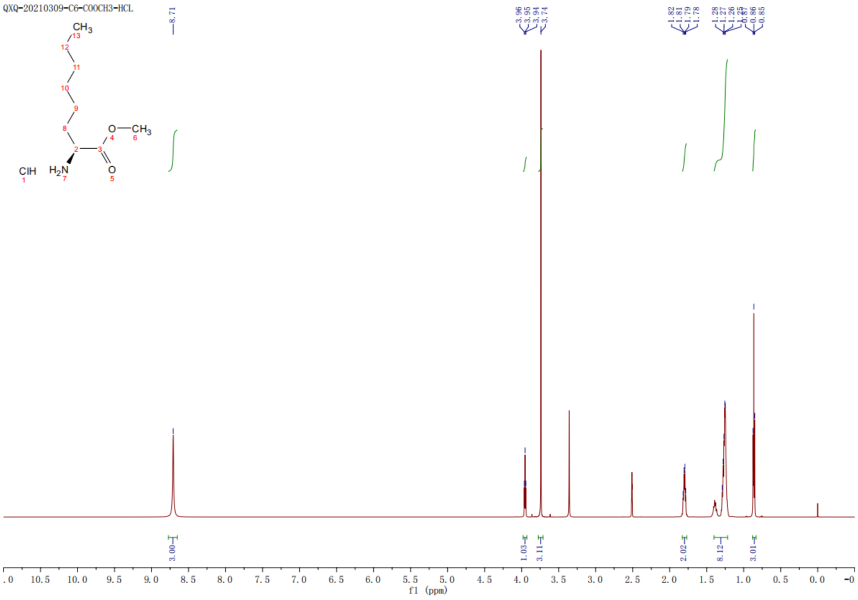

Figure S1. ^{1}H-NMR spectra of **1c**.

1d: Colorless soild (96% yield), ^{1}H-NMR (600 MHz, DMSO-d_6) δ 8.67 (s, 3H), 3.99-3.96 (t, J = 18 Hz, 1H), 3.74 (s, 3H), 1.80-1.77 (m, 2H), 1.39-1.36 (m, 1H), 1.30-1.25 (m, 1H), 0.87-0.85 (t, J = 12 Hz, 3H).

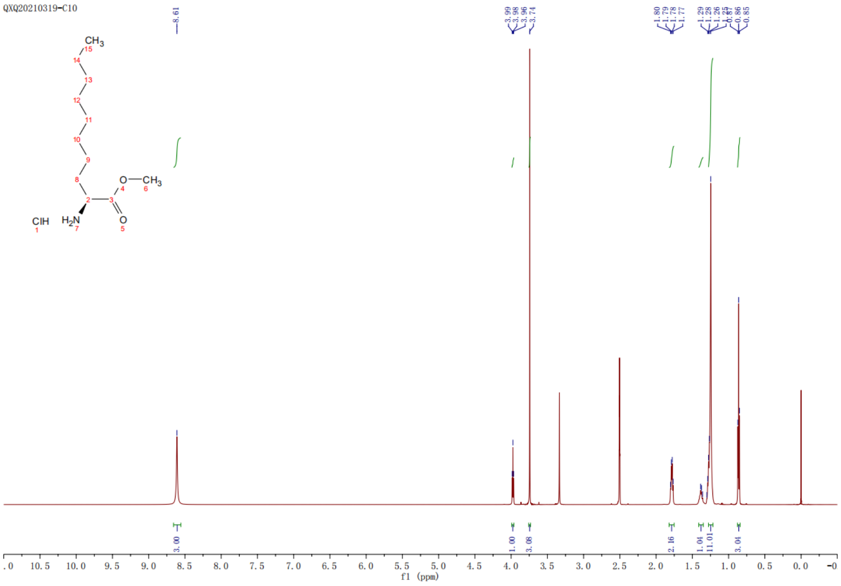

Figure S2. ^{1}H-NMR spectra of **1d**.

1e: Colorless soild (94% yield), ^{1}H-NMR (600 MHz, DMSO-d_6) δ 8.67 (s, 3H), 3.99-3.96 (t, J = 18 Hz, 1H), 3.75 (s, 1H), 1.83-1.79 (m, 2H), 1.53-1.49 (m, 1H), 1.32-1.25 (m, 1H), 1.18-1.12 (m, 1H), 0.87-0.85 (m, 6H).

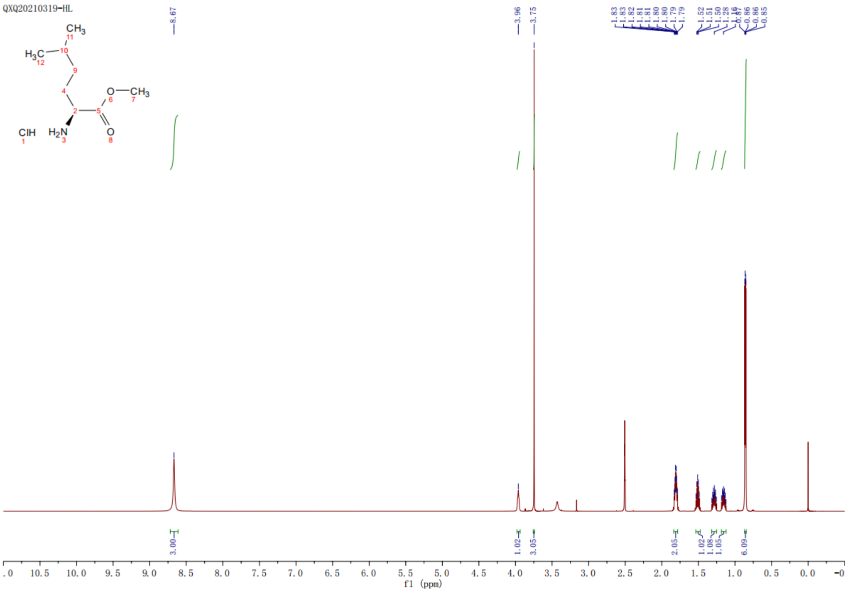

Figure S3. ^{1}H-NMR spectra of **1e**.

1k: Colorless soild (97% yield), ^{1}H-NMR (600 MHz, DMSO-d_6) δ 8.87 (s, 3H), 7.32-7.29 (m, 2H), 7.25-7.20 (m, 2H), 4.00-3.98 (t, *J* = 12 Hz, 1H), 3.73 (s, 3H), 2.81-2.76 (m, 1H), 2.67-2.62 (m, 1H), 2.15-2.11 (m, 2H).

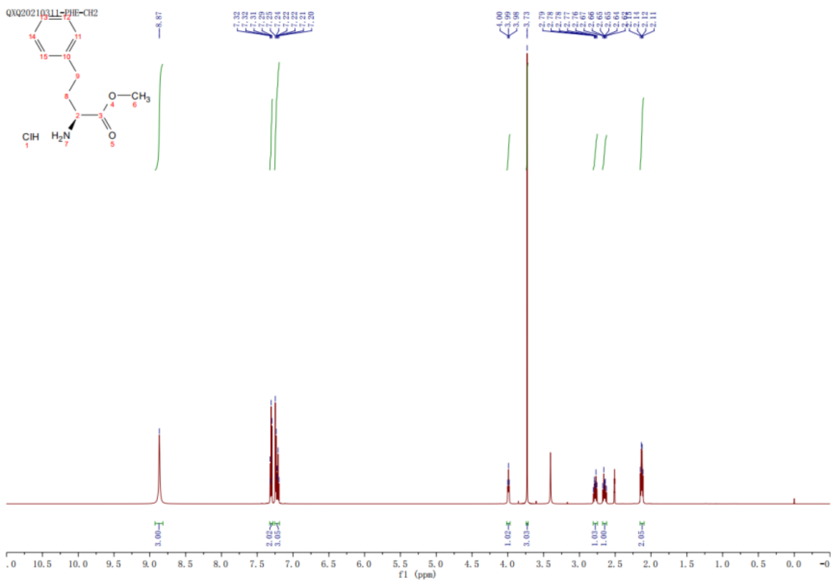

Figure S4. ^{1}H-NMR spectra of **1k**.

1l: Colorless soild (96% yield), ^{1}H-NMR (600 MHz, DMSO-d_6) δ 7.15 (s, 3H), 7.32-7.29 (m, 2H), 7.18-7.15 (t, *J* = 18 Hz, 2H), 4.25-4.23 (t, *J* = 12 Hz, 1H), 3.67 (s, 3H), 3.23-3.20 (m, 1H), 3.15-3.11 (m, 1H).

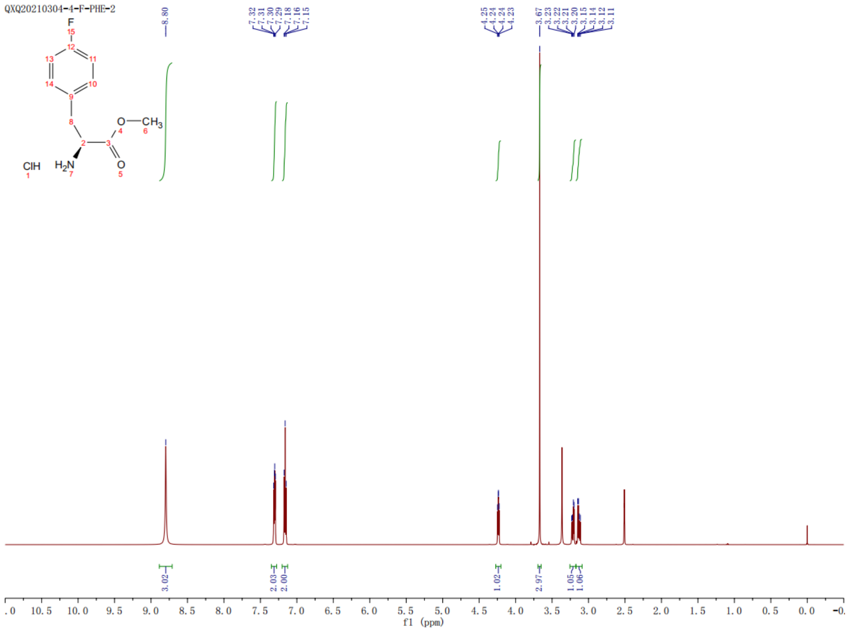

Figure S5. ^{1}H-NMR spectra of **1l**.

1n: Colorless soild (92% yield), ^{1}H-NMR (600 MHz, DMSO-d$_6$) δ 8.80 (s, 3H), 7.14-7.11 (m, 4H), 4.19-4.17 (t, *J* = 12 Hz, 1H), 3.65 (s, 3H), 3.20-3.17 (m, 1H), 3.09-3.06 (m, 1H), 2.28 (s, 3H).

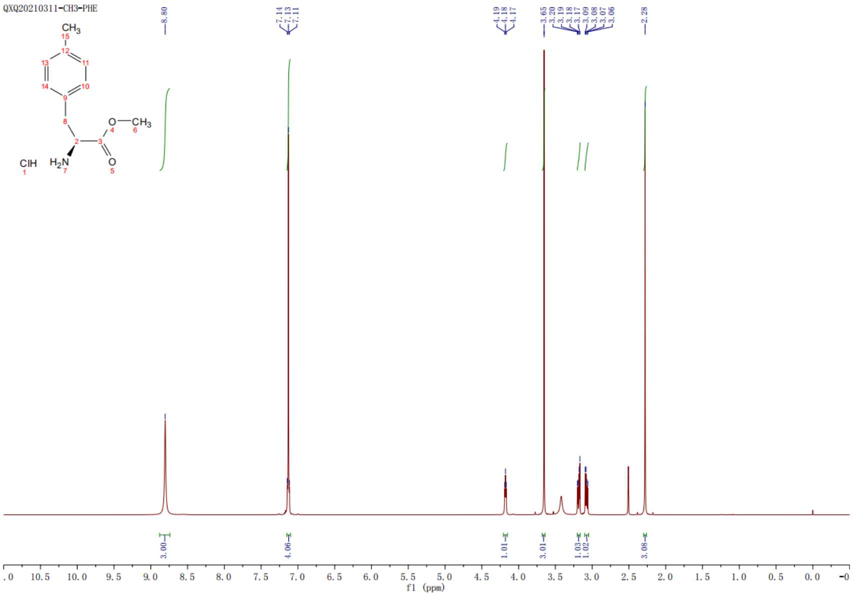

Figure S6. ^{1}H-NMR spectra of **1n.**

1o: Colorless soild (94% yield), ^{1}H-NMR (600 MHz, DMSO-d$_6$) δ 8.85 (s, 3H), 7.71-7.70 (d, *J* = 6 Hz, 2H), 7.52-7.51 (d, *J* = 6 Hz, 2H), 4.35-4.33 (t, *J* = 12 Hz, 1H), 3.69 (s, 3H), 3.34-3.31 (m, 1H), 3.27-3.23 (m, 1H).

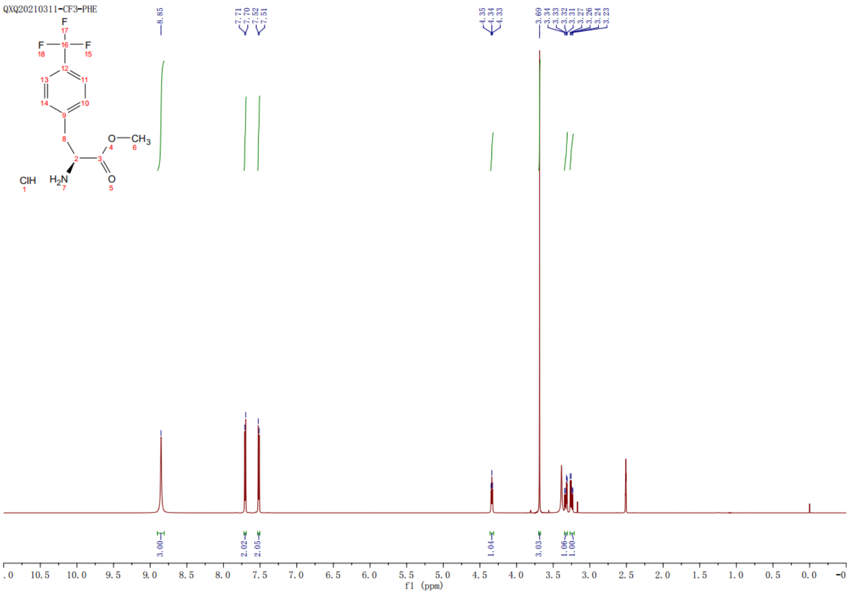

Figure S7. ^{1}H-NMR spectra of 1o.

1q: Light yellow soild (93% yield), ^{1}H-NMR (600 MHz, DMSO-d_6) δ 9.00 (s, 3H), 8.06-8.05 (m, 1H), 7.76-7.74 (m, 1H), 7.68-7.67 (m, 1H), 6.61-7.58 (m, 1H), 4.25-4.22 (t, *J* = 18 Hz, 1H), 3.58 (s, 3H), 3.52-3.39 (m, 2H).

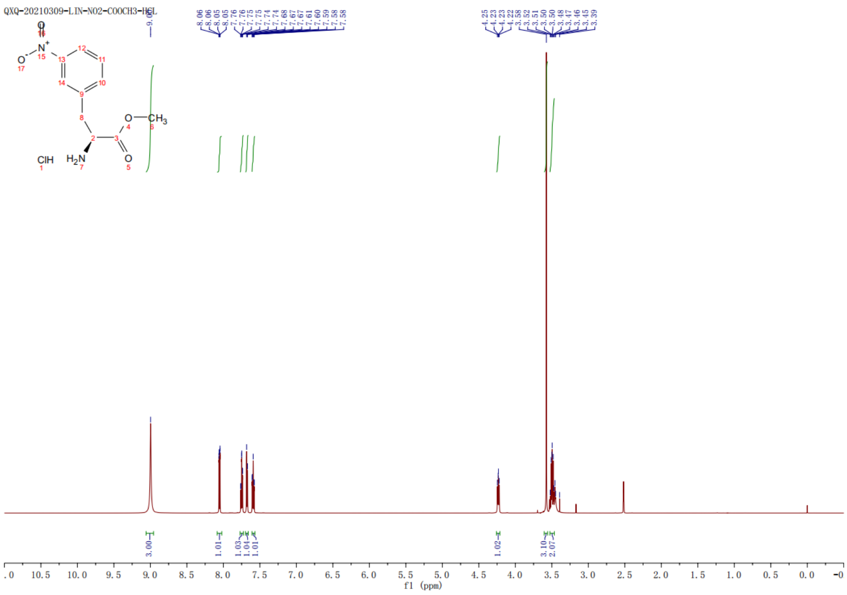

Figure S8. ^{1}H-NMR spectra of **1q**.

1r: Light yellow soild (91% yield), ^{1}H-NMR (600 MHz, DMSO-d_6) δ 8.87 (s, 3H), 8.21-8.20 (m, 1H), 8.17-8.15 (m, 1H), 7.77-7.75 (d, *J* = 12 Hz, 1H), 7.66-7.63 (t, *J* = 18 Hz, 1H), 4.41-4.39 (t, *J* = 12 Hz, 1H), 3.70 (s, 3H), 3.37-3.34 (m, 2H).

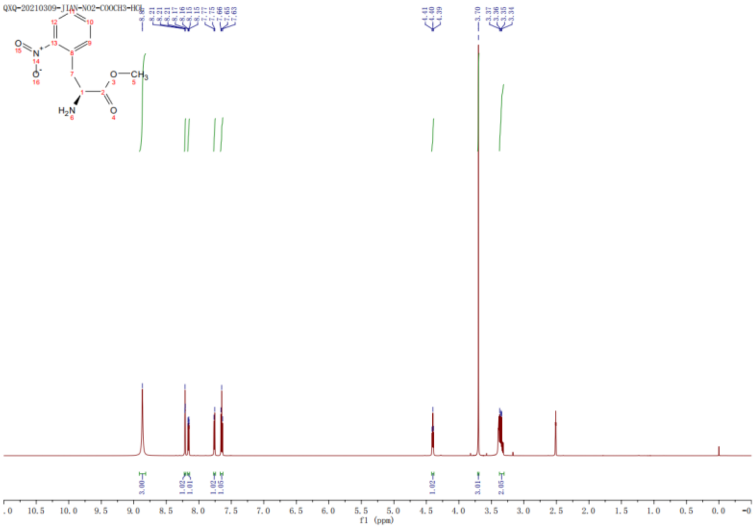

Figure S9. ^{1}H-NMR spectra of **1r**.

1s: Light yellow soild (96% yield), ^{1}H-NMR (600 MHz, DMSO-d_6) δ 8.89 (s, 3H), 7.18-7.13 (m, 4H), 4.08-4.06 (t, J = 18 Hz, 1H), 3.51 (s, 3H), 3.28-3.03 (m, 1H), 3.06-3.03 (m, 1H), 2.29 (s, 3H).

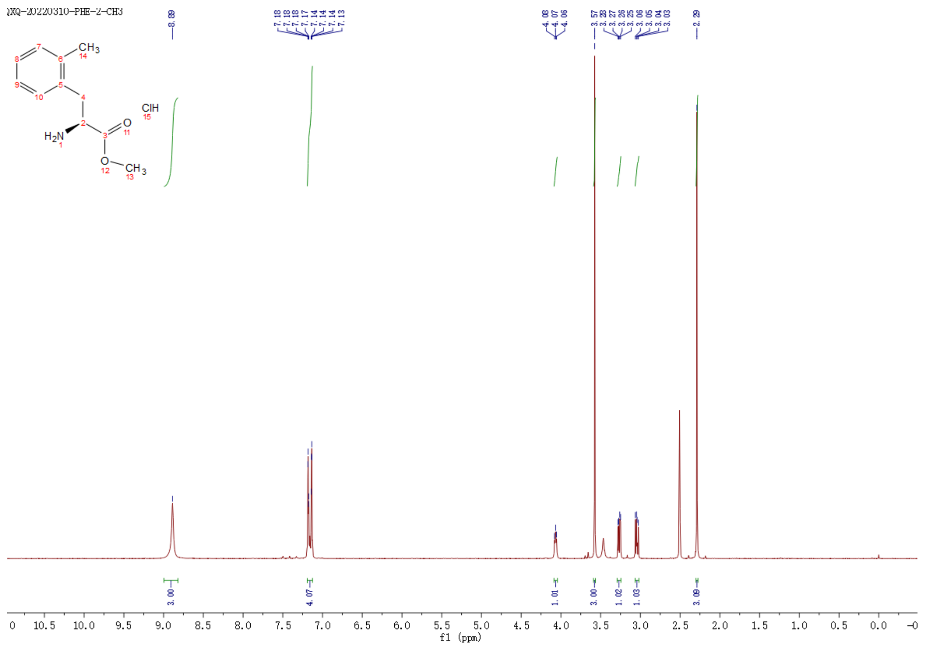

Figure S10. ^{1}H-NMR spectra of **1s**.

1t: Light yellow soild (95% yield), ^{1}H-NMR (600 MHz, DMSO-d_6) δ 8.74 (s, 3H), 7.23-7.20 (t, J = 18 Hz, 1H), 7.10-7.09 (d, J = 6 Hz, 1H), 7.05 (s, 1H), 7.10-7.09 (d, J = 6 Hz, 1H), 4.23-4.21 (t, J = 12 Hz, 1H), 3.66 (s, 3H), 3.18-3.14 (m, 1H), 3.08-3.04 (m, 1H), 2.29 (s, 3H).

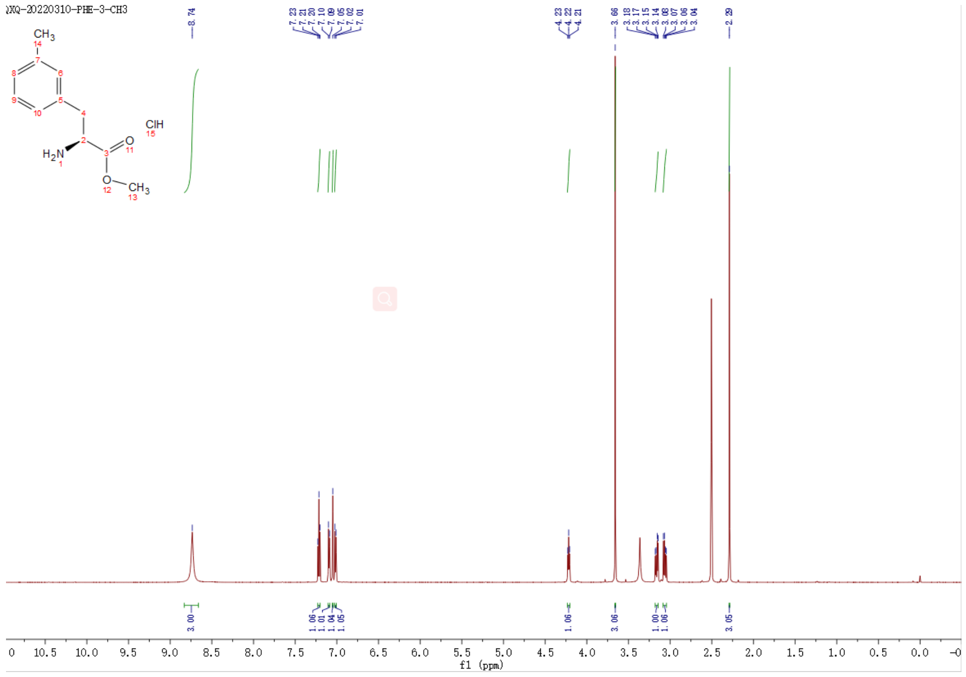

Figure S11. ^{1}H-NMR spectra of **1t**.

1u: Colorless soild (94% yield), ^{1}H-NMR (600 MHz, DMSO-d_6) δ 8.88 (s, 3H), 7.92-7.86 (m, 3H), 7.79 (s, 1H), 7.54-7.50 (m, 2H), 7.42-7.40 (m, 1H), 3.36-3.34 (t, 1H), 3.67 (s, 3H), 3.43-3.40 (m, 1H), 3.34-3.30 (m, 1H).

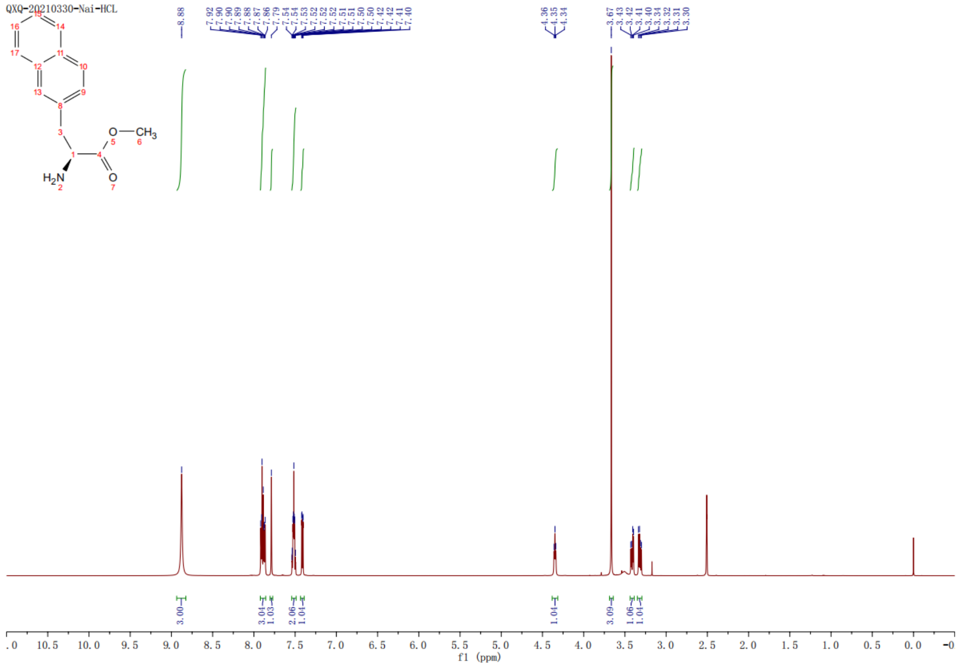

Figure S12. ^{1}H-NMR spectra of **1u**.

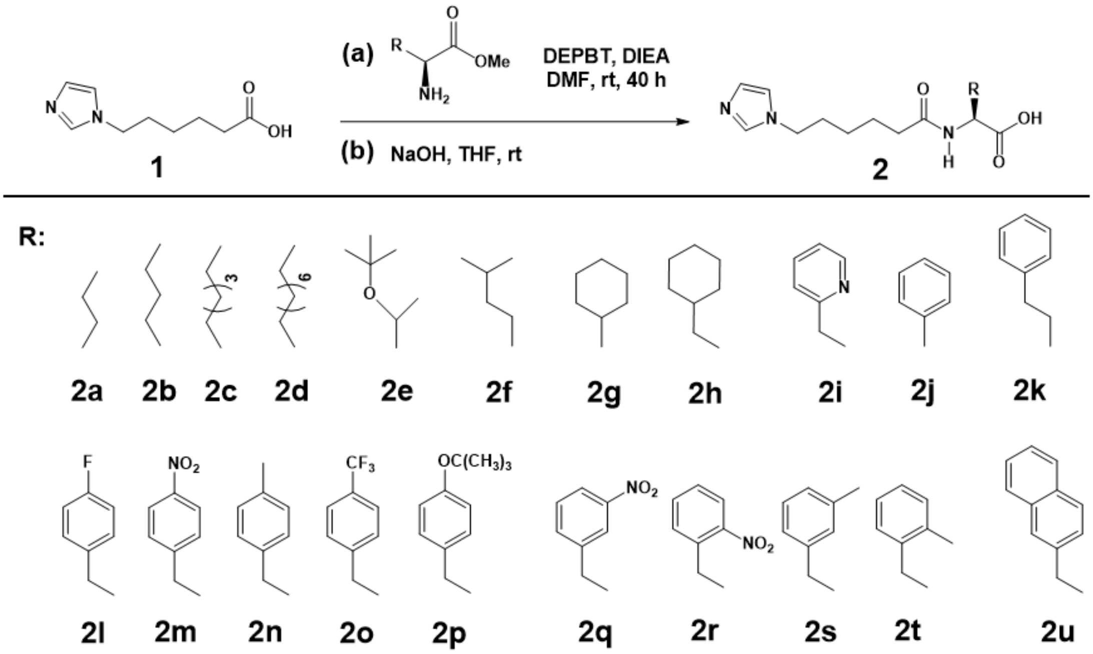

Scheme S2. Synthesis of DFSMs.

2a: Colorless oil (42% yield), ^{1}H-NMR (600 MHz, DMSO-d_6) δ 8.02-8.01 (d, *J* = 6 Hz, 1H), 7.64 (s, 1H), 7.16 (s, 1H), 6.89 (m, 1H), 4.18-4.15 (m, 1H), 3.94-3.92 (t, *J* = 12 Hz, 2H), 2.13-2.08 (m, 2H), 1.72-1.67 (m, 3H), 1.66-1.61 (m, 1H), 1.58-1.48 (m, 3H), 1.33-1.26 (m, 2H), 1.21-1.16 (m, 2H), 0.87-0.84 (t, *J* = 18 Hz, 2H).^{13}C-NMR (151 MHz, DMSO-d_6) δ172.56, 137.56, 128.45, 119.76, 51.91, 46.36, 35.26, 33.62, 30.74, 25.91, 25.13, 19.15, 13.95. LCMS (ESI): m/z $[M+H]^+$: calcd. for $C_{15}H_{26}N_3O_3$: 280.1667; found: 280.1664.

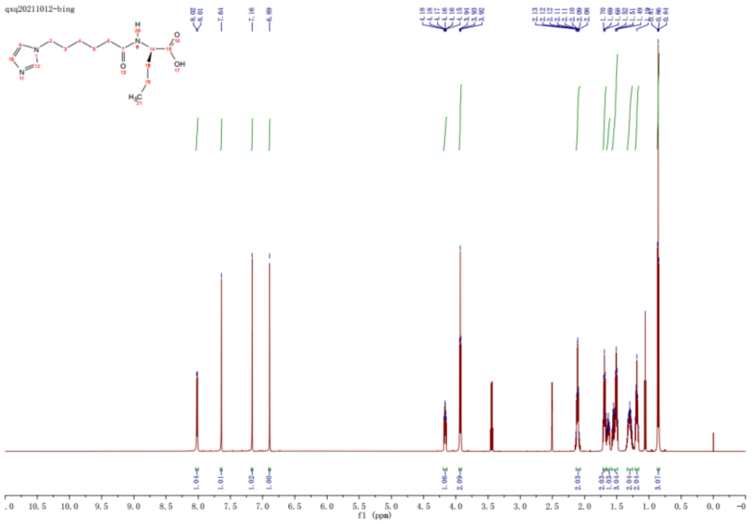

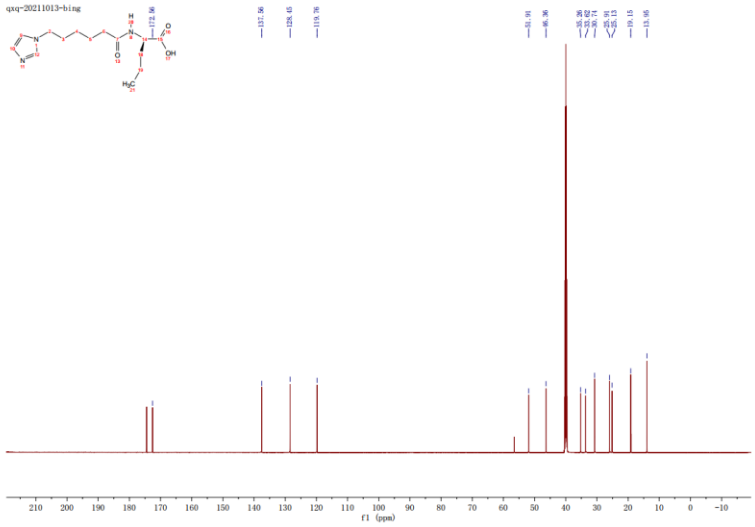

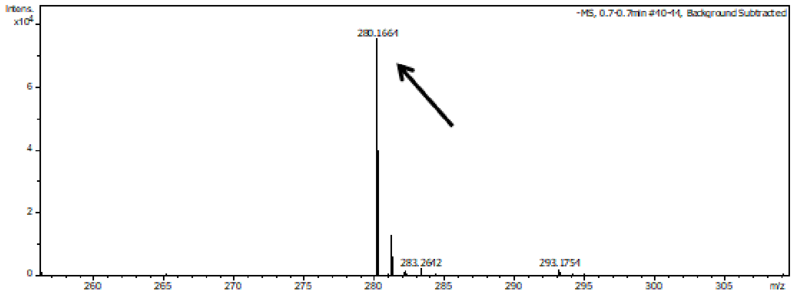

Figure S13. ^{1}H-NMR, ^{13}C-NMR and LCMS spectra of **2a**.

2b: Colorless oil (52% yield), ^{1}H-NMR (600 MHz, DMSO-d$_6$) δ 7.76-7.75 (d, J = 6 Hz, 1H), 7.60 (s, 1H), 7.15 (s, 1H), 6.87 (m, 1H), 4.07-4.06 (m, 1H), 3.93 (t, J = 12 Hz, 2H), 2.11-2.08 (m, 3H), 1.70-1.66 (m, 3H), 1.52-1.49 (m, 3H), 1.25 – 1.19 (m, 6H), 0.85-0.83 (m, 3H).^{13}C-NMR (151 MHz, DMSO-d$_6$) δ174.94, 171.98, 137.62, 128.66, 119.69, 53.10, 46.30, 35.52, 31.97, 30.81, 28.01, 25.97, 25.23, 22.40, 14.37. LCMS (ESI): m/z [M+H]$^+$: calcd. for $C_{15}H_{26}N_3O_3$: 296.1969; found: 296.1969.

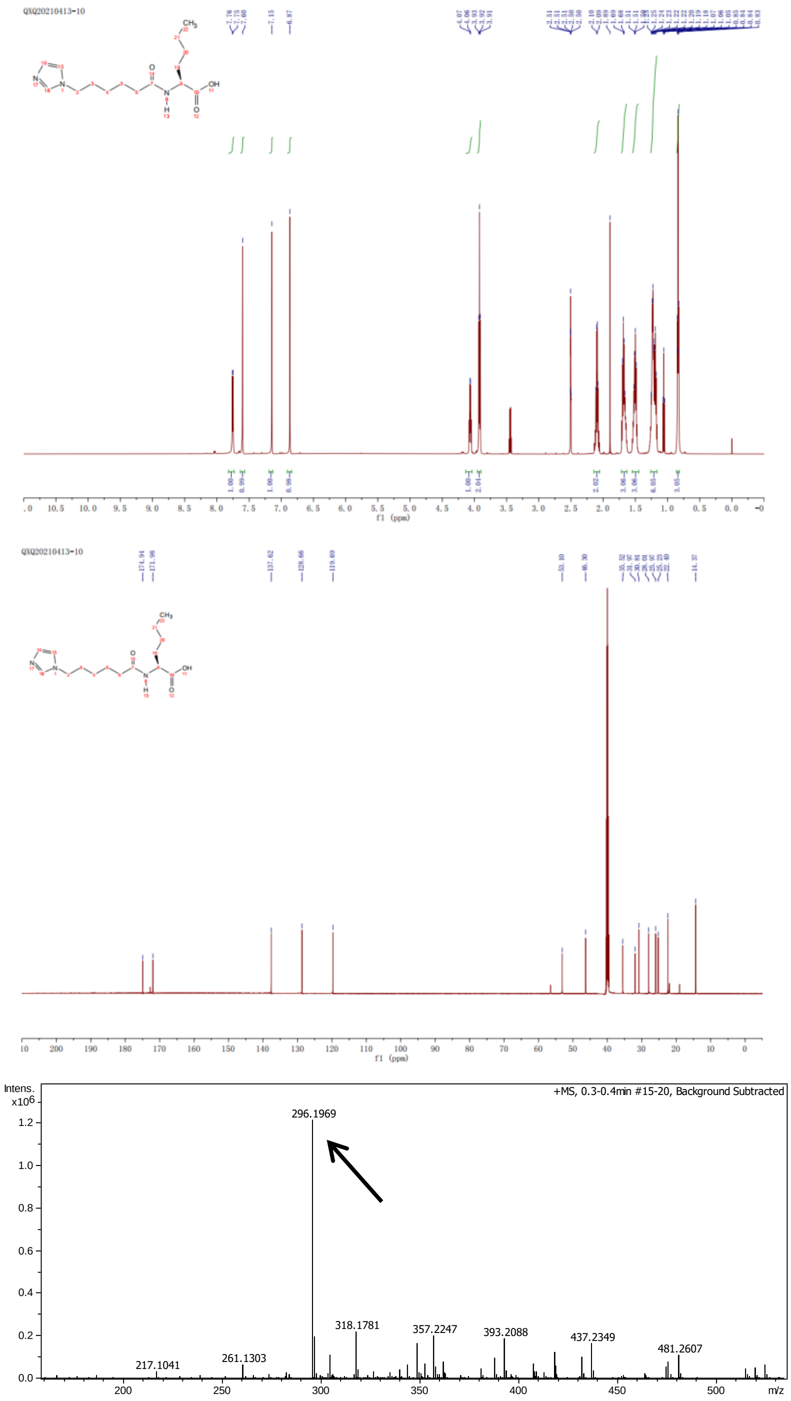

Figure S14. ^{1}H-NMR, ^{13}C-NMR and LCMS spectra of **2b**.

2c: Colorless oil (63% yield), ^{1}H-NMR (600 MHz, DMSO-d_6) δ 8.03 (d, *J* = 6 Hz, 1H), 7.64 (s, 1H), 7.16 (s, 1H), 6.89 (m, 1H), 4.17-4.13 (m, 1H), 3.94 (t, *J* = 12 Hz, 2H), 2.14-2.08 (m, 3H), 1.71-1.66 (m, 3H), 1.53-1.49 (m, 3H), 1.27 – 1.23 (m, 6H), 0.86-0.84 (m, 3H).^{13}C-NMR (151 MHz, DMSO-d_6) δ174.44, 172.55, 119.75, 52.18, 46.35, 35.25, 31.55, 30.74, 28.68, 25.91, 25.79, 25.14, 22.46, 14.38. LCMS (ESI): m/z $[M+H]^+$: calcd. for $C_{17}H_{30}N_3O_3$: 324.2281; found: 324.2282.

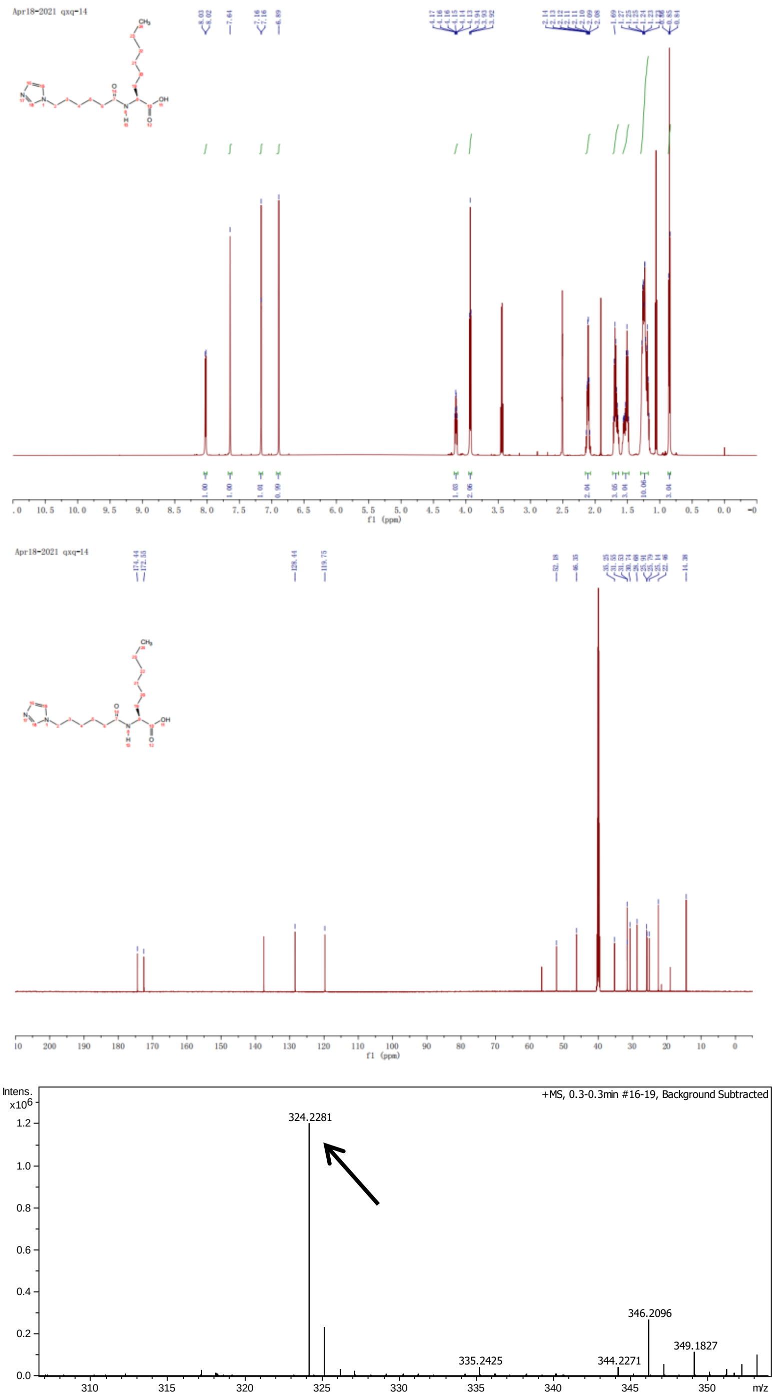

Figure S15. ^{1}H-NMR, ^{13}C-NMR and LCMS spectra of **2c**.

2d: Colorless oil (71% yield), ^{1}H-NMR (600 MHz, DMSO-d_6) δ 9.23 (s, 1H), 8.11 (d, *J* = 12 Hz, 1H), 7.81 (s, 1H), 7.68 (m, 1H), 4.20-4.18 (t, *J* = 12 Hz, 3H), 4.16-4.14 (m, 1H), 2.15-2.13 (t, *J* = 12 Hz, 2H), 1.84-1.79 (m, 2H), 1.66-1.64 (m, 1H), 1.57-1.53 (m, 3H), 1.27-1.23 (m, 15H), 0.86-0.84 (m, 3H). ^{13}C-NMR (151 MHz, DMSO-d_6) δ174.32, 172.57, 135.51, 122.41, 120.17, 52.16, 48.75, 35.07, 31.71, 31.41, 29.60, 29.27, 29.07, 29.00, 25.84, 25.48, 24.94, 22.55, 14.42. LCMS (ESI): m/z $[M+H]^{+}$: calcd. for $C_{19}H_{34}N_3O_3$: 352.2596; found: 352.2595.

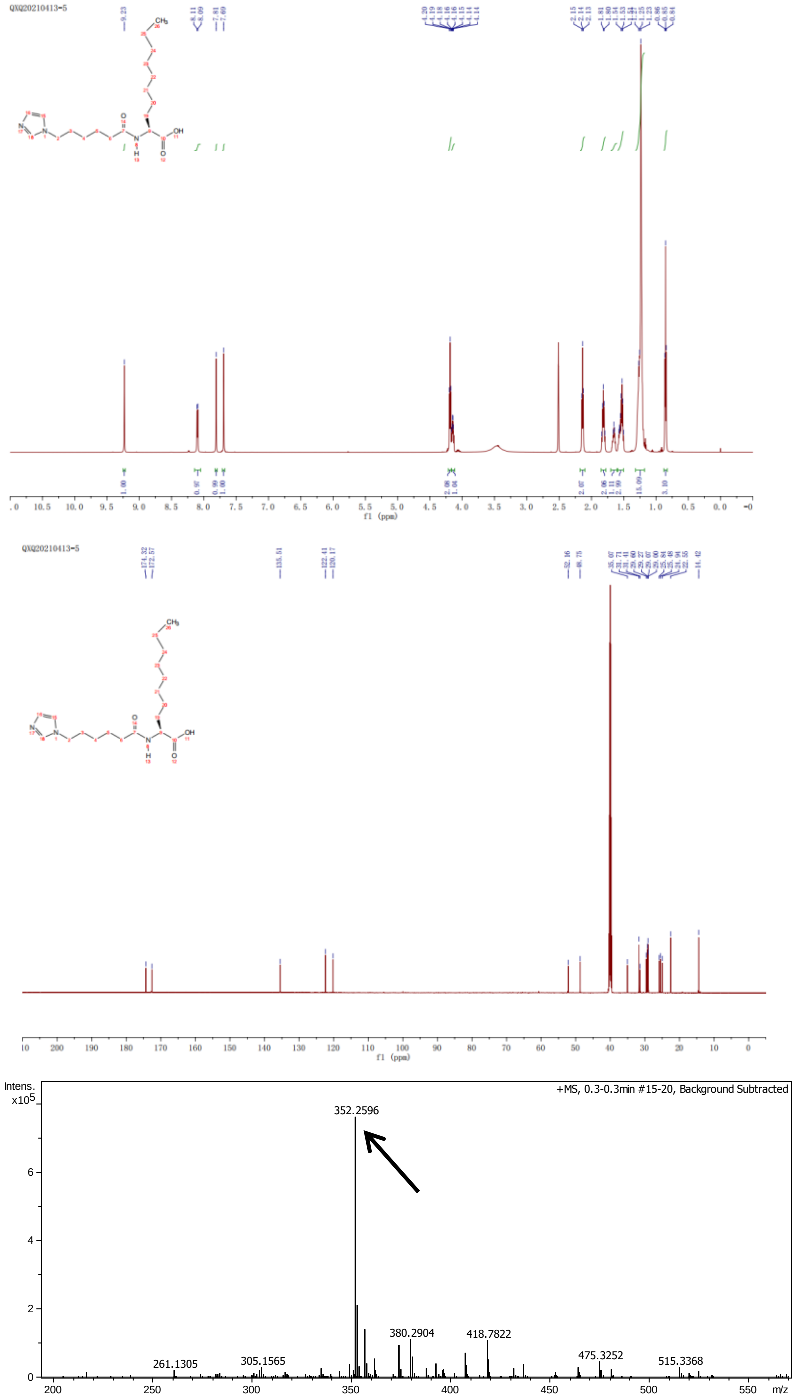

Figure S16. ^{1}H-NMR, ^{13}C-NMR and LCMS spectra of **2d**.

2e: Colorless oil (58% yield), ^{1}H-NMR (600 MHz, DMSO-d_6) δ 7.88 (d, *J* = 12 Hz, 1H), 7.61 (s, 1H), 7.15 (s, 1H), 6.87 (m, 1H), 4.11-4.07 (m, 1H), 3.94-3.91 (t, *J* = 18 Hz, 2H), 2.13-2.08 (m, 2H), 1.71-1.65 (m, 2H), 1.52-1.47 (m, 4H), 1.20-1.107 (m, 4H),0.84-0.83 (d, *J* = 6 Hz, 6H). ^{13}C-NMR (151 MHz, DMSO-d_6) δ174.73, 172.21, 137.60, 128.63, 119.70, 52.94, 46.30, 35.41, 34.93, 30.79, 29.90, 27.72, 25.94, 25.20, 23.02, 22.76. LCMS (ESI): m/z [M+H]$^{+}$: calcd. for $C_{16}H_{28}N_3O_3$: 310.2125; found:310.2125.

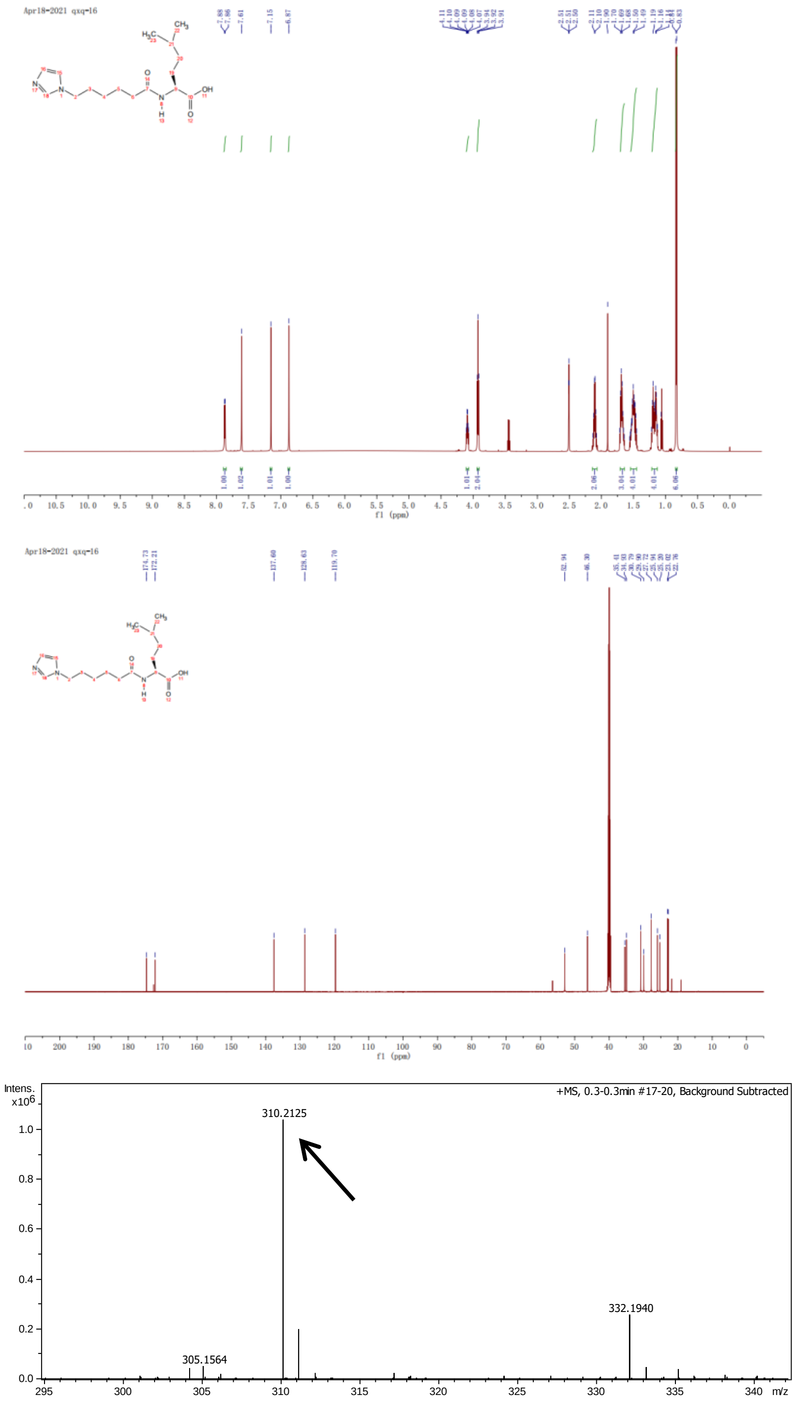

Figure S17. ^{1}H-NMR, ^{13}C-NMR and LCMS spectra of **2e**.

2f: Colorless oil (49% yield), ^{1}H-NMR (600 MHz, DMSO-d_6) δ 7.62 (s, 1H), 7.54 (d, J = 6 Hz, 1H), 7.15 (s, 1H), 6.87 (m, 1H), 4.28-4.26 (q, 1H), 4.10-4.07 (m, 1H), 3.94-3.92 (t, J = 12 Hz, 2H), 2.23-2.19 (m, 2H), 1.71-1.68 (m, 2H), 1.52-1.50 (m, 2H), 1.11 (s, 9H),1.05-1.03 (d, J = 12 Hz, 2H).^{13}C-NMR (151 MHz, DMSO-d_6) δ172.91, 172.74, 137.59, 128.56, 119.71, 73.61, 67.64, 56.48, 46.33, 35.18, 30.80, 28.76, 25.98, 25.21, 19.03. LCMS (ESI): m/z $[M+H]^+$: calcd. for $C_{18}H_{24}N_3O_3$: 339.2210; found: 340.2229.

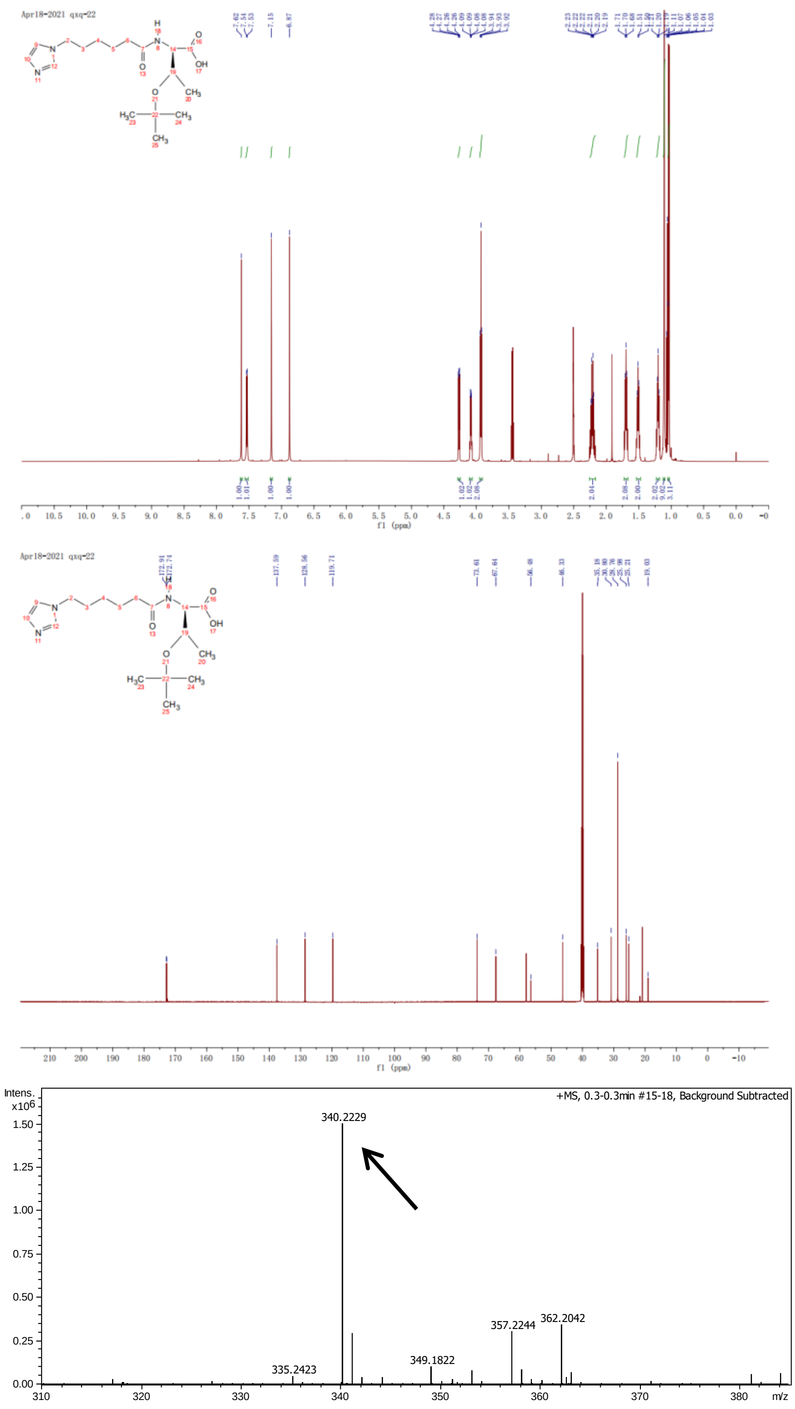

Figure S18. ^{1}H-NMR, ^{13}C-NMR and LCMS spectra of **2f**.

2g: Colorless oil (67% yield), ^{1}H-NMR (600 MHz, DMSO-d_6) δ 7.86 (d, J = 6 Hz, 1H), 7.61 (s, 1H), 7.15 (s, 1H), 6.87 (m, 1H), 4.11-4.08 (m, 1H), 3.93-3.91 (t, *J* = 12 Hz, 2H), 2.15-2.11 (m, 2H), 1.70-1.66 (m, 5H), 1.57-1.49 (m, 5H), 1.21-1.14 (m, 4H),1.00-0.97 (m, 2H). ^{13}C-NMR (151 MHz, DMSO-d_6) δ173.86, 172.47, 137.60, 128.62, 119.69, 56.49, 46.32, 35.30, 30.78, 29.83, 26.22, 26.11, 26.09, 25.98, 25.25, 21.67. LCMS (ESI): m/z $[M+H]^+$: calcd. for $C_{17}H_{28}N_3O_3$: 322.2126; found: 322.2125.

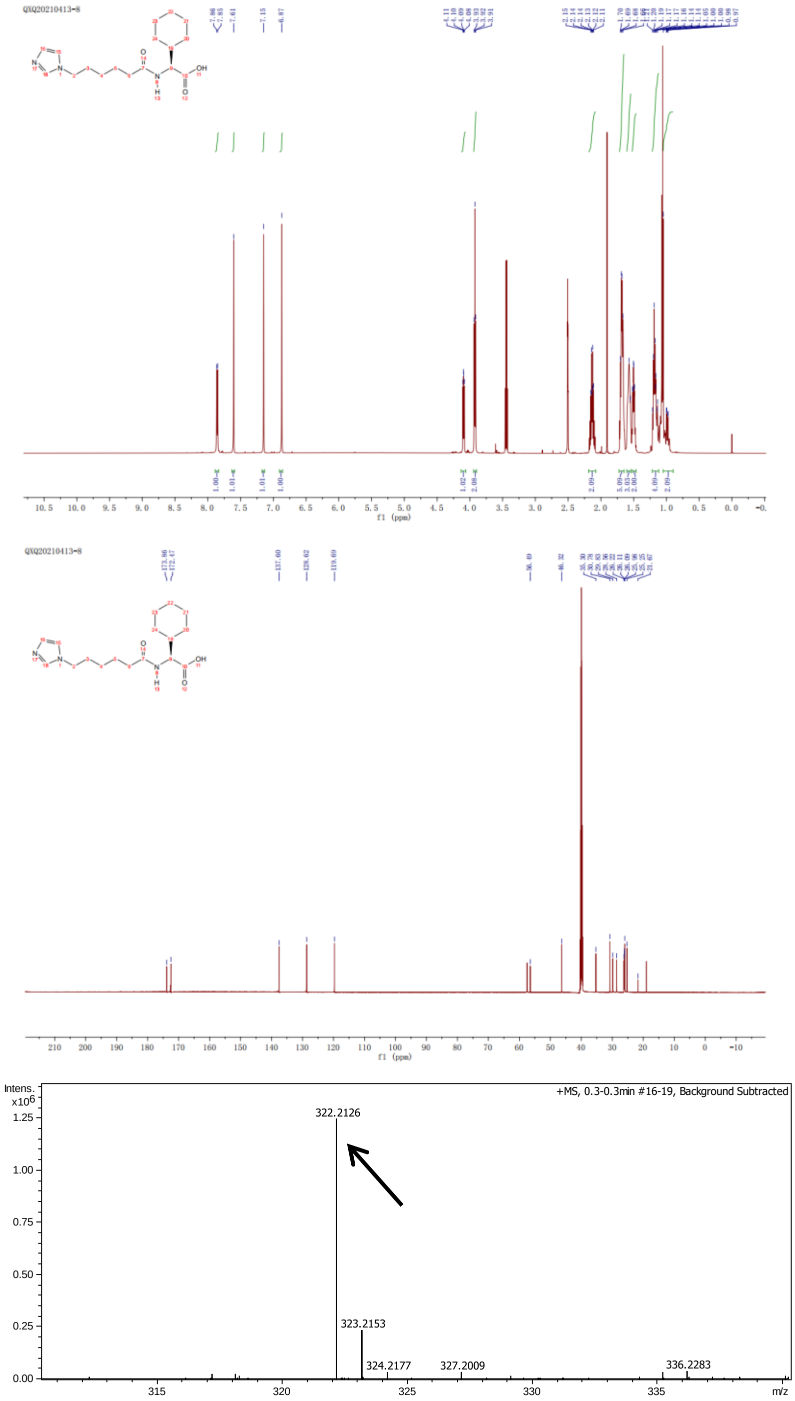

Figure S19. ^{1}H-NMR, ^{13}C-NMR and LCMS spectra of **2g**.

2h: Colorless oil (72% yield), ^{1}H-NMR (600 MHz, DMSO-d6) δ 8.02 (d, *J* = 6 Hz, 1H), 7.61 (s, 1H), 7.15 (s, 1H), 6.88 (m, 1H), 4.24-4.23 (m, 1H), 3.94-3.91 (t, *J* = 18 Hz, 2H), 2.12-2.08 (m, 2H), 1.71-1.61 (m, 7H), 1.52-1.47 (m, 4H), 1.30-1.11 (m, 6H), 0.94-0.90 (m, 1H), 0.84-0.78 (m, 1H). ^{13}C-NMR (151 MHz, DMSO-d_6) δ174.94, 172.49, 137.59, 128.63, 119.70, 49.89, 46.31, 35.29, 34.08, 33.61, 31.98, 30.75, 26.48, 26.27, 26.08, 25.87, 25.14. LCMS (ESI): m/z [M+H]$^+$: calcd. for $C_{18}H_{23}BrN_3O_3$: 408.0908; found: 408.0917.

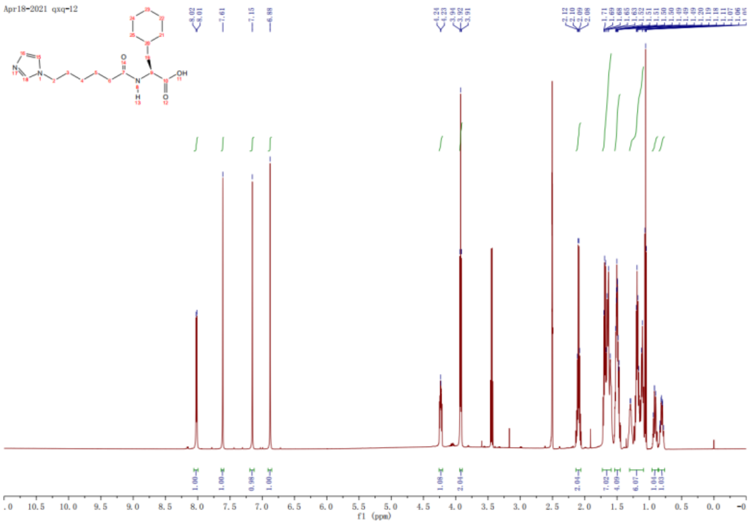

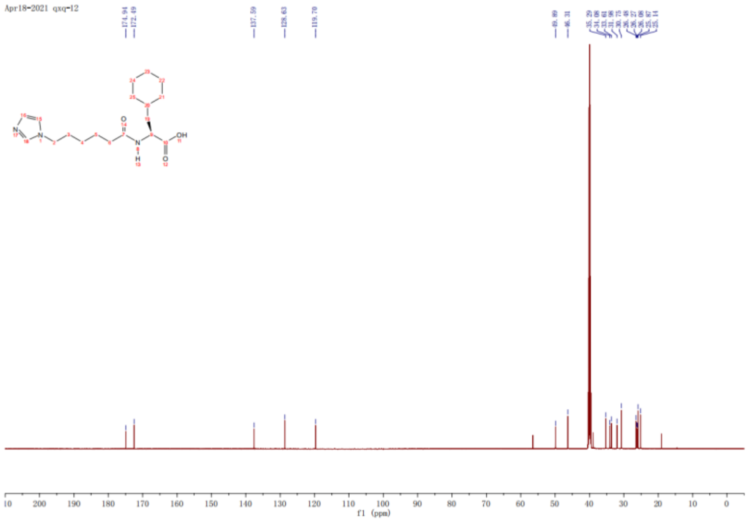

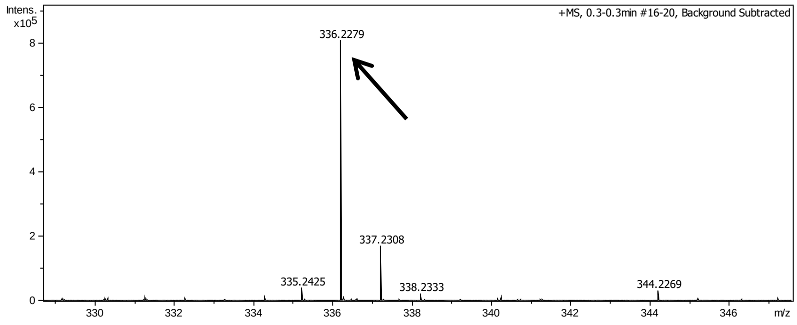

Figure S20. ^{1}H-NMR, ^{13}C-NMR and LCMS spectra of **2h**.

2i: Colorless oil (61% yield), ^{1}H-NMR (600 MHz, DMSO-d_6) δ 8.48 (d, *J* = 6 Hz, 1H), 8.40 (s, 1H), 8.20 (d, *J* = 12 Hz, 1H), 7.71-7.68 (m, 1H), 7.46 (s, 1H), 7.28 (d, *J* = 12 Hz, 2H), 7.23-7.21 (m, 1H), 4.67-4.66 (m, 1H), 4.01-3.99 (t, *J* = 12 Hz, 2H), 3.20-3.17 (m, 1H), 3.03-3.00 (m, 1H), 2.05-2.02 (m, 2H), 1.69-1.67 (m, 2H), 1.43-1.41 (m, 2H), 1.09-1.06 (m, 2H). ^{13}C-NMR (151 MHz, DMSO-d_6) δ173.71, 172.40, 158.01, 149.42, 136.80, 136.56, 124.42, 124.18, 122.18, 121.05, 52.28, 47.50, 35.20, 30.12, 25.51, 24.92. LCMS (ESI): m/z $[M+H]^+$: calcd. for $C_{17}H_{23}N_4O_3$: 331.1764; found:331.1765.

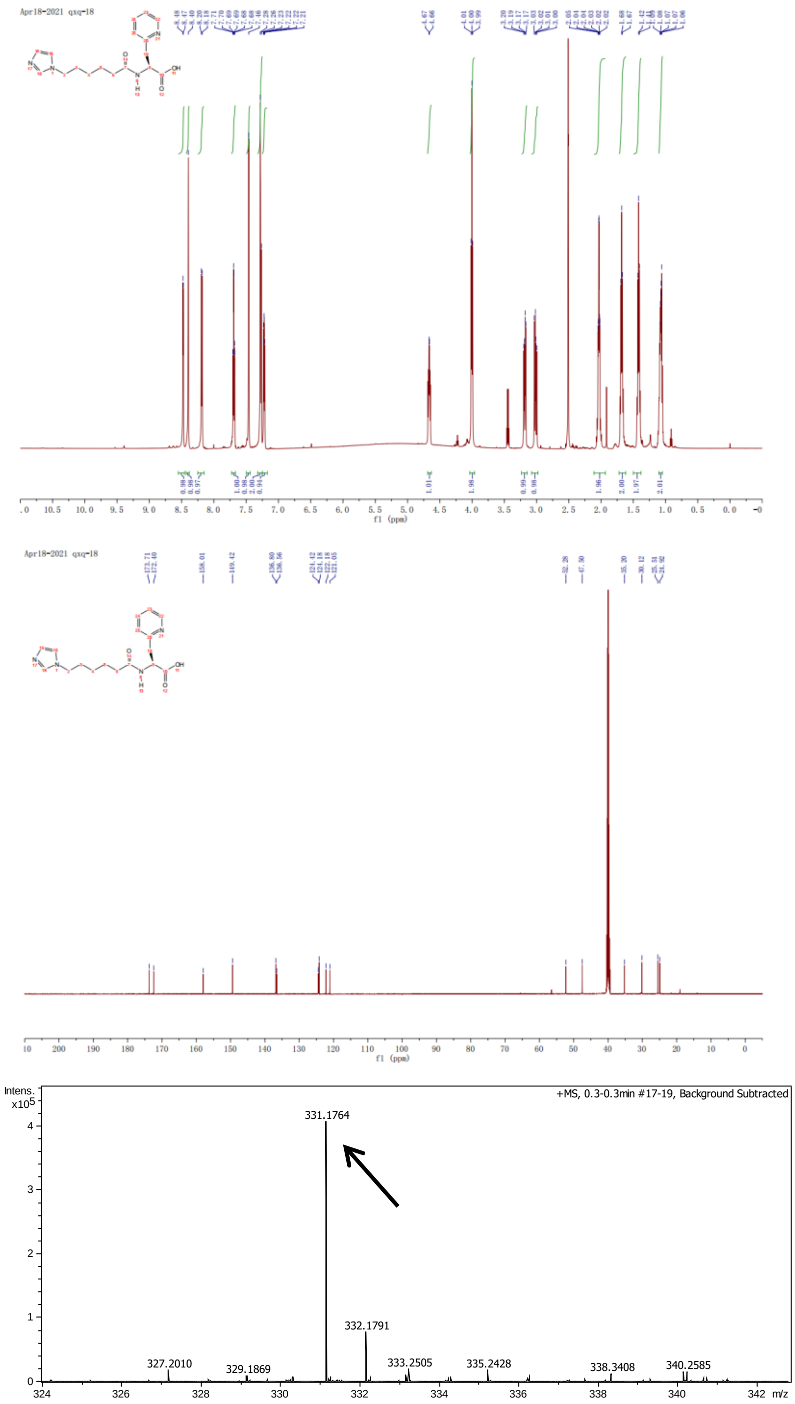

Figure S21. ^{1}H-NMR, ^{13}C-NMR and LCMS spectra of **2i**.

2j: Colorless oil (81% yield), ^{1}H-NMR (600 MHz, DMSO-d_6) δ 8.50 (d, J = 6 Hz, 1H), 7.71 (s, 1H), 7.39-7.30 (m, 5H), 7.18 (s, 1H), 7.46 (s, 1H), 6.92 (s, 1H), 5.31-5.30 (d, J = 6 Hz, 1H), 3.94-3.91 (t, J = 18 Hz, 2H), 2.19-2.17 (m, 2H), 1.71-1.66 (m, 2H), 1.53-1.48 (m, 2H), 1.21-1.17 (m, 2H). ^{13}C-NMR (151 MHz, DMSO-d_6) δ172.57, 172.29, 138.34, 128.85, 128.13, 128.05, 128.00, 119.89, 56.86, 46.46, 35.10, 30.69, 25.93, 25.11. LCMS (ESI): m/z $[M+H]^{+}$: calcd. for $C_{17}H_{22}N_3O_3$: 316.1659; found: 316.1656.

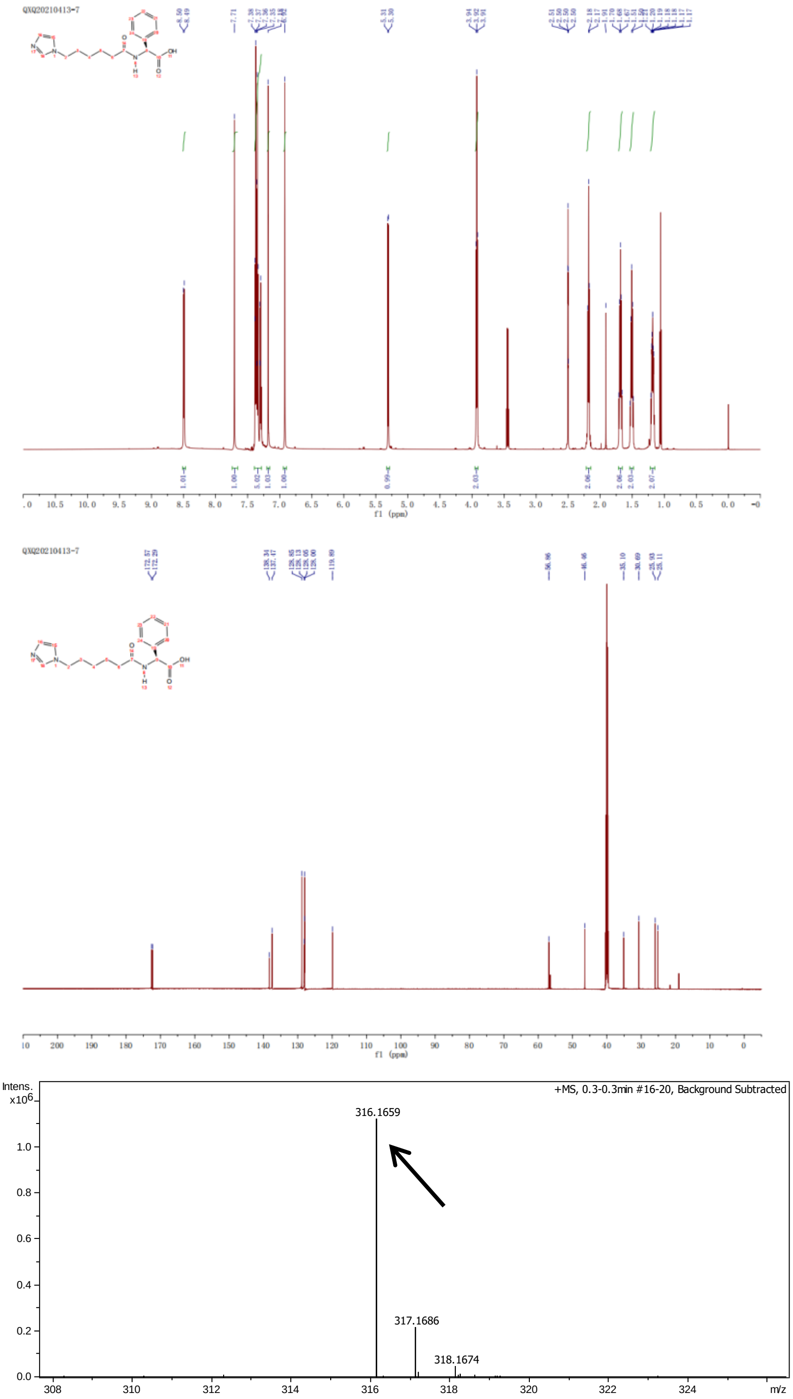

Figure S22. ^{1}H-NMR, ^{13}C-NMR and LCMS spectra of **2j**.

2k: Colorless oil (76% yield), ^{1}H-NMR (600 MHz, DMSO-d_6) δ 8.21 (d, *J* = 6 Hz, 1H), 8.10 (s, 1H), 7.35 (s, 1H), 7.30 (t, *J* = 18 Hz, 2H), 7.20-7.18 (m, 3H), 7.12 (s, 1H), 4.16-4.12 (m, 1H), 4.02-4.00 (t, *J* = 12 Hz, 2H), 2.64-2.57 (m, 2H), 2.18-2.14 (m, 2H), 1.97-1.95 (m, 1H), 1.88-1.85 (m, 1H), 1.76-1.72 (m, 2H), 1.56-1.52 (m, 2H), 1.26-1.22 (m, 2H). ^{13}C-NMR (151 MHz, DMSO-d_6) δ174.22, 172.71, 141.55, 136.99, 128.82, 126.08, 120.54, 51.78, 47.06, 35.26, 33.31, 31.99, 30.43, 25.82, 25.08. LCMS (ESI): m/z $[M+H]^+$: calcd. for $C_{19}H_{26}N_3O_3$: 344.1968; found: 344.1969.

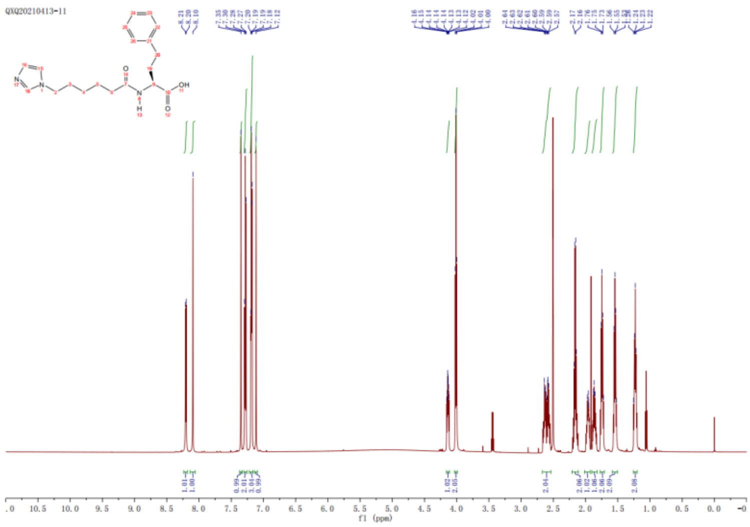

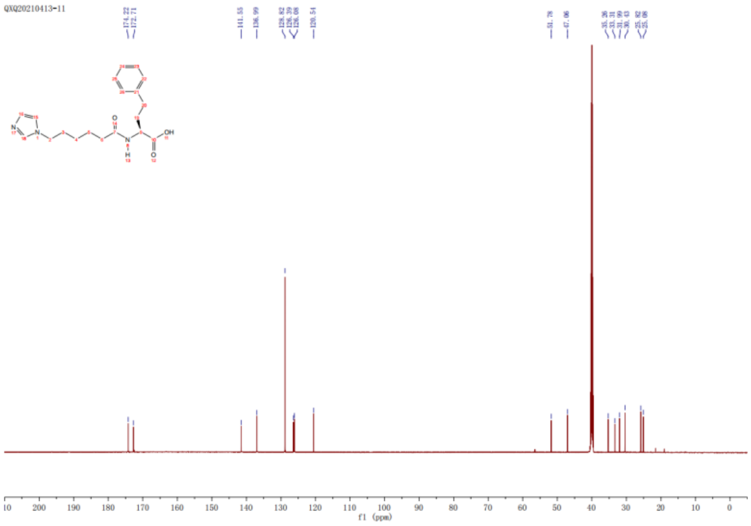

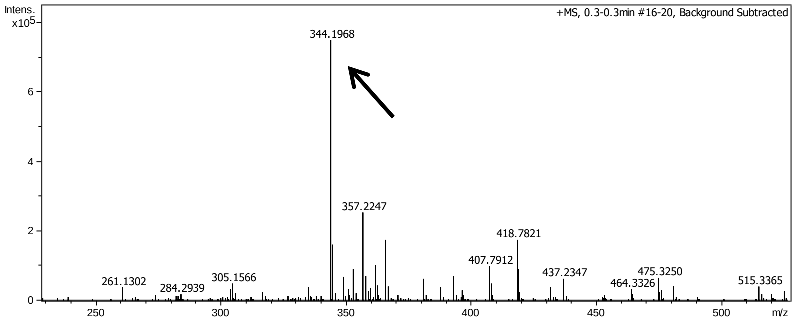

Figure S23. ^{1}H-NMR, ^{13}C-NMR and LCMS spectra of **2k**.

2l: Colorless oil (83% yield), ^{1}H-NMR (600 MHz, DMSO-d$_6$) δ 8.10 (d, *J* = 6 Hz, 1H), 7.64 (s, 1H), 7.26-7.24 (m, 2H), 7.15 (s, 1H), 7.09 (t, *J* = 18 Hz, 2H), 6.90 (s, 1H), 4.44-4.40 (m, 1H), 3.90-38-.87 (t, *J* = 18 Hz, 2H), 3.07-3.04 (m, 1H), 2.84-2.80 (m, 1H), 2.05-2.02 (t, *J* = 18 Hz, 2H), 1.64-1.62 (m, 2H), 1.42-1.40 (m, 2H), 1.11-1.03 (m, 2H). ^{13}C-NMR (151 MHz, DMSO-d$_6$) δ173.72, 172.35, 160.63, 137.52, 134.56, 134.54, 131.44, 131.38, 128.40, 119.76, 115.28, 115.14, 53.90, 46.32, 36.48, 35.34, 30.73, 25.79, 25.05. LCMS (ESI): m/z [M+H]$^+$: calcd. For $C_{18}H_{23}FN_3O_3$: 348.1723; found: 348.1718.

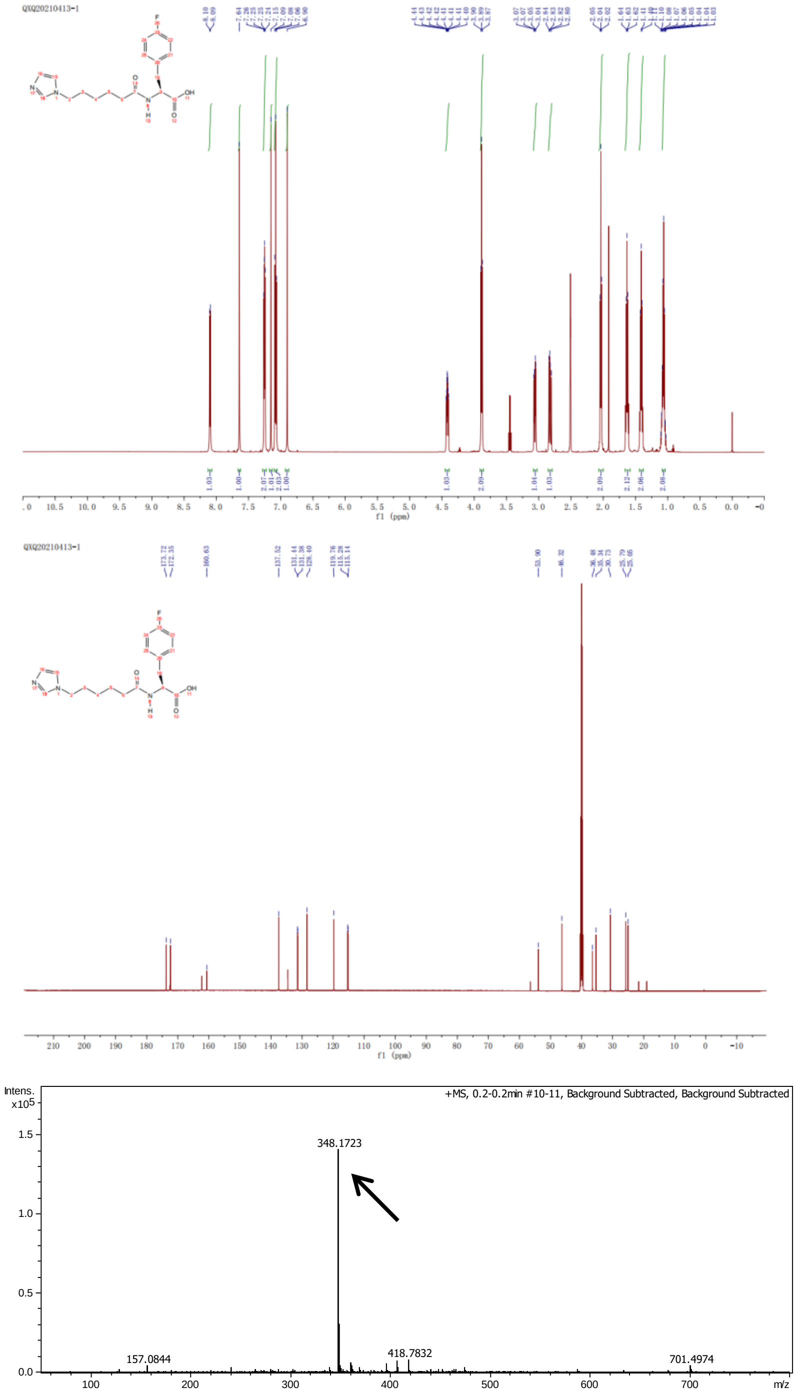

Figure S24. ^{1}H-NMR, ^{13}C-NMR and LCMS spectra of **2l**.

2m: Colorless solid (88% yield), ^{1}H-NMR (600 MHz, DMSO-d$_6$) δ 8.23 (d, *J* = 6 Hz, 1H), 8.15 (d, *J* = 6 Hz, 2H), 7.71 (s, 1H), 7.53 (d, *J* = 6 Hz, 2H), 7.18 (s, 1H), 6.94 (s, 1H), 4.55-4.51 (m, 1H), 3.90-3.87 (t, *J* = 18 Hz, 2H), 3.24-3.21 (m, 1H), 3.01-2.97 (m, 1H), 2.06-2.01 (m, 2H), 1.65-1.60 (m, 2H), 1.43-1.38 (m, 2H), 1.10-1.01 (m, 2H). ^{13}C-NMR (151 MHz, DMSO-d$_6$) δ173.29, 172.43, 146.88, 146.69, 137.40, 130.99, 127.96, 123.62, 119.88, 53.26, 46.41, 37.05, 35.29, 30.64, 25.75, 24.97. LCMS (ESI): m/z [M+H]$^+$: calcd. for $C_{18}H_{23}N_4O_5$: 375.1665; found: 375.1663.

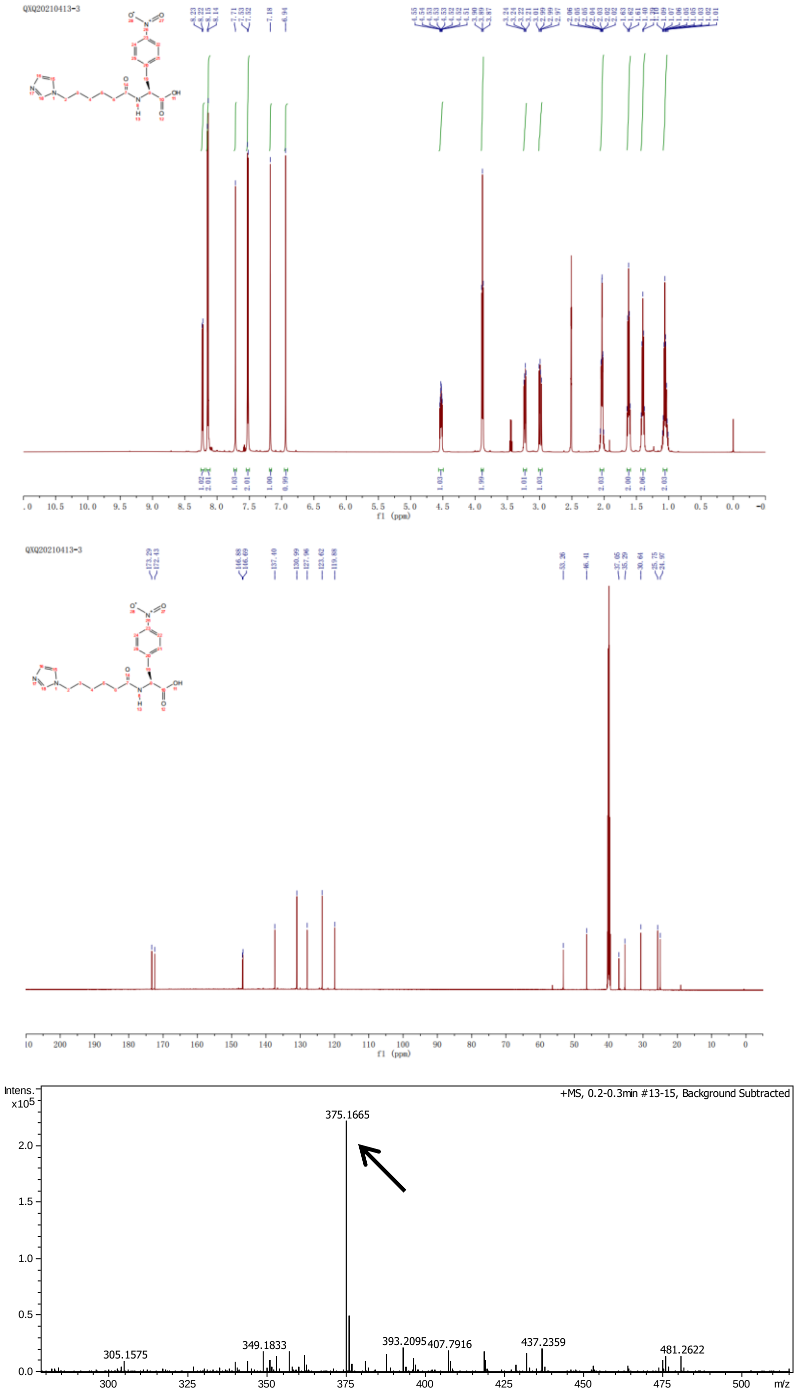

Figure S25. ^{1}H-NMR, ^{13}C-NMR and LCMS spectra of **2m**.

2n: Colorless oil (79% yield), ^{1}H-NMR (600 MHz, DMSO-d_6) δ 8.11 (d, J = 6 Hz, 1H), 7.83 (s, 1H), 7.23 (s, 1H), 7.11 (d, J = 6 Hz, 2H), 7.07 (d, J = 6 Hz, 2H), 7.00 (s, 1H), 4.42-4.38 (m, 1H), 3.92-3.90 (t, J = 12 Hz, 2H), 3.01-2.98 (m, 1H), 2.81-2.77 (m, 1H), 2.24 (s, 3H), 2.05-2.03 (t, J = 12 Hz, 2H), 1.66-1.63 (m, 2H), 1.43-1.41 (m, 2H), 1.09-1.08 (m, 2H). ^{13}C-NMR (151 MHz, DMSO-d_6) δ173.75, 172.44, 137.27, 135.72, 135.14, 129.42, 129.16, 127.37, 120.10, 53.87, 46.63, 36.86, 35.26, 30.56, 25.73, 25.00, 21.10. LCMS (ESI): m/z [M+H]$^{+}$: calcd. for $C_{19}H_{26}N_3O_3$: 344.1969; found: 344.1969.

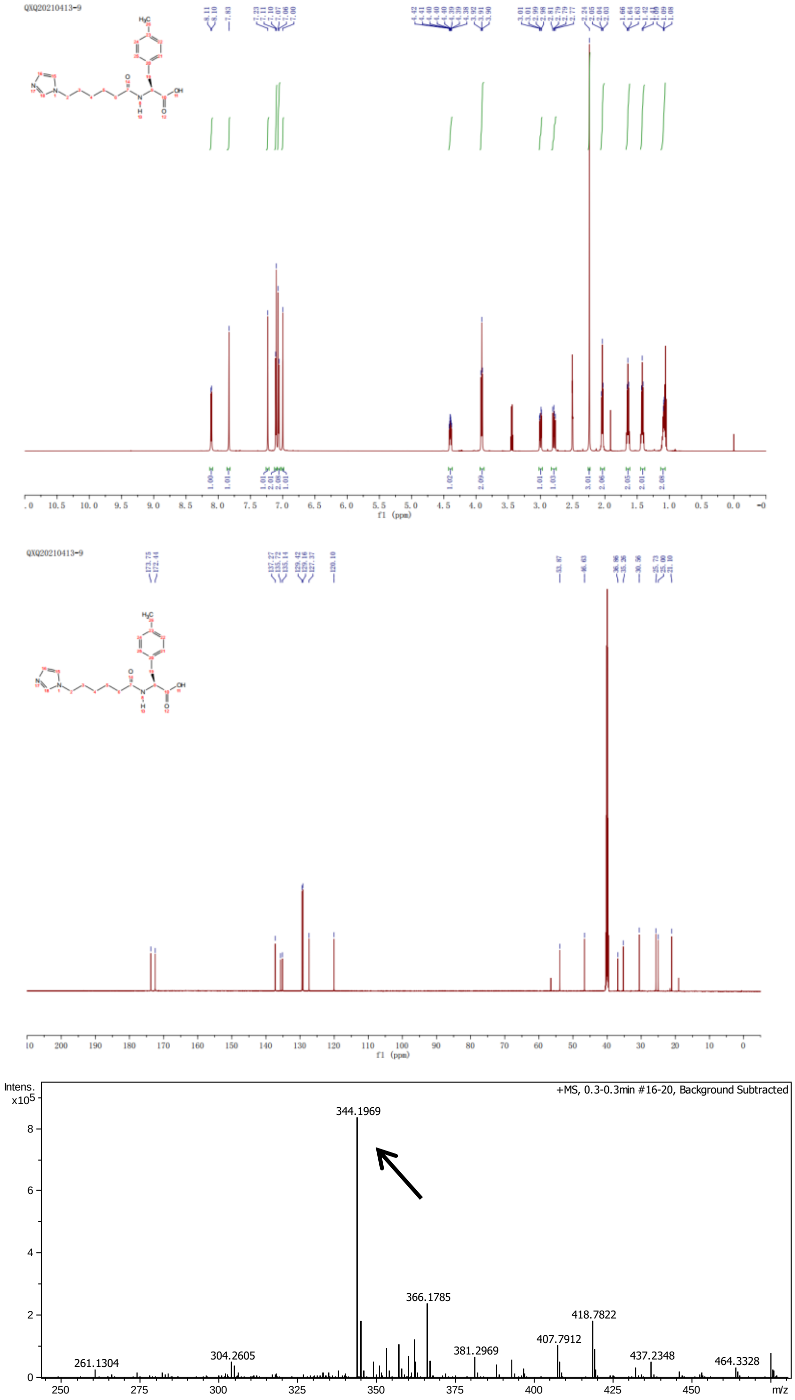

Figure S26. ^{1}H-NMR, ^{13}C-NMR and LCMS spectra of **2n**.

2o: Colorless oil (89% yield), ^{1}H-NMR (600 MHz, DMSO-d_6) δ 8.61 (s, 1H), 8.27 (d, *J* = 12 Hz, 1H), 7.64 (d, *J* = 12 Hz, 1H), 7.54 (s, 1H), 7.48 (d, *J* = 12 Hz, 2H), 7.39 (s, 1H), 4.52-4.49 (m, 1H), 4.05-4.03 (t, *J* = 12 Hz, 2H), 3.18-3.15 (m, 1H), 2.97-2.93 (m, 1H), 2.07-2.04 (m, 2H), 1.71-1.68 (m, 2H), 1.43-1.40 (m, 2H), 1.10-1.05 (m, 2H). ^{13}C-NMR (151 MHz, DMSO-d_6) δ173.32, 172.48, 143.29, 136.26, 130.46, 125.38, 125.36, 123.27, 123.27, 121.40, 53.32, 47.77, 36.91, 35.14, 29.94, 25.46, 24.83. LCMS (ESI): m/z [M+H]$^+$: calcd. for $C_{19}H_{23}F_3N_3O_3$: 398.1681; found: 398.1686.

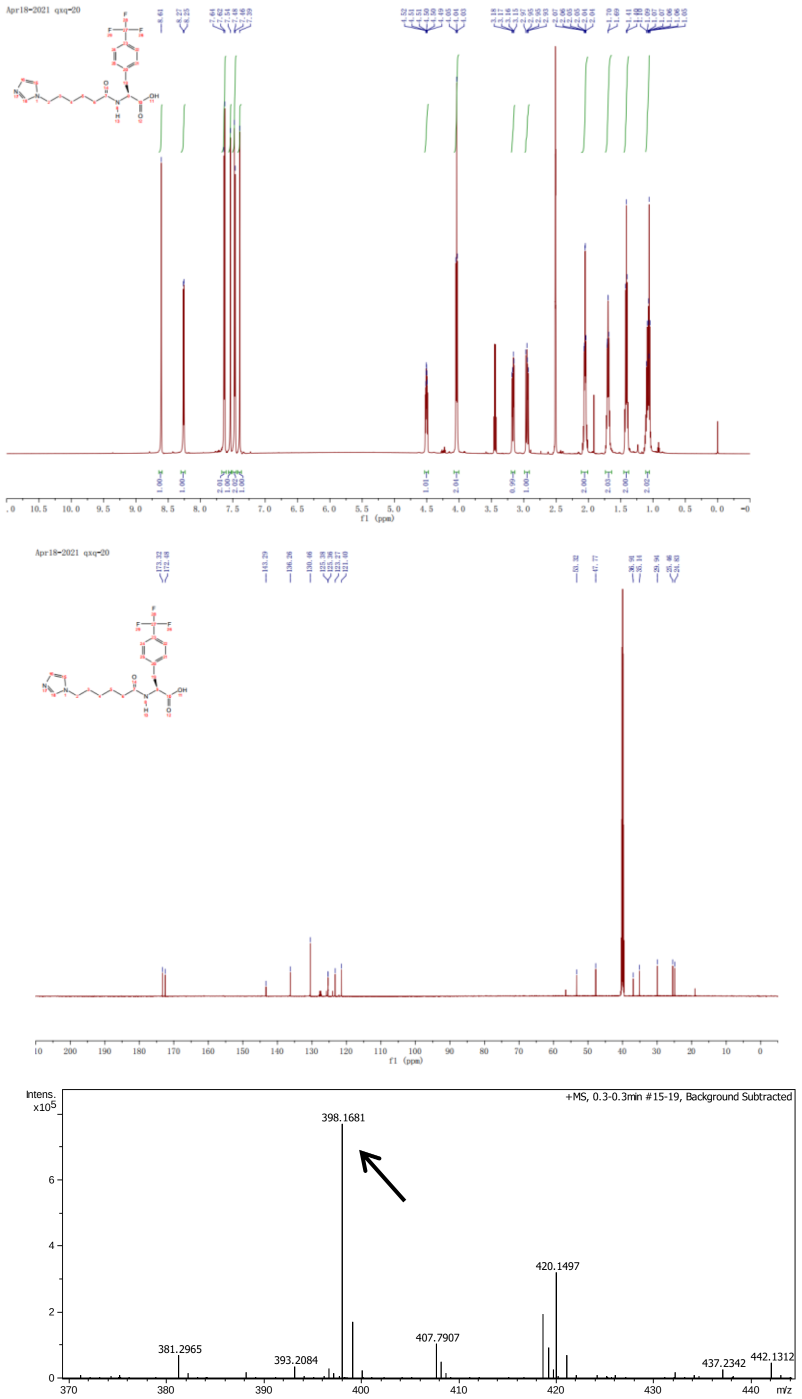

Figure S27. ^{1}H-NMR, ^{13}C-NMR and LCMS spectra of **2o**.

2p: Colorless oil (74% yield), ^{1}H-NMR (600 MHz, DMSO-d_6) δ 8.61 (s, 1H), 8.27 (d, *J* = 12 Hz, 1H), 7.64 (d, *J* = 12 Hz, 1H), 7.54 (s, 1H), 7.48 (d, *J* = 12 Hz, 2H), 7.39 (s, 1H), 4.52-4.49 (m, 1H), 4.05-4.03 (t, *J* = 12 Hz, 2H), 3.18-3.15 (m, 1H), 2.97-2.93 (m, 1H), 2.07-2.04 (m, 2H), 1.71-1.68 (m, 2H), 1.43-1.40 (m, 2H), 1.10-1.05 (m, 2H). ^{13}C-NMR (151 MHz, DMSO-d_6) δ173.32, 172.48, 143.29, 136.26, 130.46, 125.38, 125.36, 123.27, 123.27, 121.40, 53.32, 47.77, 36.91, 35.14, 29.94, 25.46, 24.83. LCMS (ESI): m/z [M+H]$^+$: calcd. for $C_{22}H_{32}N_3O_4$: 402.2382; found: 402.2387.

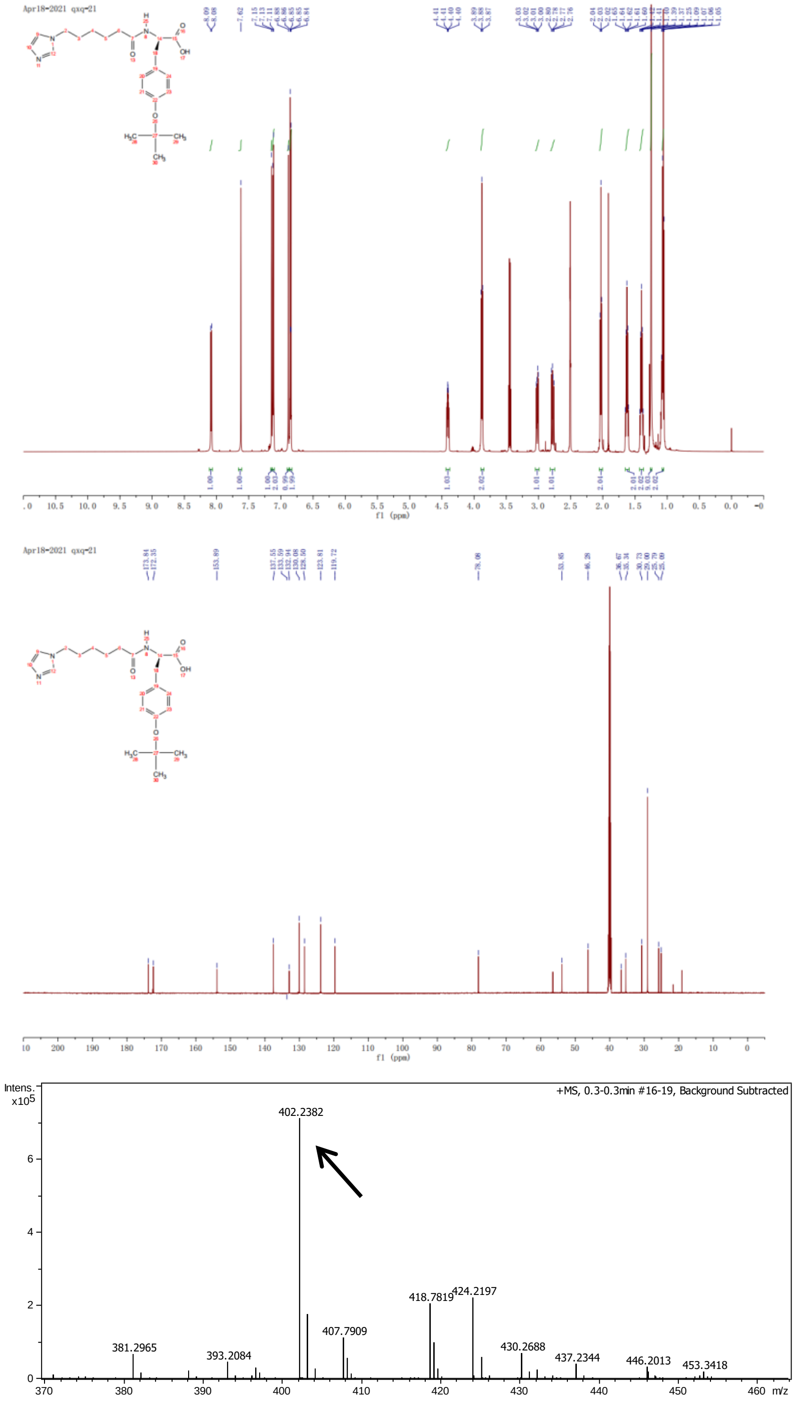

Figure S28. ^{1}H-NMR, ^{13}C-NMR and LCMS spectra of **2p**.

2q: Colorless solid (86% yield), ^{1}H-NMR (600 MHz, DMSO-d$_6$) δ 8.22 (d, *J* = 6 Hz, 1H), 8.12 (m, 2H), 8.09 (s, 1H), 7.72-7.70 (t, *J* = 12 Hz, 2H), 7.59-7.56 (t, *J* = 18 Hz, 1H), 7.17 (s, 1H), 6.93 (s, 1H), 4.54-4.50 (m, 1H), 3.89-3.87 (t, *J* = 12 Hz, 2H), 3.24-3.22 (m, 1H), 3.00-2.96 (m, 1H), 2.04-2.02 (m, 2H), 1.62-1.60 (m, 2H), 1.40-1.37 (m, 2H), 1.06-1.02 (m, 2H). ^{13}C-NMR (151 MHz, DMSO-d$_6$) δ173.28, 172.44, 148.00, 140.75, 137.42, 136.63, 130.05, 128.03, 124.41, 121.92, 119.86, 53.31, 46.38, 36.68, 35.36, 30.66, 25.76, 25.05. LCMS (ESI): m/z [M+H]$^+$: calcd. for $C_{18}H_{23}N_4O_5$: 375.1663; found: 375.1663.

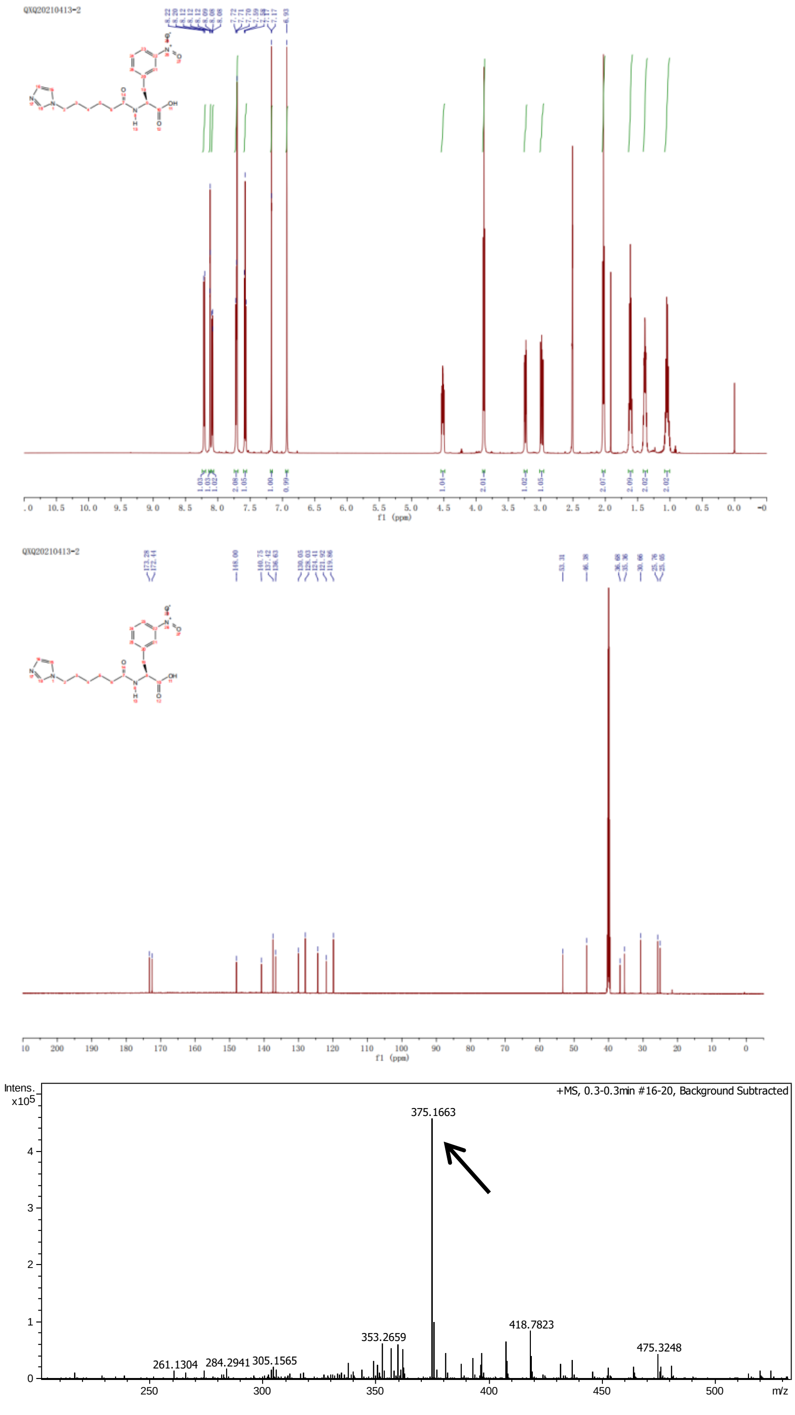

Figure S29. ^{1}H-NMR, ^{13}C-NMR and LCMS spectra of **2q**.

2r: Colorless solid (79% yield), ^{1}H-NMR (600 MHz, DMSO-d_6) δ 8.26 (d, *J* = 6 Hz, 1H), 7.97-7.95 (m, 2H), 7.63 (t, *J* = 6 Hz, 1H), 7.51-7.48 (m, 2H), 7.29 (s, 1H), 7.06 (s, 1H), 4.63-4.60 (m, 1H), 3.94-3.92 (t, *J* = 12 Hz, 2H), 3.50-3.47 (m, 1H), 3.04-3.00 (m, 1H), 2.03-1.97 (m, 2H), 1.67-1.62 (m, 2H), 1.40-1.35 (m, 2H), 1.08-1.02 (m, 2H). ^{13}C-NMR (151 MHz, DMSO-d_6) δ173.25, 172.34, 149.67, 137.13, 133.50, 133.46, 132.78, 128.58, 126.73, 125.00, 120.31, 52.06, 46.81, 35.25, 34.37, 30.46, 25.69, 24.87. LCMS (ESI): m/z $[M+H]^+$: calcd. for $C_{18}H_{23}N_4O_5$: 375.1659; found: 375.1663.

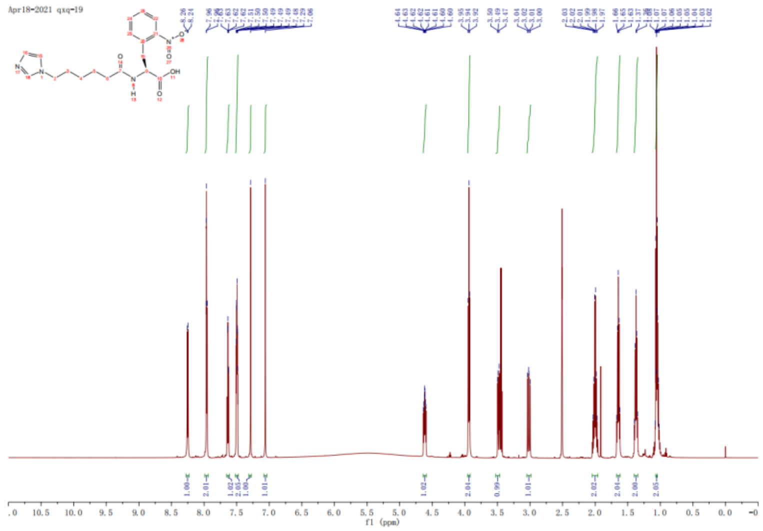

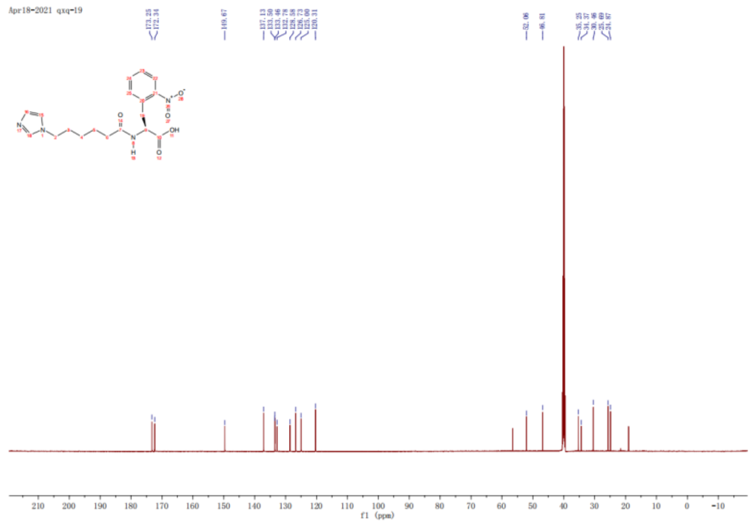

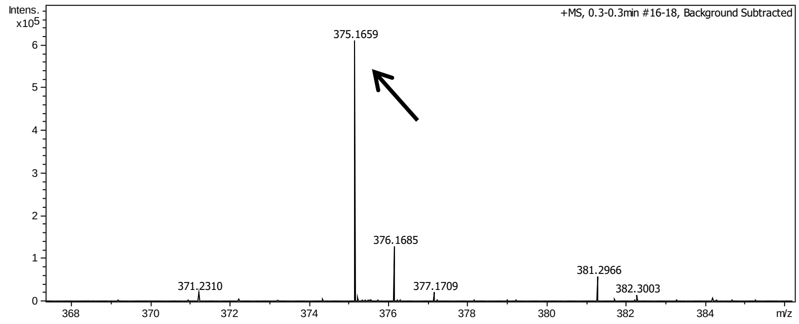

Figure S30. ^{1}H-NMR, ^{13}C-NMR and LCMS spectra of **2r**.

2s: Colorless solid (86% yield), ^{1}H-NMR (600 MHz, DMSO-d_6) δ 9.15 (s, 1H), 8.18-8.16 (d, J = 12 Hz, 1H), 7.76 (s, 1H), 7.67 (s, 1H), 7.15-7.13 (t, J = 18 Hz, 1H), 7.04-7.00 (m, 3H), 4.42-4.38 (m, 1H), 4.14-4.11 (t, J = 18 Hz, 2H), 3.02-2.99 (m, 1H), 2.82-2.78 (m, 1H), 2.25 (s, 3H), 2.08-2.05 (m, 2H), 1.76-1.73 (m, 2H), 1.46-1.43 (m, 2H), 1.14-1.08 (m, 2H). ^{13}C-NMR (151 MHz, DMSO-d_6) δ173.64, 172.43, 138.14, 137.52, 135.56, 130.22, 128.47, 127.46, 126.60, 122.34, 120.39, 53.81, 48.66, 37.13, 35.12, 29.59, 25.35, 24.84, 21.49. LCMS (ESI): m/z [M+H]$^{+}$: calcd. for $C_{22}H_{26}N_3O_3$: 344.1973; found: 344.1969.

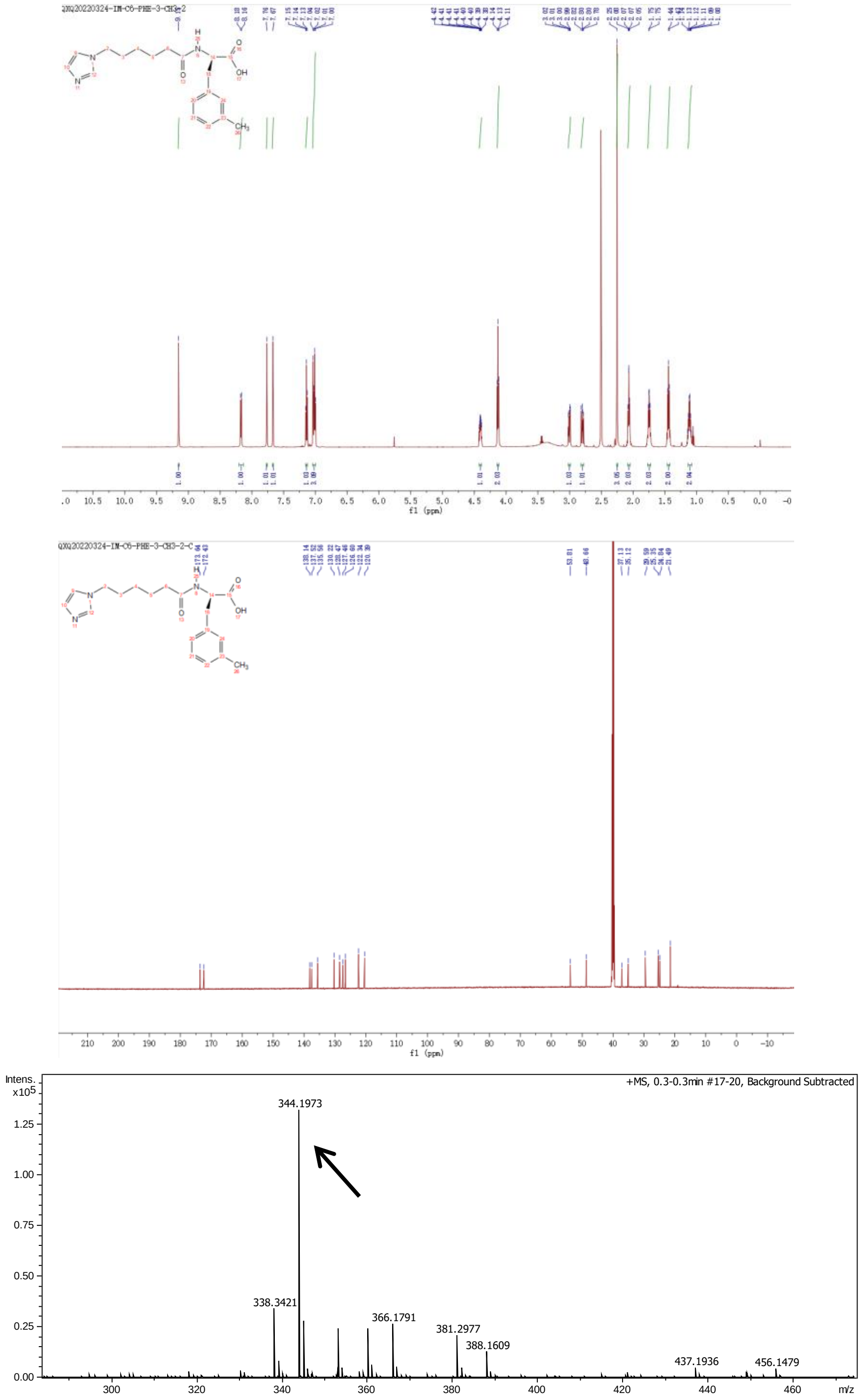

Figure S31. ^{1}H-NMR, ^{13}C-NMR and LCMS spectra of **2s**.

2t: Colorless solid (86% yield), ^{1}H-NMR (600 MHz, DMSO-d_6) δ 8.20-8.19 (d, J = 6 Hz, 1H), 7.83 (s, 1H), 7.23 (s, 1H), 7.16-7.07 (m, 4H), 6.99 (s, 1H), 4.46-4.42 (m, 1H), 3.92-3.90 (t, J = 12 Hz, 2H), 3.09-3.06 (m, 1H), 2.82-2.78 (m, 1H), 2.29 (s, 3H), 2.07-2.01 (m, 2H), 1.67-1.62 (m, 2H), 1.43-1.39 (m, 2H), 1.11-1.02 (m, 2H). ^{13}C-NMR (151 MHz, DMSO-d_6) δ173.91, 172.45, 137.28, 136.52, 136.42, 130.45, 130.05, 127.39, 126.91, 126.01, 120.10, 52.32, 46.63, 35.27, 34.85, 30.57, 25.73, 24.98, 19.40. LCMS (ESI): m/z [M+H]$^{+}$: calcd. for $C_{22}H_{26}N_3O_3$: 344.1973; found: 344.1969.

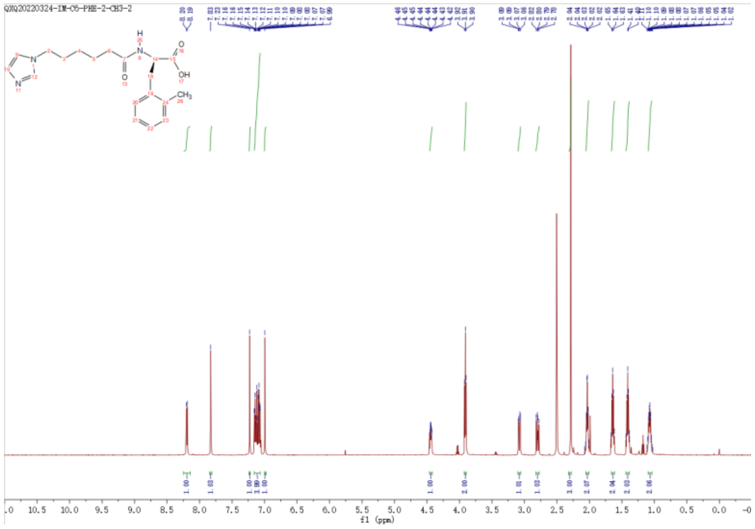

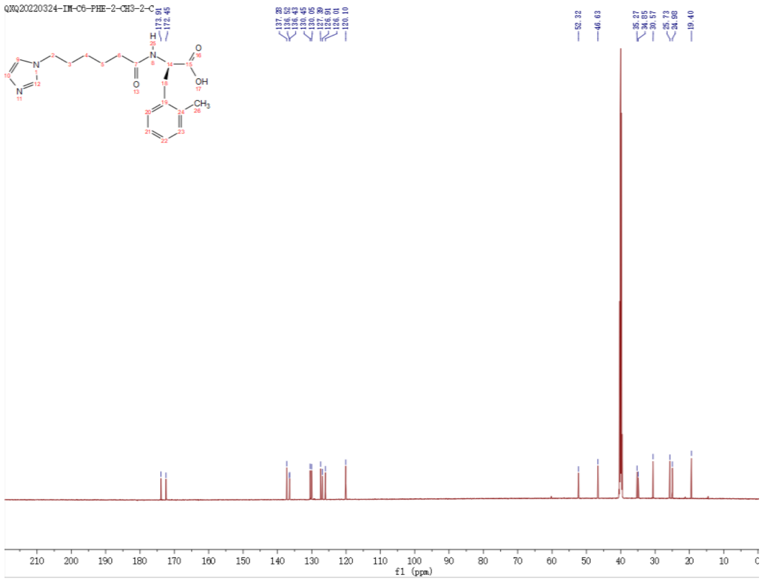

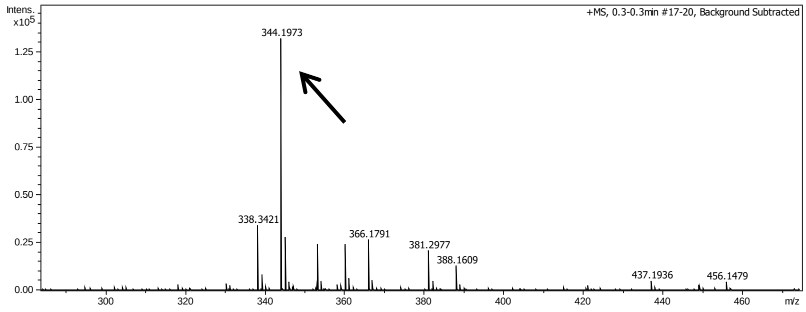

Figure S32. ^{1}H-NMR, ^{13}C-NMR and LCMS spectra of **2t**.

2w: Colorless solid (88% yield), ^{1}H-NMR (600 MHz, DMSO-d6) δ 8.21 (d, J = 12 Hz, 1H), 7.87 (d, J = 6 Hz, 1H), 7.83-7.81 (m, 2H), 7.73 (s, 1H), 7.65 (s, 1H), 7.48-7.41 (m, 3H), 7.11 (s, 1H), 6.92 (s, 1H), 4.60-4.56 (m, 1H), 3.76 (t, J = 12 Hz, 2H), 3.26-3.23 (m, 1H), 3.04-3.00 (m, 1H), 2.04 (t, J = 12 Hz, 2H), 1.53-1.49 (m, 2H), 1.38-1.34 (m, 2H), 1.01-0.95 (m, 2H). ^{13}C-NMR (151 MHz, DMSO-d6) δ173.73, 172.49, 172.44, 137.37, 135.99, 133.41, 132.31, 128.14, 128.00, 127.92, 127.91, 127.86, 126.44, 125.91, 119.33, 53.72, 46.32, 37.49, 35.32, 30.58, 25.67, 25.02, 21.57. LCMS (ESI): m/z [M+H]$^+$: calcd. for $C_{22}H_{26}N_3O_3$: 380.1972; found: 380.1969.

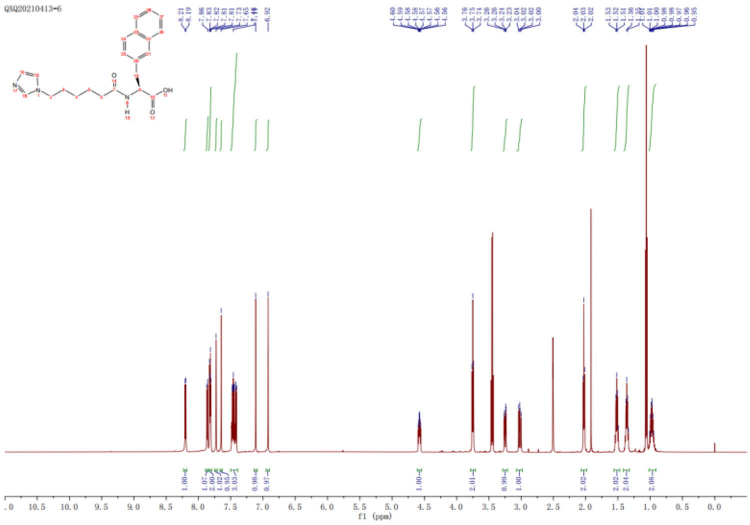

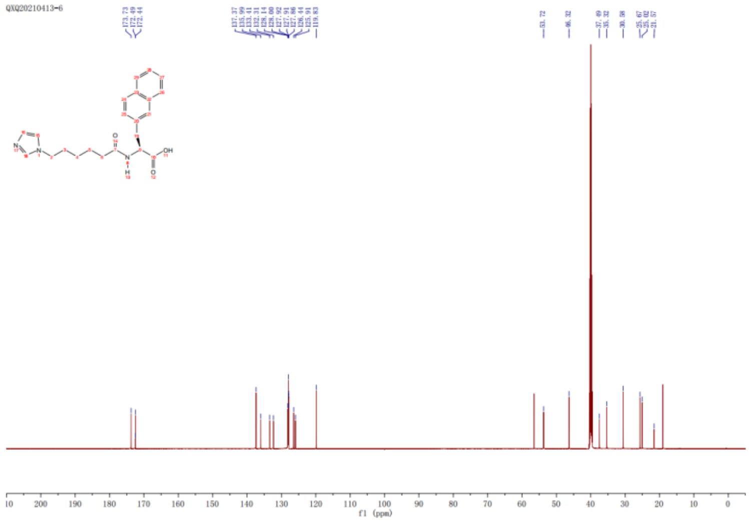

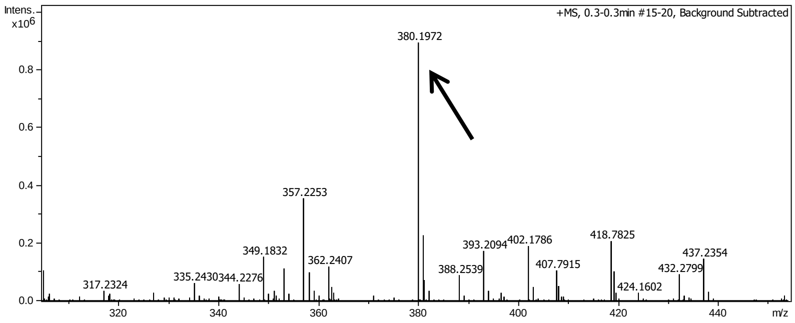

Figure S33. ^{1}H-NMR, ^{13}C-NMR and LCMS spectra of **2u**.

Typical chiral GC analyses for the epoxidation of styrene

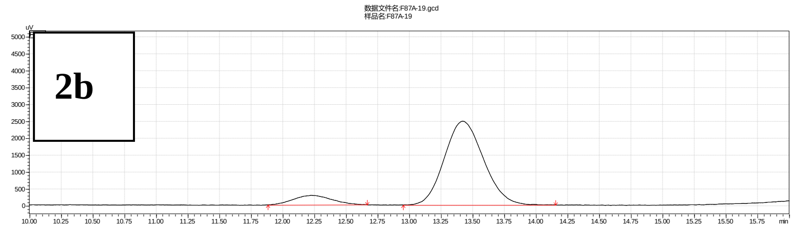

RT (min)	12.225 (*S*)	13.421 (*R*)
Area	4617	53226
ee % (*R*)	84	

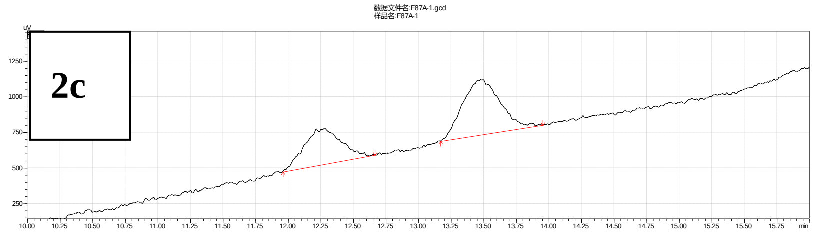

RT (min)	12.28 (*S*)	13.478 (*R*)
Area	5009	7760
ee % (*R*)	22	

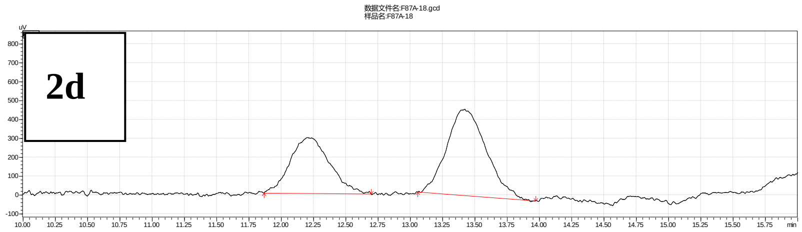

RT (min)	12.25 (*S*)	13.427 (*R*)
Area	4016	22419
ee % (*R*)	70	

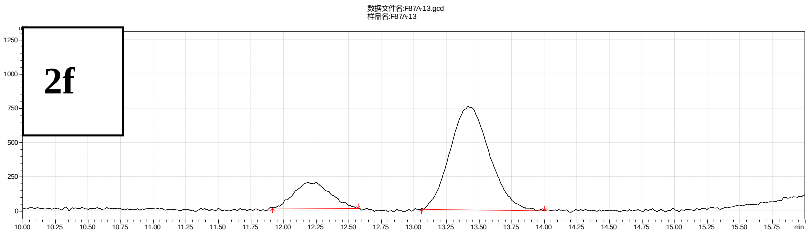

RT (min)	12.253 (*S*)	13.418 (*R*)
Area	3100	15857
ee % (*R*)	67	

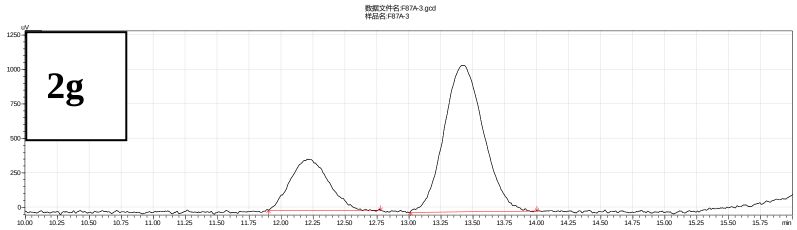

RT (min)	12.211 (*S*)	13.417 (*R*)
Area	7099	22643
ee % (*R*)	52	

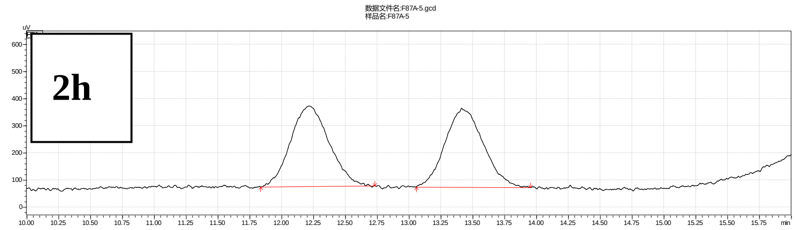

RT (min)	12.216 (*S*)	13.414 (*R*)
Area	5610	6046
ee % (*R*)	4	

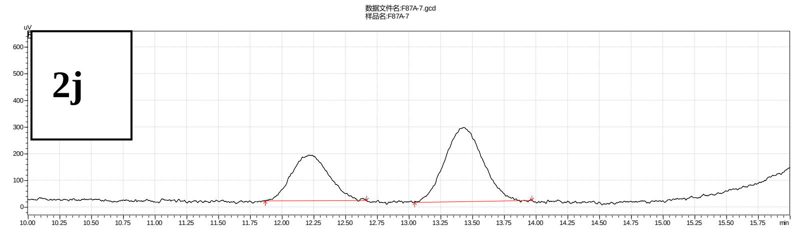

RT (min)	12.212 (*S*)	13.433 (*R*)
Area	3478	5730
ee % (*R*)	24	

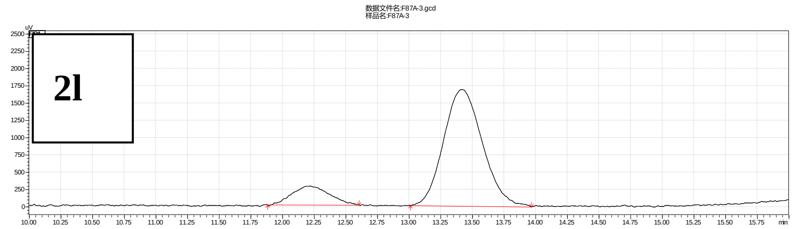

RT (min)	12.220 (*S*)	13.414 (*R*)
Area	4489	35874
ee % (*R*)	77	

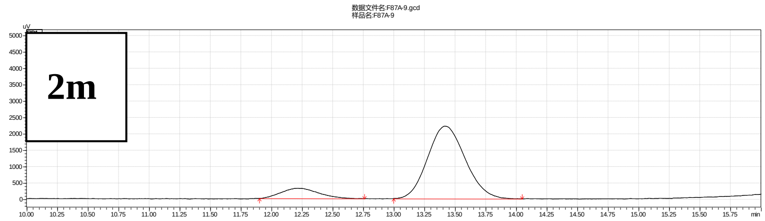

RT (min)	12.216 (*S*)	13.416 (*R*)
Area	4814	47323
ee % (*R*)	82	

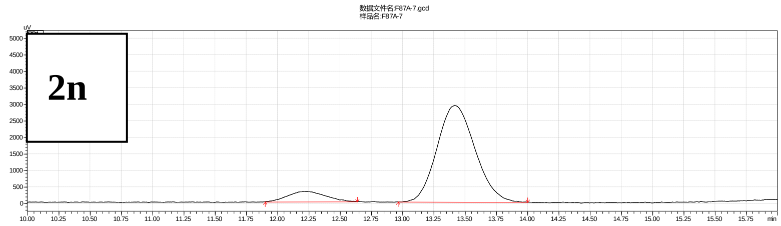

RT (min)	12.212 (*S*)	13.419 (*R*)
Area	5069	61687
ee % (*R*)	85	

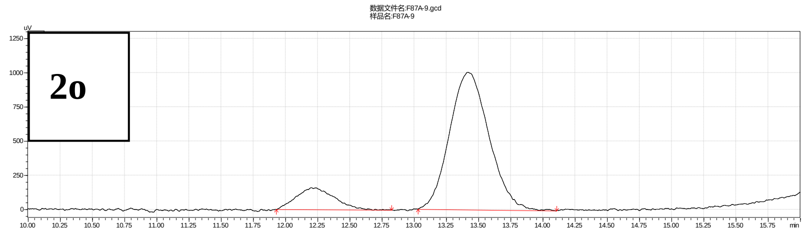

RT (min)	12.202 (*S*)	13.418 (*R*)
Area	2441	21358
ee % (*R*)	79	

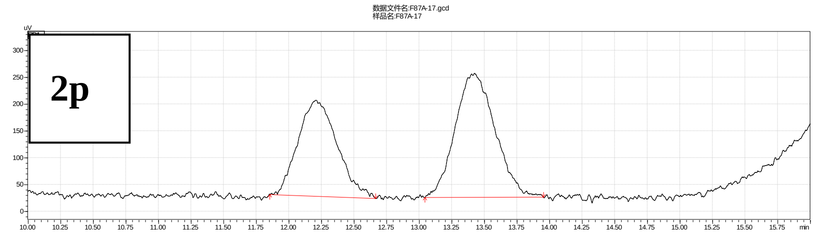

RT (min)	12.210 (*S*)	13.426 (*R*)
Area	3708	4902
ee % (*R*)	14	

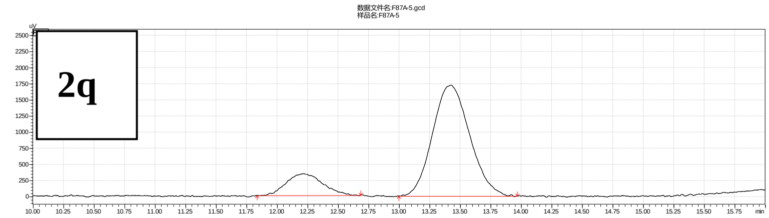

RT (min)	12.218 (*S*)	13.427 (*R*)
Area	5202	36358
ee % (*R*)	75	

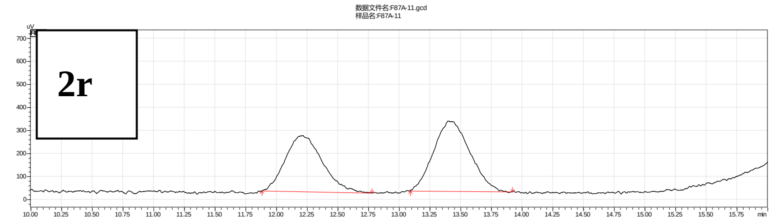

RT (min)	12.216 (*S*)	13.400 (*R*)
Area	5090	6254
ee % (*R*)	13	

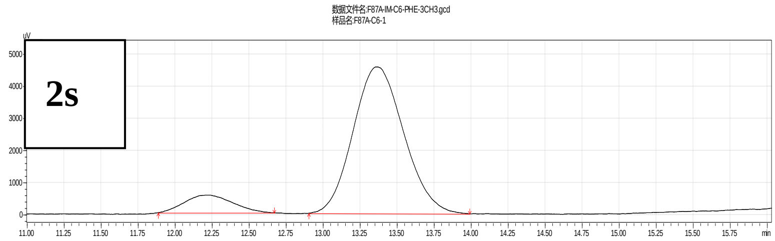

RT (min)	12.216 (*S*)	13.400 (*R*)
Area	10395	108720
ee % (*R*)	83	

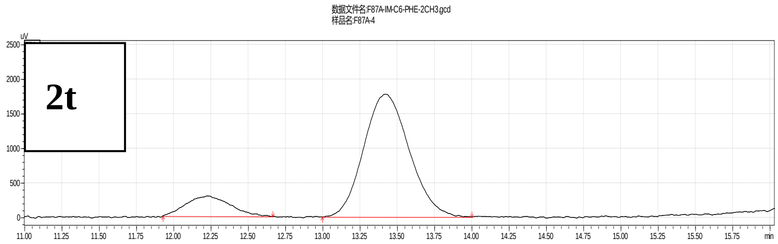

RT (min)	12.216 (*S*)	13.400 (*R*)
Area	6024	36628
ee % (*R*)	71	

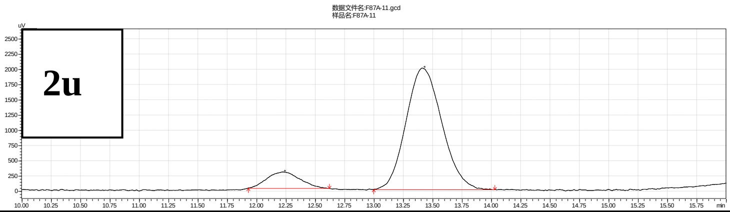

RT (min)	12.222 (*S*)	13.411 (*R*)
Area	3436	42291
ee % (R)	85	

Typical chiral GC analyses for the hydroxylation of ethylbenzene

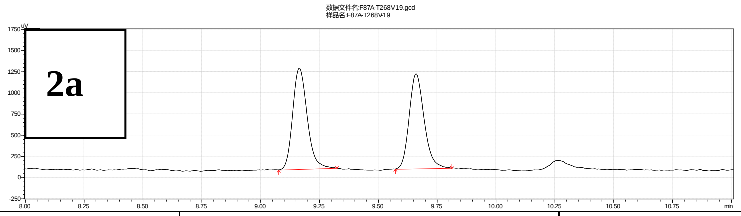

RT (min)	9.165 (*R*)	9.661 (*S*)
Area	5117	4942
ee % (*R*)	2	

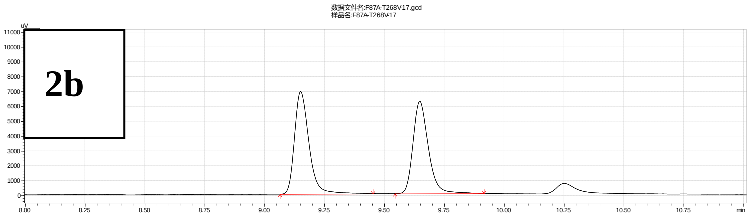

RT (min)	9.151 (*R*)	9.648 (*S*)
Area	28878	27453
ee % (*R*)	3	

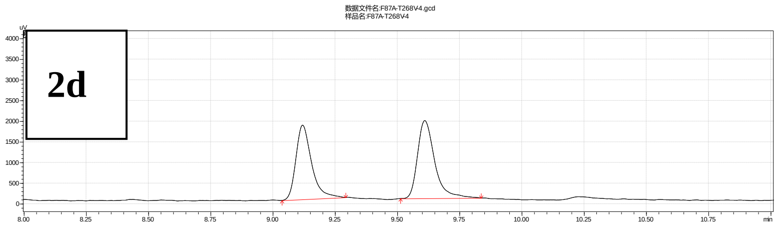

RT (min)	9.120 (*R*)	9.611 (*S*)
Area	7889	9027
ee % (*R*)	-7	

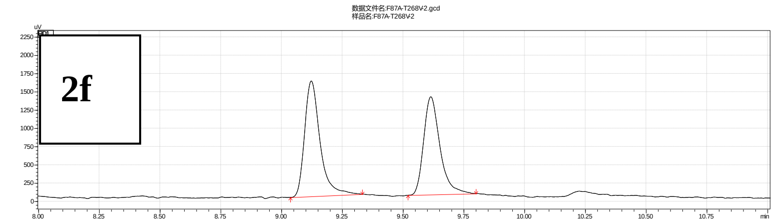

RT (min)	9.123 (*R*)	9.615 (*S*)
Area	6997	6235
ee % (*R*)	6	

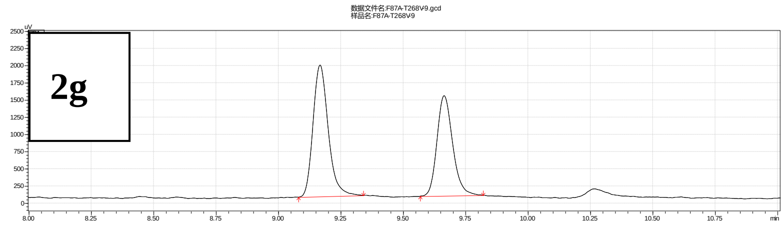

RT (min)	9.167 (*R*)	9.664 (*S*)
Area	8105	6378
ee % (*R*)	12	

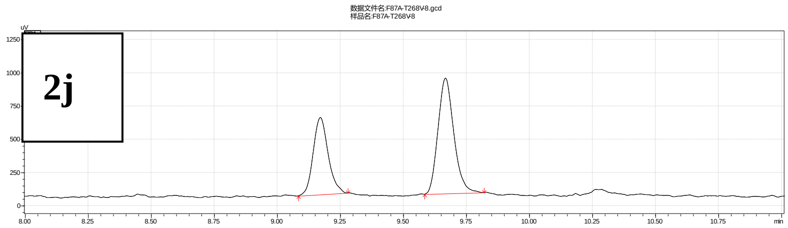

RT (min)	9.039 (*R*)	9.515 (*S*)
Area	1878	3382
ee % (*R*)	-29	

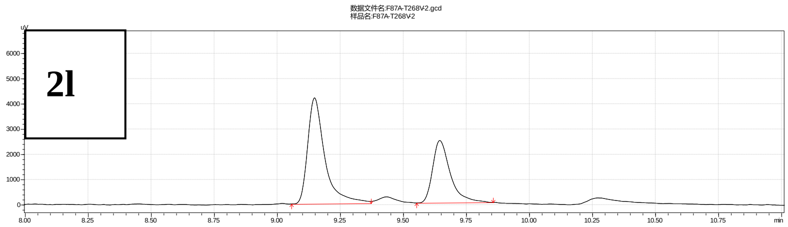

RT (min)	9.149 (*R*)	9.645 (*S*)
Area	19934	12004
ee % (*R*)	25	

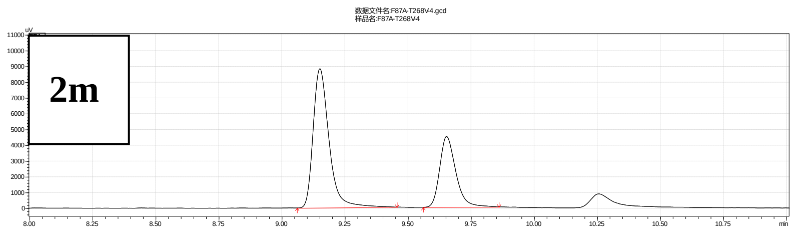

RT (min)	9.151 (*R*)	9.653 (*S*)
Area	38610	19817
ee % (*R*)	32	

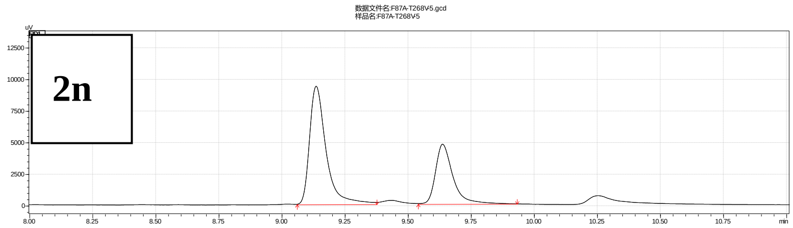

RT (min)	9.136 (*R*)	9.637 (*S*)
Area	42733	22681
ee % (*R*)	31	

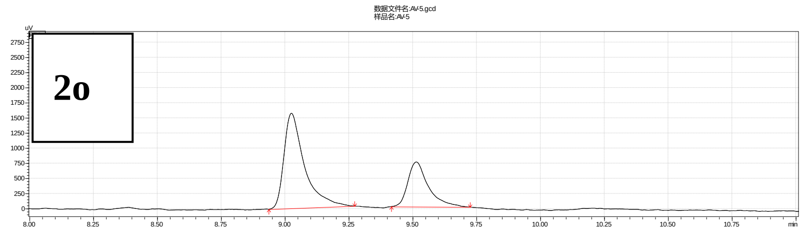

RT (min)	9.026 (*R*)	9.515 (*S*)
Area	8773	4129
ee % (*R*)	36	

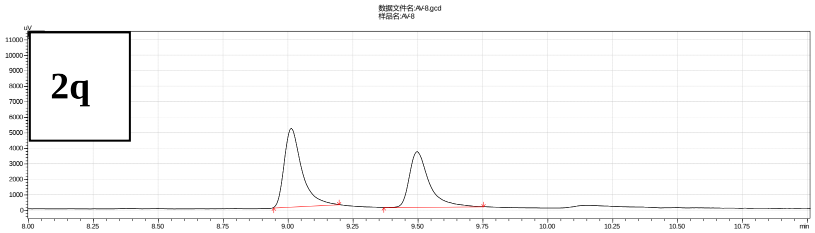

RT (min)	9.168 (*R*)	9.666 (*S*)
Area	16733	11166
ee % (*R*)	12	

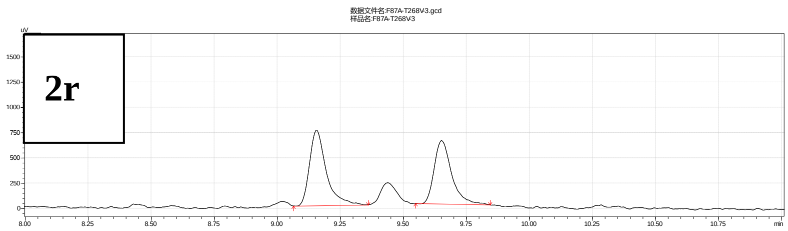

RT (min)	9.156 (*R*)	9.652 (*S*)
Area	3427	2992
ee % (*R*)	7	

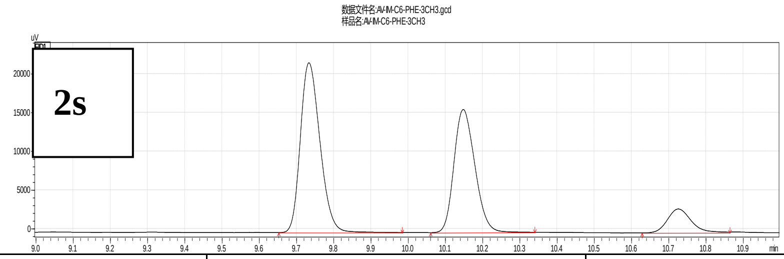

RT (min)	9.734 (*R*)	10.149 (*S*)
Area	80224	62027
ee % (*R*)	13	

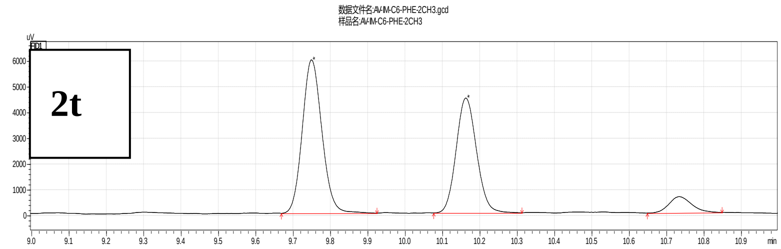

RT (min)	9.75 (*R*)	10.163 (*S*)
Area	21735	16923
ee % (*R*)	12	

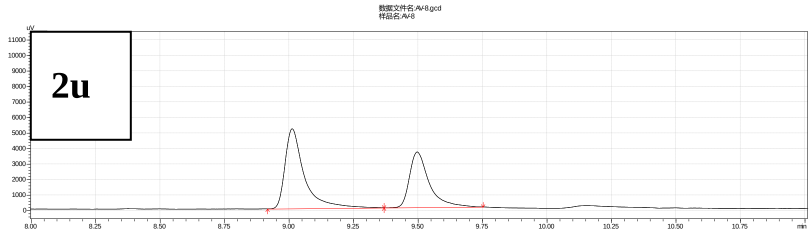

RT (min)	9.013 (*R*)	9.498 (*S*)
Area	26703	18883
ee % (*R*)	17	

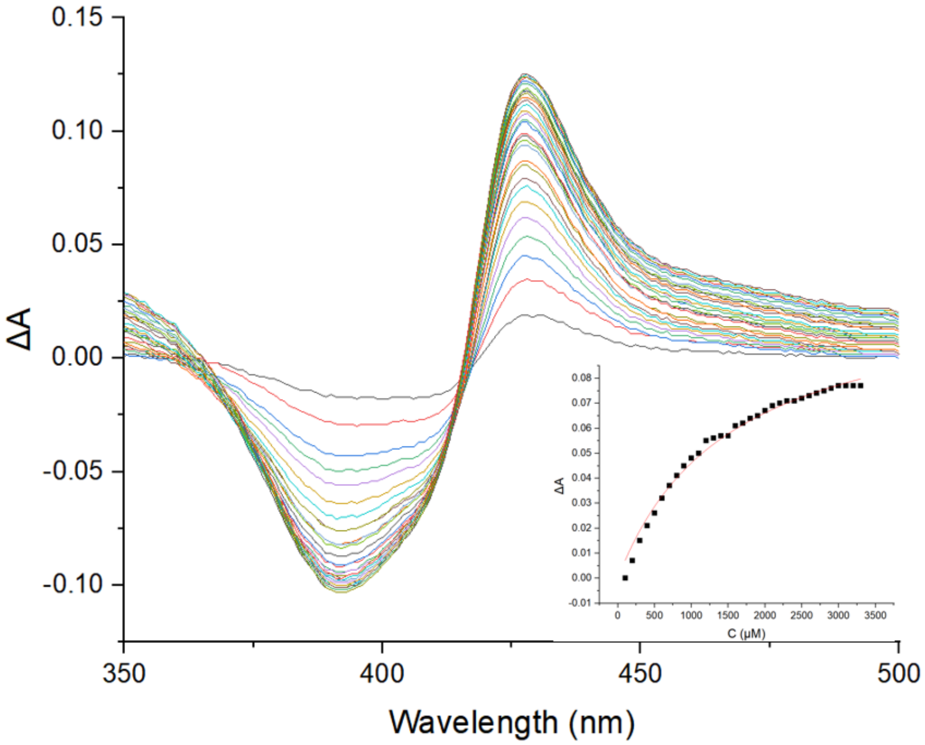

Figure S34. The UV-vis spectral change of F87A upon the addition of 2a (left) and the dissociation constants estimated by the titration experiment (inlet).

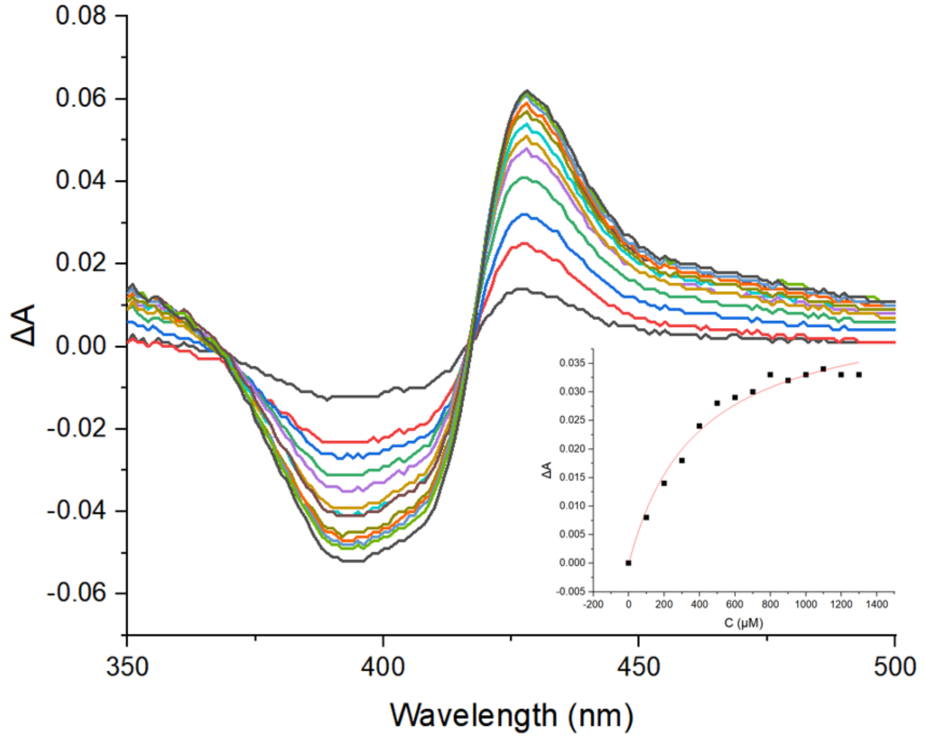

Figure S35. The UV-vis spectral change of F87A upon the addition of 2k (left) and the dissociation constants estimated by the titration experiment (inlet).

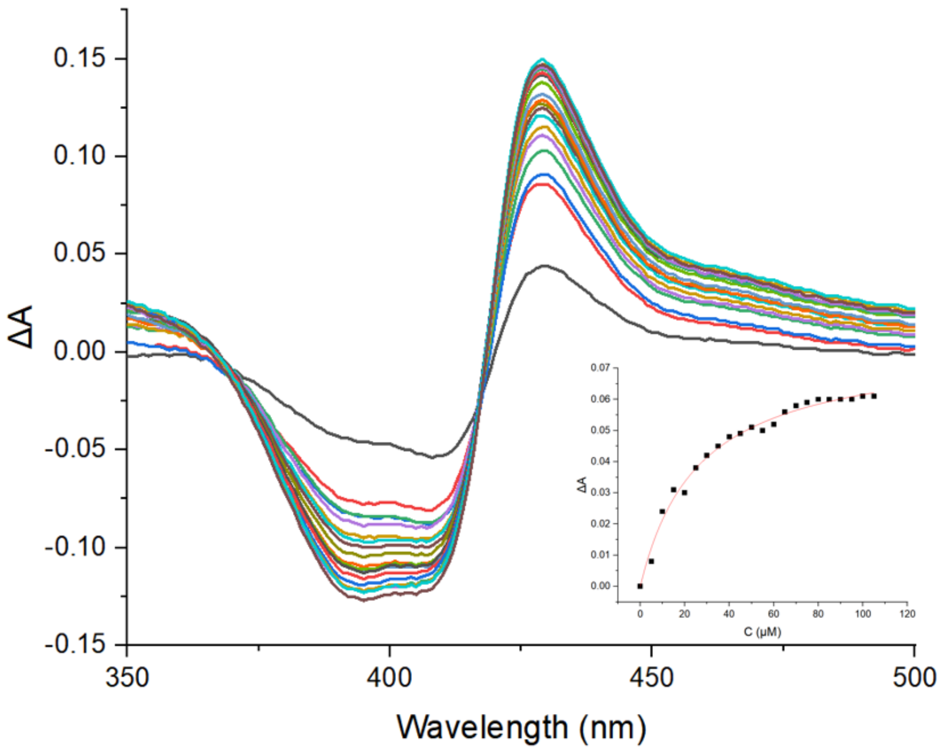

Figure S36. The UV-vis spectral change of F87A upon the addition of 2n (left) and the dissociation constants estimated by the titration experiment (inlet).

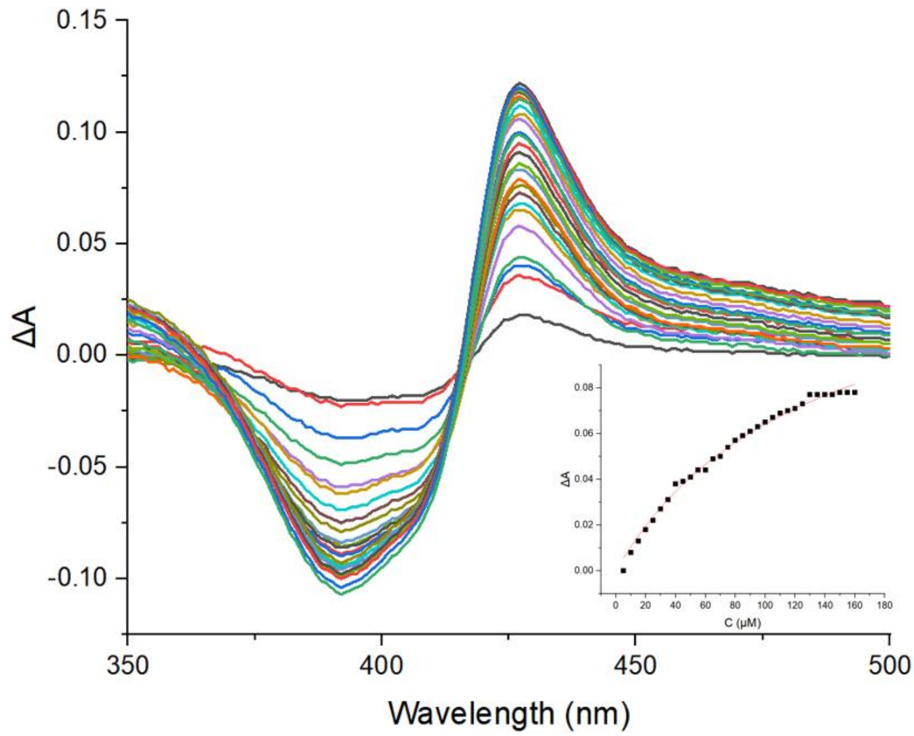

Figure S37. The UV-vis spectral change of F87A upon the addition of 2s (left) and the dissociation constants estimated by the titration experiment (inlet).

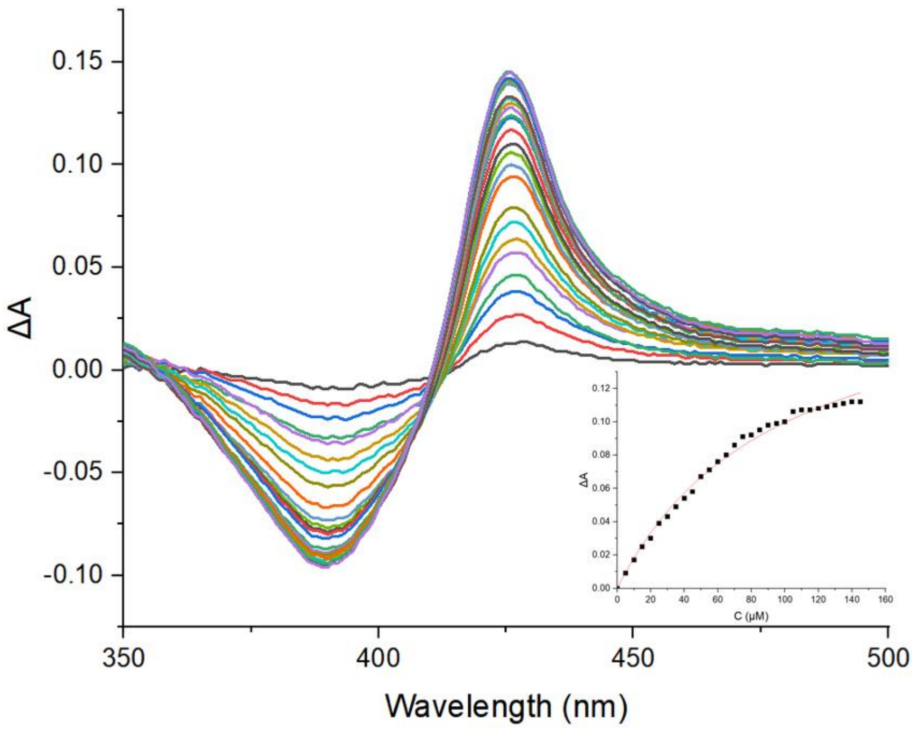

Figure S38. The UV-vis spectral change of F87A upon the addition of Im-C6-Phe (left) and the dissociation constants estimated by the titration experiment (inlet).
